# Discovery of a Structurally Distinct Acetylenase in the Biosynthesis of Mangotoxin

**DOI:** 10.1021/jacs.5c21680

**Published:** 2026-04-28

**Authors:** Edward D. Badding, Elijah N. Kissman, Stefan V. Velculescu, Michelle C. Y. Chang

**Affiliations:** Department of Chemistry, 6740 Princeton University, Princeton, New Jersey 08544, United States

## Abstract

Amino acids, peptides, and proteins play pivotal roles in medicine, materials, and catalysis, with their functions largely dictated by their side-chain functionality. Recent studies have shown that heme oxygenase-like domain-containing oxidases (HDOs) catalyze a broad range of interesting chemical reactions on amino acids and their derivatives to produce structurally diverse target structures. Using a bioinformatics approach, the mangotoxin biosynthetic operon was found to house an unannotated HDO, MboA, along with a dedicated redox partner, MboB. We show that MboA catalyzes alkyne formation by iterative desaturations on the side-chain of a peptide substrate, where the transformation between alkene and alkyne is gated by MboB. The crystal structure of Fe­(II)_2_-MboA reveals an unexpectedly short Fe–Fe distance, suggesting that the activation of strong C­(sp^2^)–H bonds may utilize a mechanism distinct from other HDOs and expands the scope of known HDO chemistry.

## Graphical abstract

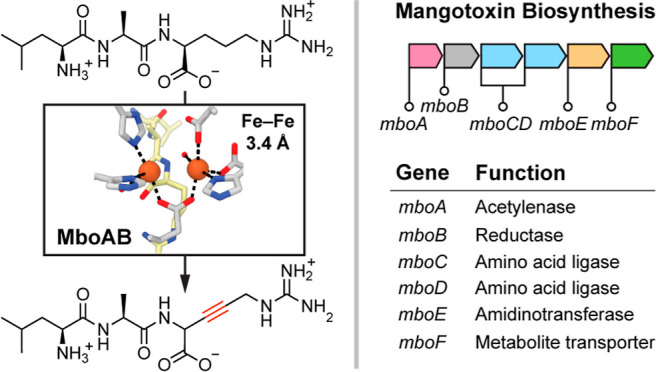

## Introduction

Amino acids and peptides form an important group of compounds used in a wide range of fields such as medicine,^1,2^ materials,^3^ and catalysis.^4,5^ While the 20 proteinogenic amino acids provide a surprisingly large scope of biological function, many of these synthetic applications require side-chain structures with greater functional group diversity. As such, there is great interest in the development of synthetic^6^ and biocatalytic^7^ approaches to prepare and incorporate noncanonical amino acids into target structures to be used as chemical warheads,^8^ probes,^9^ or downstream handles for further elaboration.^10^ Toward this goal, our group^11–13^ and others^14–18^ have been interested in the discovery and characterization of new enzymes that modify amino acids and peptides to discover new ways to introduce unusual chemical structures onto the side chain of amino acids.

We have been particularly interested in metalloenzymes that can activate C­(sp^3^) positions,^11,12,19^ with a growing interest in the heme oxygenase-like domain-containing oxidases (HDOs). HDOs share an α-helical bundle fold characteristic of heme oxygenases, but instead utilize a nonheme transition metal active site.^20^ A bimetallic Fe–Fe cluster^19,21–29^ is the most common active site found, but Fe and Fe–Mn cofactors are also supported.^20,30^ Interestingly, HDOs appear to specialize in biosynthetic pathways that utilize amino acid substrates, activating O_2_ to catalyze a broad range of reactions, such as O atom transfer,^31–35^ methine excision,^36^ lyase-dependent desaturation,^11,36^ C–C scission,^37^ nitrile formation,^25,26^ and azetidine ring generation^28,29^ (Figure 1A).

**Figure 1:**
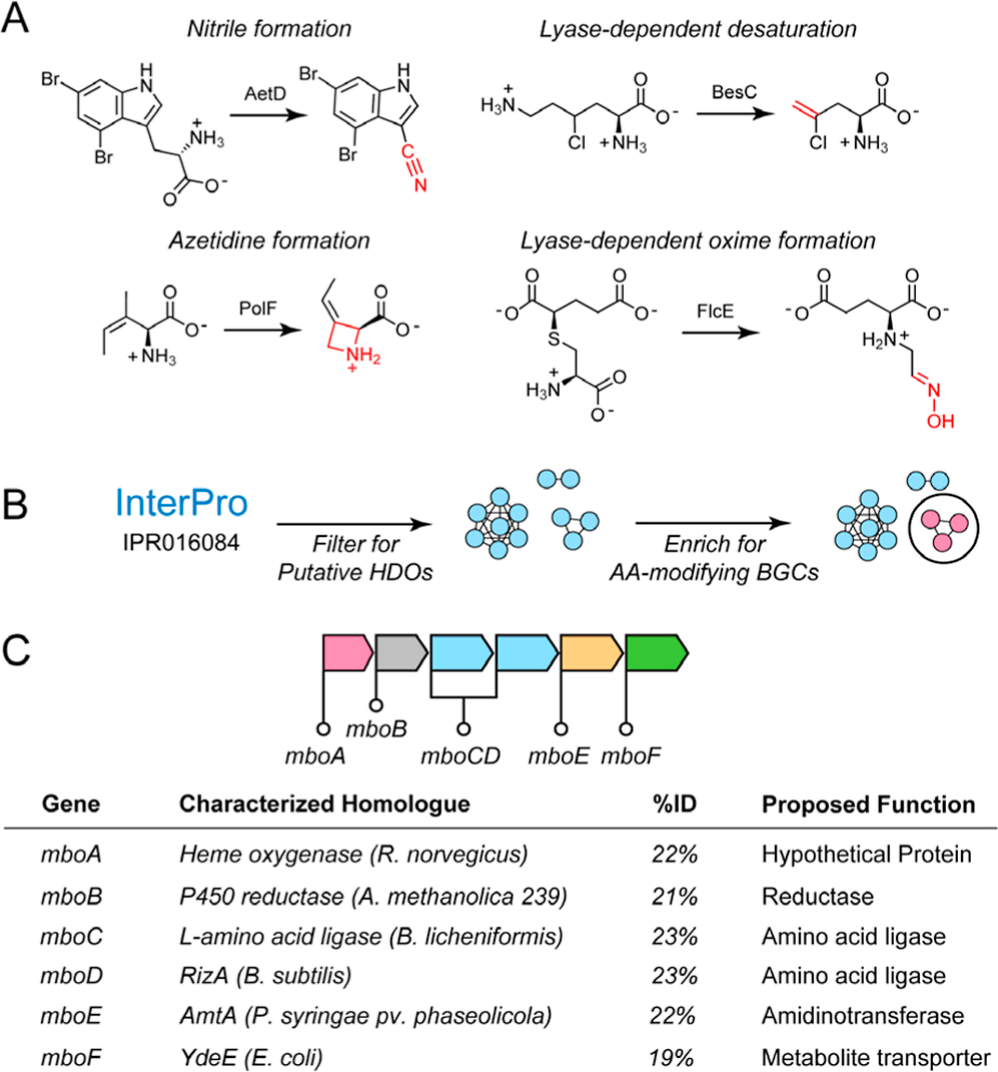
Heme oxygenase-like domain containing oxidase (HDO) family. (A) Examples of chemical transformations mediated by HDOs. (B) Strategy used to identify HDO-containing BGCs that modify amino acids. (C) The mangotoxin BGC consists of five enzymes, including the unannotated HDO, MboA, and two amino acid ligases from the ATP-grasp family, MboC and MboD.

To focus our search for HDO candidates that modify amino acids and peptides, we searched for sequences that colocalize with other enzymes known to work on amino acid-derived substrates (Figure 1B). For this purpose, we identified HDO sequences within biosynthetic gene clusters (BGCs) that contain amino-acid ligases from the ATP-grasp family. This enzyme family is known to construct short oligopeptides, providing a simple strategy to produce small peptide natural products without the utilization of the more complex ribosomal or nonribosomal peptide synthase machinery.^38–41^

Within this list, we found the BGC that encodes the biosynthesis of mangotoxin, a plant antagonist proposed to inhibit ornithine *N*-acetyl transferase.^42^ Although the mangotoxin BGC was not documented to contain an HDO, prior work established that the mangotoxin gene cluster consists of six genes: *mboA*, *mboB*, *mboC*, *mboD*, *mboE*, and *mboF*.^43^ Through a combination of structural and biochemical studies, we show that this cluster produces a tripeptide as a substrate for MboA, an HDO that catalyzes the four-electron desaturation of two inert C­(sp^3^) sites to form an internal alkyne between C_β_ and C_γ_ of the C-terminal residue. This is a challenging transformation that has not been previously observed in HDOs and requires a dedicated redox partner, MboB, that gates the transformation from an alkene to an alkyne. Notably, the crystal structure of MboA reveals an unexpectedly short Fe–Fe distance, suggesting that alkyne formation may utilize a mechanism distinct from other HDOs.

## Results

### Bioinformatic Analysis of HDOs and Identification of MboA as an HDO

Given that many previously studied HDOs act on amino acids or amino acid-derived substrates,^11,25,26,28,29,31,32,35–37^ we hypothesized that a subset of HDOs may colocalize with freestanding l-amino acid ligases and thus be amenable toward more facile full pathway reconstitution. Using a sequence and structure-guided approach (AlphaFold),^23,30,44^ we analyzed members of the InterPro family IPR016084 (76,090 sequences)—which contain a broad range of annotations including HDOs—and filtered for sequences that likely bind a binuclear metallocofactor to identify putative HDOs (at least 19,778 sequences) (Figures 1B and S2B). We then examined their genome neighborhood^45^ to find HDOs located nearby at least one ATP-grasp ligase (1447 sequences). Within these, we observed at least 25 unique BGCs that feature an HDO and at least one ATP-grasp ligase (Figure S2C).

The mangotoxin BGC (*mboABCDEF*) was found within this set and we identified MboA as an unannotated HDO (Figure 1C). While the final product of this gene cluster was unknown, previous studies^42,43^ suggested that it is a short oligopeptide that exhibits antimicrobial and herbicidal properties. We therefore initiated in vitro studies on the *mbo* BGC to elucidate its product and characterize the reaction catalyzed by MboA.

### In Vitro Characterization of MboABCDE

The gene sequences encoding MboA, MboB, MboC, MboD, and MboE were codon optimized and inserted into pET16b expression vectors with an N-terminal His_10_-tag. All five proteins were then heterologously expressed in *Escherichia coli* BL21­(DE3) and purified by metal-affinity chromatography (Figure S3). As the product of the *mbo* pathway was expected to be a tripeptide, the two amino acid ligases, MboC and MboD, were first screened against the 20 canonical amino acids and l-ornithine (Orn) to give insight into the identity of the mangotoxin building blocks. MboC showed a clear preference for producing a species with [M + H]^+^ = 246.1531, consistent with three dipeptides: Ala/Arg (*m*/*z*_calc_ = 246.1561), Asn/Leu (*m*/*z*_calc_ = 246.1449), Gln/Val (*m*/*z*_calc_ = 246.1449) (Figure S4A). Analysis of the fragmentation pattern led us to assign the MboC product as the Ala–Arg dipeptide (Figure S4B). MboD produced a range of putative dipeptide products at low signal intensity, suggesting that it may be responsible for tripeptide production (Figure S4C). Screening of MboC and MboD together resulted in the formation of several tripeptides of which the most abundant were comprised of Leu/Ile, Ala, and Arg (Figure S4D).

We next determined the product of the MboE, a putative amidinotransferase, by addition of the MboCD screening assay. A product corresponding to replacement of an NH group with O ([M + H] = 360.2217) was observed only in the presence of MboE, consistent with the conversion of the Leu-Ala-Arg tripeptide (LAR) to Leu-Ala-Citrulline (Cit) (Figure S5A,B). Subsequent MS/MS experiments confirmed this amino acid sequence (Figure S5C). Thus, MboE functions as an arginine deiminase, rather than as a closely related amidinotransferase,^46^ which is consistent with the steady-state kinetic analysis of MboC and MboD that shows that Arg and Arg-containing peptides are the preferred substrates for these enzymes, rather than free Cit (Figures S6 and S7). Based on these results, we hypothesized that the biosynthesis of mangotoxin likely utilizes Leu, Ala, and Arg as building blocks (Figure 2A,B).

**Figure 2:**
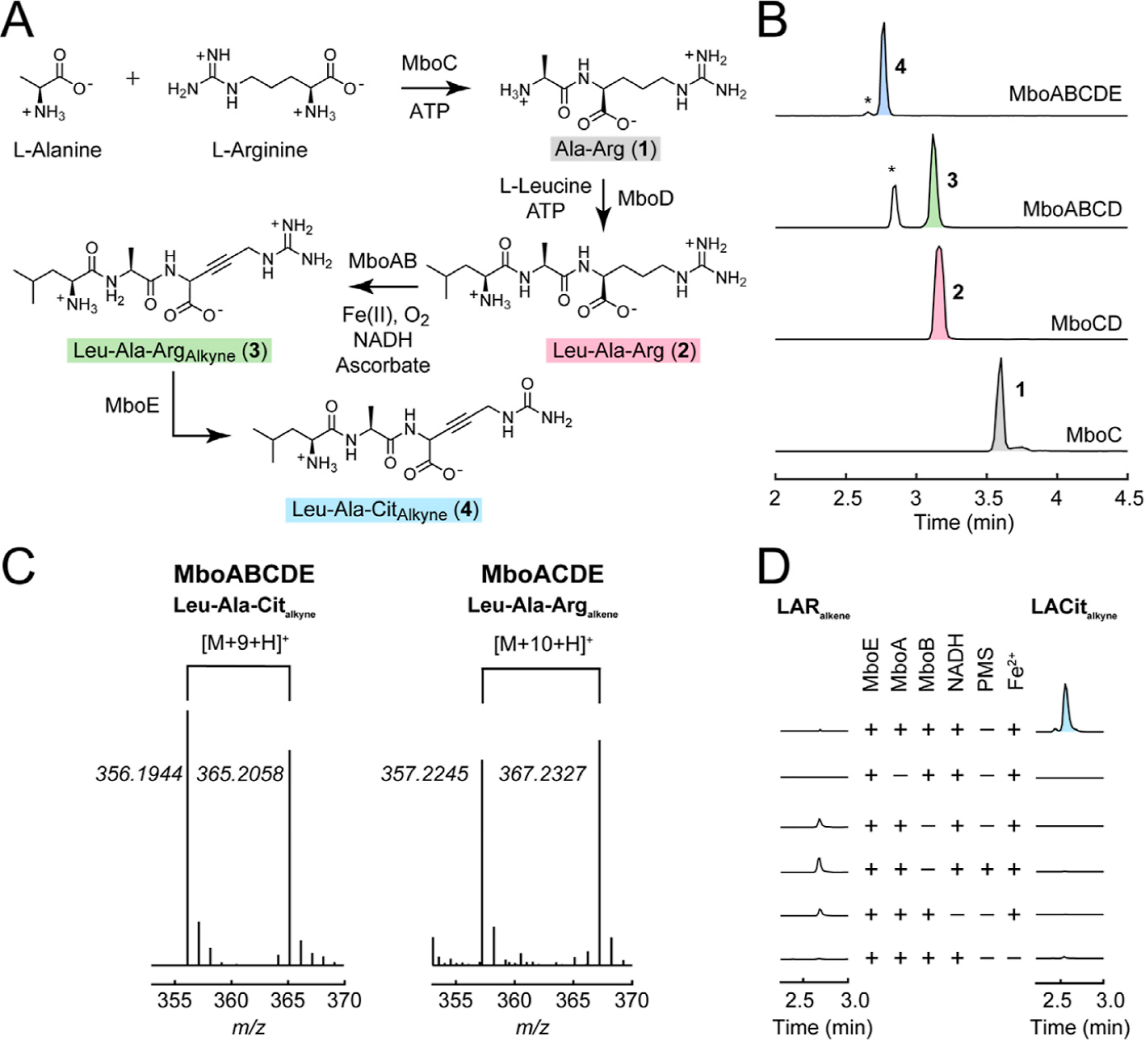
In vitro characterization of the enzymes from the mangotoxin BGC. (A) Biosynthetic pathway proposed for mangotoxin. The stereochemistry of the Arg/Cit residue is unknown after modification by MboAB and the structures of **3** and LAR_alkene_ are proposed based on LC-MS/MS characterization. (B) In vitro reconstitution of MboABCDE. Various Mbo enzymes were added as labeled to in vitro reconstitution assays with 1 mM amino acids (Leu, Ala, Arg), 5 mM ATP, 5 mM MgCl_2_, 1 mM ascorbic acid, 100 μM ferrous ammonium sulfate, 2.5 mM NADH (MboA, 10 μM; MboBCDE, 1 μM). Assays were analyzed by LC-QTOF/MS and displayed as normalized extracted ion chromatograms (**1**, Ala–Arg, *m*/*z*_obs_ = 246.1562, *m*/*z*_calc_ = 246.1561; **2**, Leu–Ala–Arg, *m*/*z*_obs_ = 359.2415, *m*/*z*_calc_ = 359.2401; **3**, Leu–Ala–Arg_alkyne_, *m*/*z*_obs_ = 355.2100, *m*/*z*_calc_ = 355.2088; **4**, Leu–Ala–Cit_alkyne_, *m*/*z*_obs_ = 356.1941, *m*/*z*_calc_ = 356.1928). (*, cyclized alkyne-containing tripeptide). (C) Mass spectra of two new species produced from the in vitro reconstitution of MboABCDE (left) and MboACDE (right) using [^15^N_4_, ^13^C_6_]-Arg (M + 10) in the presence of Fe­(II), NADH, and ascorbate. The first species (left) corresponds to −4H from Leu–Ala–Cit, which was assigned to Leu–Ala–Cit_alkyne_ (*m*/*z*_obs_ = 356.1917, *m*/*z*_calc_ = 356.1928). Highlighted is the corresponding [M+9 + H]^+^ species (*m*/*z*_obs_ = 365.2043, *m*/*z*_calc_ = 365.2041), indicating that one of the labeled atoms from Arg was lost due to hydrolysis of the guanidinium group by MboE. When MboB was omitted, a species corresponding to −2H from Leu–Ala–Arg (right) was observed, which was assigned to Leu–Ala–Arg_alkene_ (*m*/*z*_obs_ = 357.2245, *m*/*z*_calc_ = 357.2245). Highlighted is the corresponding [M + 10 + H]^+^ species (*m*/*z*_obs_ = 367.2327, *m*/*z*_calc_ = 367.2328), indicating that all C and N atoms from Arg are retained, suggesting that either MboE does not accept this intermediate or it remains bound to MboA waiting for a second round of oxidation. (D) Testing the requirements for production of Leu–Ala–Cit_alkyne_ (LACit_alkyne_) compared to Leu–Ala–Arg_alkene_ (LAR_alkene_). These assays demonstrate that Fe­(II), MboB, and NADH are required for full conversion to product (LACit_alkyne_), whereas conversion stalls at (LAR_alkene_) in the absence of MboB. MboB cannot be replaced by redox mediators, such as phenazine methosulfate, that can be used to support catalysis in other HDOs.

To identify the transformation catalyzed by the MboA, we reconstituted the full MboABCDE pathway using an assay containing the 20 amino acids Orn, [^15^N_4_, ^13^C_6_]-l-arginine (M + 10), MgATP, Fe­(II), ascorbate, and NADH. Under these conditions, we observed the formation of an intriguing new species consistent with loss of 4H ([M + H]^+^ = 356.1935) from the Leu-Ala-Cit tripeptide, as well as the corresponding [M + 9 + H]^+^ species expected for conversion of Arg to Cit (Figure 2C). Interestingly, if MboB is omitted from this reaction, only trace amounts of a different species consistent with loss of 2H ([M + H]^+^ = 357.2245) from LAR is observed, along with the isotopically labeled [M + 10 + H]^+^ species that indicates that the Arg residue remains intact (Figure 2D). MboB is a member of a structurally distinct class of reductases that facilitate one-electron transfer using NAD­(P)H along with a flavin cofactor and Fe_2_S_2_ cluster (Figure S9) and thus appears to be required as a redox partner for MboA.^47–50^

Taken together, these observations support the hypothesis that MboAB acts on LAR to carry out two sequential 2-electron oxidations followed by hydrolysis of the guanidinium group of the Arg residue (Figures 2A,B and S10A). Indeed, oxidation by MboA occurs only when LAR is provided and not Leu–Ala–Cit (Figure S10B), which is consistent with the in vitro reconstitution of MboABCDE and the characterization of MboCD and MboE. The formation of the 4-electron oxidized species was found to be MboA-, MboB-, NADH-, and Fe-dependent, while formation of the 2-electron oxidized species can be produced by MboA and Fe­(II) alone (Figures 2E and S10C). Interestingly, MboB cannot be substituted with chemical reductants (ascorbic acid) and/or the use of electron mediators (NADH and phenazine methosulfate) as observed with other HDOs.^20^ Thus, MboAB may be the first characterized example of a dedicated HDO-redox partner pair where interactions with MboB are necessary to enable multiple turnovers of MboA.

### Mangotoxin Structure and Reactivity

We next sought to characterize the structure of mangotoxin by tandem MS/MS and NMR spectroscopy. First, an analysis of the MS/MS fragmentation of mangotoxin shows that the C-terminal arginine is the site of oxidation by MboAB (Figure 3A). The MboABCDE product was then isolated by preparative HPLC and characterized by 1D- and 2D-NMR spectroscopy (Figure S11). By 2D-NMR, we observe ^1^H–^13^C HMBC crosspeaks between the δ protons (3.83 ppm) and two carbons atoms with ^13^C shifts consistent with an alkyne (Figure 3B; 77.97 and 79.90 ppm). Moreover, we observe a long-range ^1^H–^1^H COSY crosspeak between H_δ_ and H_α_ consistent with an unsaturated motif between these sites, as well as the expected absence of protons attached to C_β_ and C_γ_. Based on the MS and NMR data, we conclude that MboA catalyzes the double desaturation of the C-terminal Arg residue to produce an internal alkyne between C_β_ and Cγ (Figure 2A). Finally, using previously established assays, we determined that Leu–Ala–Cit_alkyne_ exhibits antibacterial properties toward *E. coli* (Figure S12), bridging our in vitro results with the previous studies of mangotoxin.^42,43^

**Figure 3:**
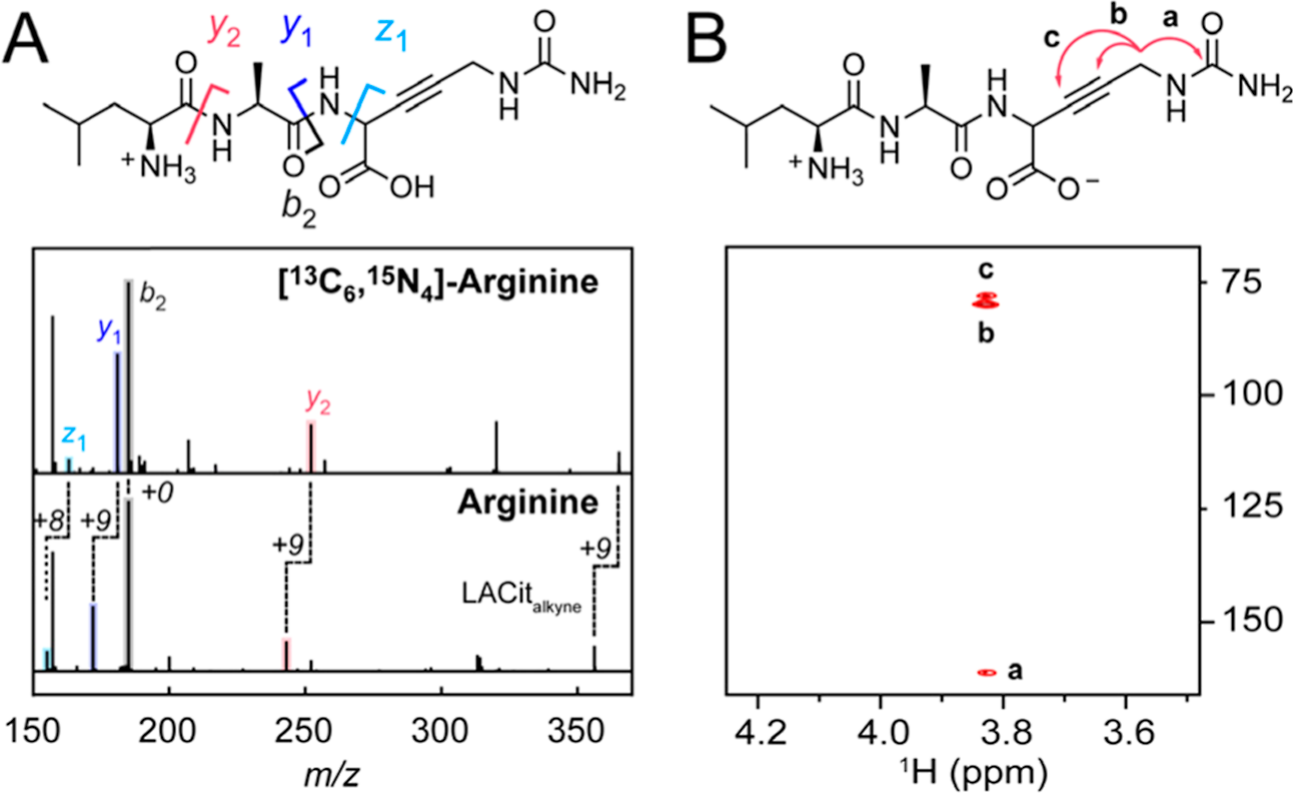
Structural characterization of the product of the mangotoxin BGC made by in vitro reconstitution of MboABCDE. (A) Tandem MS/MS spectrum of Leu–Ala–Cit_alkyne_ derived from a mixture of natural-abundance Arg (bottom) and [^13^C_6_,^15^N_4_]–Arg (top). The fragment ions corresponding to *y*_2_, *y*_1_, and *z*_1_ exhibit a loss of 4H from Leu–Ala–Cit, demonstrating that the modification is localized to the C-terminal residue. List of diagnostic fragment ions for natural abundance Leu–Ala–Cit_alkyne_ (bottom): *m*/*z* [M + H]^+^_obs_ = 356.1950 (356.1928); *m/z y*_2,obs_ = 243.1058 (243.1088); *m/z b*_2,obs_ = 185.1120 (185.1285); *m/z y*_1, obs_ = 172.102 (172.0717); *m/z z*_1, obs_ = 155.0207 (155.0452). List of diagnostic fragment ions for isotopically enriched [^13^C_6_,^15^N_3_]–Leu–Ala–Cit: [M + H]_obs_ = 365.2068 (365.2041); *m/z y*_2,obs_ = 252.1147 (252.1200); *m/z b*_2,obs_ = 185.1271 (185.1285); *m/z y*_1,obs_ = 181.0809 (181.0829); *m/z z*_1,obs_ = 163.0618 (163.0593) (*m*/*z*_calc_ in parentheses). (B) Key ^1^H–^13^C correlations from the HMBC spectrum of the MboABCDE product supporting the presence of an internal alkyne.

A minor peak also appears with the same exact mass in the chromatographic traces of the Leu–Ala–Arg_alkyne_ and Leu–Ala–Cit_alkyne_ tripeptides (Figure 2B). Given the structure and possible reactivity of the internal alkyne at this position, we hypothesized that this peak could represent an isomer that is produced under the acidic quench conditions (Figure S13). When MboAB and MboABE assays were carried out in D_2_O, we observed a mass shift consistent with a H/D exchange at a single position (Figure S13). The isomer was then produced on larger scale from an MboABE reaction with LAR and then subsequently isolated and characterized by NMR (Figures 4 and S14). The ^1^H and ^1^H–^13^C HSQC NMR spectra both exhibited 18 nonsolvent exchangeable protons, rather than the expected 17 protons present in Leu–Ala–Cit_alkyne._ Moreover, we observed two new ^1^H and ^13^C peaks that were shifted unusually downfield (5.22, 81.77 ppm and 7.37 and 130.88 ppm, Figures 4B,C,D and S14C). These observations are consistent with the occurrence of an intramolecular cyclization, as well as the presence of an alkene. Based on the observed chemical shifts, we hypothesize that the C-terminal carboxylic acid cyclizes onto the side-chain of the Cit (and Arg) residue to furnish a lactone with migration of the internal alkyne to form a double bond between C_α_ and C_β_ (Figures 4A and S14A).

**Figure 4:**
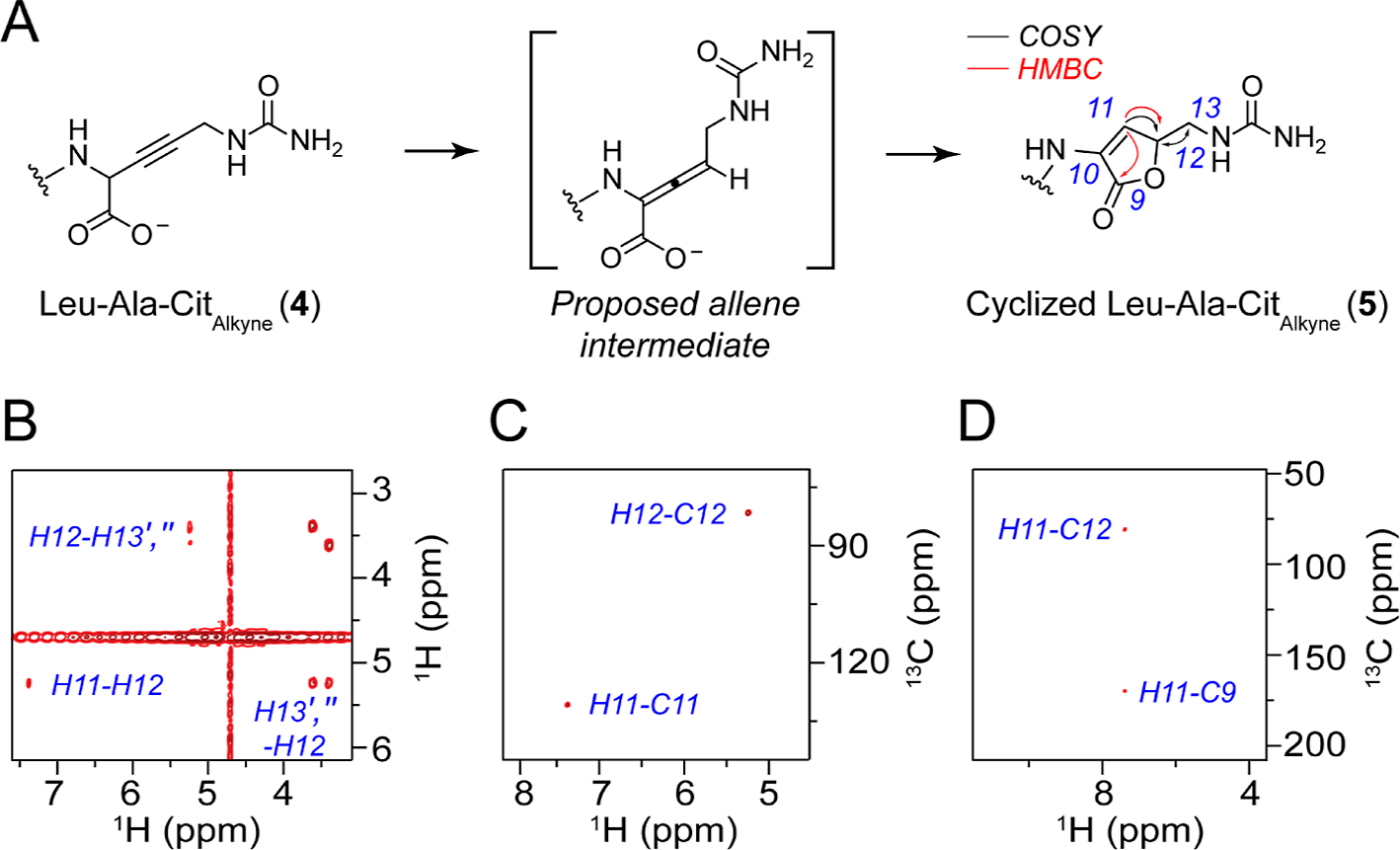
Characterization of the cyclized Leu–Ala–Cit_alkyne_. Leu–Ala–Cit_alkyne_ (**4**) was prepared from the in vitro reconstitution of MboABE with Leu–Ala–Arg (1 mM), NADH (5 mM), Fe­(II) (100 μM), and ascorbate (1 mM). The reaction was quenched in 1% (*v/v*) formic acid in MeCN and incubated overnight before purification by HPLC to isolate the cyclized product. (A) The structure of the cyclized Leu–Ala–Cit_alkyne_ (**5**) is proposed based on the 1D- and 2D-NMR analysis and is hypothesized to form after isomerization of **4** to an allene intermediate (^1^H–^1^H COSY, black; ^1^H–^13^C HMBC, red; Figures S13 and S14). (B) Key ^1^H–^1^H COSY correlations establish the downfield resonances at 5.22 (H12) and 7.37 (H11) ppm derived from the modified peptide as they exhibit cross peaks with the H_δ_ methylene protons and are consistent with lactone formation. Please note that the diagonal cross peaks were suppressed. (C) The ^13^C chemical shifts (C11, 130.88 ppm; C12, 81.77 ppm) from the ^1^H–^13^C HSQC correlations of **5** supports the presence of a lactone and alkene and is consistent with additional NMR analysis (Figure S14). (D) The highlighted ^1^H–^13^C HMBC cross peaks also support the connectivity of **5** and that H11 resides within the lactone ring.

The structure and reactivity of mangotoxin show that the alkyne moiety can act as an electrophile and perhaps provides insight into its biological function as a toxin. Moreover, the observed reactivity is also consistent with initial studies of mangotoxin that demonstrated that it was inactivated over the course of its purification.^42^

### Crystallographic Studies of MboA

To obtain insight into the structure and mechanism of MboA, MboA was crystallized for X-ray crystallographic studies and its structure solved by molecular replacement using its AlphaFold model.^51^ We determined three different crystal structures of MboA: apo MboA (PDB ID 9YPI, 2.49 Å), apo MboA bound to the LAR substrate (PDB ID 9YPL, 2.20 Å), and Fe­(II)_2_ MboA bound to substrate (PDB ID 9YPM, 2.45 Å) (Tables S2–S4, Figure S15). The overall architecture of MboA resembles that of other HDOs, where it exhibits the characteristic seven α-helix bundle (Figures 5A and S16) and supports its assignment as a member of the HDO superfamily.^20^

**Figure 5:**
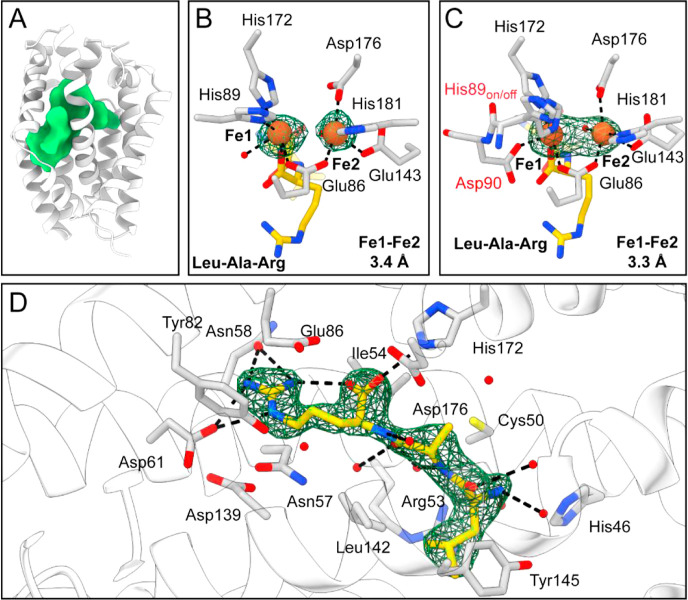
Structural characterization of MboA by X-ray crystallography. (A) Overview of the apo MboA structure (PDB ID: 9YPI). The green surface highlights the MboA central cavity in which the metallocofactor and substrate are housed. Cavity was calculated using the KVFinder^77^ within ChimeraX using the default parameters. (B) Fe­(II)_2_ metallocofactor in Chain B of Fe­(II)_2_-MboA bound (prepared anaerobically) to the LAR tripeptide substrate (Fe occupancy: Fe1, 1.0; Fe2, 1.0). In Chain B, His89 is found coordinated to Fe1 along with the C-terminus of the LAR substrate. Chains C and D are found in a similar coordination geometry. (C) Fe­(II)_2_ metallocofactor in Chain A of Fe­(II)_2_-MboA (prepared anaerobically) bound to the LAR tripeptide substrate (Fe occupancy: Fe1, 1.0; Fe2, 0.85). In Chain A, Asp90 flips toward the metallocofactor, replaces a water molecule, and coordinates to Fe1, whereas His89 is found as an approximately 1:1 mixture of on and off conformations. The C-terminus of the LAR substrate also provides an additional ligand to Fe1 as in Chains BCD. (D) Interactions of the LAR tripeptide substrate with the MboA binding cleft (PDB ID: 9YPL). For clarity, the LAR-bound MboA structure is shown as the LAR conformation does not change dramatically when the Fe­(II)_2_ metallocofactor is present (RMSD: 1.01 Å). Residues shown are within 4 Å of the peptide. Black dashed lines denote potential hydrogen bonds between the substrate and residues and waters within the active site. The composite omit Fo-Fc maps for the Fe atoms are contoured to 6 standard deviations above the mean (σ). The composite omit Fo-Fc map for the peptide is contoured to 2σ.

Comparison of the three different MboA states shows that the structures are quite similar overall (9YPI vs: 9YPL, 0.839 Å r.m.s.d.; 9YPM, 0.853 Å r.m.s.d.), with only small changes, such as the movement of the residues and loops near Fe-coordinating ligands His89 and Glu143 (Figure S17). Upon additional analysis, we observe the presence of a small channel that connects the surface of MboA to the active site that could be used for O_2_ or cofactor assembly (Figure S18A) and a large acidic region near the active site that may recruit MboB, which possesses a complementary basic patch (Figure S18B).

We next examined the coordination environment of the Fe­(II)_2_ cofactor of MboA. Three out of the four monomer units of the MboA tetramer exhibit the canonical 3-His (His89, 172, and 181), 3-Glu/Asp (Glu86, Glu143, and Asp176) coordination to the Fe­(II)_2_ active site found in most other HDOs (Chains BCD, Figures 5B and S19). However, Chain A interestingly features an additional ligand, Asp90, bound to Fe1, with His89 bound to Fe1 at partial occupancy (Figures 5C and S19B). Sequence analysis of MboA-like HDOs shows that Asp or Glu at this position (Asp90) appears to be at least partially conserved (Figure S19C,D), suggesting that ligand exchange at this site could be functionally relevant. Finally, in addition to the protein-derived ligands, the MboA substrate provides an additional ligand—via the C-terminal carboxylate—to the Fe1 site like AetD,^25,26^ UndA,^22^ HrmI,^27^ and PolF,^28,29^ (Figure 5B,C). This observation suggests that substrate coordination may play a role in cofactor assembly and/or triggering O_2_ activation as observed in other HDOs.^20^

In addition to coordinating to the Fe1 site, the Leu–Ala–Arg (LAR) tripeptide is also recognized via H-bonds with Asn57, Asp61, and Asp139, which stabilize the guanidinium group and likely confer selectivity over Leu–Ala–Cit, as well as other residues that either orient waters for H-bonding or provide hydrophobic contacts to the rest of the peptide (Figure 5D). These factors contribute to positioning substrate such that the site of modification extends toward the Fe2 site. This contrasts with other HDOs that coordinate substrate via Fe1 and position the substrate such that it extends away from the cofactor (Figure S20). As a result, the β-carbon of the Arg side-chain is placed within ∼ 3.5 Å of the Fe2-bound water, within the distance expected for activation of the C_β_–H bonds by H atom abstraction (Figures 6 and S21). Notably, the γ-carbon is placed 3.1 Å from the Tyr82 phenol, within appropriate distance for this residue to act as a base for desaturation (Figure 6).^52,53^ As such, the crystal structure of MboA is consistent with alkyne formation occurring between C_β_ and C_γ_ of the C-terminal arginine.

**Figure 6:**
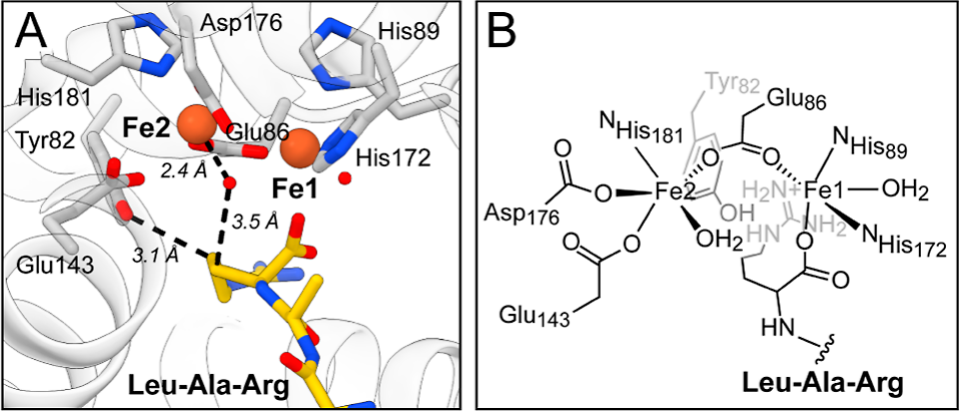
MboA active site geometry. (A) Close-up view of the Fe­(II)_2_ MboA active site with LAR bound (Chain B). The orientation of the Arg residue places C_β_ within distance for hydrogen atom abstraction by an unknown high-valent intermediate based on the distance between C_β_ and the water bound to Fe2 (C_β_–H_2_O_Fe2_ = 3.5 Å). The phenol moiety of Tyr82 is found within distance to act as a base for deprotonation of C_γ_ (C_γ_–O_Tyr82_ = 3.1 Å). The Fe2–H_2_O and Fe1–H_2_O distances are 2.4 Å and 2.9 Å, respectively, consistent with the Fe2-bound water as a terminal ligand, rather than bridging the two Fe sites. (B) Schematic depiction of the MboA active site.

However, the most notable structural feature of MboA is related to the geometry of the diiron site. In all four chains, the Fe–Fe distance is drastically shorter (3.3–3.4 Å) than observed in other Fe­(II)_2_ HDO structures to date (4.4–5.0 Å) (Figures 5B,C and S22).^22,23,25–29^ The relatively long Fe–Fe distances observed in HDOs, compared to ferritin-like dioxygenases (FDOs) like the soluble methane monooxygenase (sMMO, <4 Å), raise the question of what species are involved in HDO catalysis.^20^ These extended Fe–Fe distances may preclude access to the higher-valent intermediates that result from O–O bond cleavage, as observed in many FDOs.^20,54^ Interestingly, the short Fe–Fe distance found in MboA appears to correlate with the geometry of the μ-1,3-bridging Glu86 ligand, which exhibits an acute ∠Fe–O–O angle (104°), more reminiscent of metalloenzymes like sMMO (102°) (Figure S22A).^54^ While most HDOs characterized to date do not feature a bridging ligand, SznF and AetD do contain a similar bridging Glu. However, these enzymes exhibit a significantly more obtuse ∠Fe–O–O angle (SznF,^23^ 147°; AetD,^25,26^ 165°; Figure S22A), which leads to relatively long Fe–Fe distances. Thus, the Fe–Fe distance of HDOs may govern what O_2_ activation pathways are accessible and dictate, in part, the reactivity of different active sites (Figure S22D).

## Discussion

Alkyne-containing compounds represent key synthetic intermediates for a range of downstream chemical transformations.^55–57^ Despite the growing interest in using biocatalysis to prepare alkyne-containing molecules, there remain few enzymatic systems available for this purpose.^58–63^ While there are a large number of acetylenic natural products (∼2000), most seem to be derived from a fatty acid or polyketide origin.^64–66^ Thus, mangotoxin is unique in that the triple bond is installed directly on the side-chain of a peptide substrate. While the exact function of the alkyne is not known, our studies point to it acting as an electrophilic warhead to inhibit ArgJ, the proposed target of mangotoxin.^42^ While internal alkynes are usually not as reactive as their terminal alkyne counterparts, the cyclization of mangotoxin suggests that it exhibits similar reactivity to previously studied β,γ-ynethioates,^67^ which act as covalent inhibitors through isomerization of the alkyne to the corresponding conjugated allene (Figure 4C).

The chemistry of MboA also expands the known reaction scope of HDOs, as other HDO desaturases terminate at alkene formation. While UndA^68^ and BesC^11^ both couple desaturation to loss of CO_2_ and NH_3_, respectively, MboA and VarO^69^ catalyze the more difficult oxidation of their substrates through the formal loss of H_2_. Interestingly, MboA and VarO (∼30% sequence identity) utilize similar substrates, but the presence of a dedicated redox partner, MboB, in the mangotoxin BGC enables a second turnover of MboA to form the alkyne through an unknown mechanism. Moreover, alkyne formation is energetically demanding as it requires the activation of strong C­(sp^2^)-H bonds (BDE ∼ 107 kcal/mol)^70^ while also avoiding more thermodynamically accessible outcomes such as alkene epoxidation or activation of weaker C–H bonds.^53,71^ Thus, it is possible that the unexpectedly short Fe–Fe distance observed in the Fe­(II)_2_-MboA structure is also an important feature in the ability of MboA to catalyze this challenging reaction.

Only one other enzyme family is known to catalyze a similar reaction to MboA: the unrelated membrane-bound fatty acid desaturase-like (FADs) acetylenases (Figure S23).^53,58,60,66,72^ However, issues with poor biochemical properties have stood in the way of the structural and mechanistic study of these acetylenases.^53^ As a result, we have little mechanistic insight into alkyne formation and much of our understanding of FAD-like acetylenases comes from the study of related family members (e.g., FADs, AlkB).^72–75^ These enzymes also possess a diiron cofactor, but their metal–metal distances (∼6 Å) are far longer than those of even other HDOs. As a result, the question of how they activate O_2_ is longstanding.^48,74^ It has recently been proposed that the Fe sites in AlkB operate independently from one another, with one site responsible for O_2_-activation, while the other is responsible for shuttling electrons and protons.^75^

The crystal structure of MboA presents a first look into the structure of a metal-dependent acetylenase, revealing an active site that is structurally distinct from other HDOs and other diiron enzyme families that form alkynes. The short Fe–Fe distance observed in MboA may hint that alkyne formation utilizes a different pathway for O_2_ activation, and perhaps resembles the chemistry of FDOs, where the Fe–Fe distance is short enough to accommodate bridging hydroxy- and oxo-containing intermediates (Figure S22D).^54^ Indeed, the well-studied FDO, sMMO, supports a similar active site (Figure S22A) and can functionalize CH_4_, whose C–H bond strengths (105 kcal/mol) are similar to that of vinyl C–H bonds.^76^

## Supplementary Material







## Data Availability

The data supporting the findings of this study are available within the paper and its Supporting Information files. Should any raw data files be needed in another format they are available from the corresponding author upon request. Crystallographic data for structures have been deposited in the Protein Data Bank (PDB IDs: 9YPI, 9YPL, and 9YPM).

## Notes

Experimental details; characterization data; and further discussion of the experimental data (PDF)
The list of putative HDO sequences based on our structural analysis with their associated ID (TXT)
The script used to identify them is provided (TXT)
