# Supplementary material for: Discovery of a Structurally Distinct Acetylenase in the Biosynthesis of Mangotoxin
Source: J Am Chem Soc. 2026 Apr 28;148(18):18724–32. doi: 10.1021/jacs.5c21680 (PMC13185094; doi:10.1021/jacs.5c21680)
Supplement: Supplementary file 1 [file ja5c21680_si_001.pdf]

Supplementary Information for

Discovery of a structurally distinct acetylenase in the biosynthesis of mangotoxin

Edward D. Badding, Elijah N. Kissman, Stefan V. Velculescu, and Michelle C.Y. Chang*

Materials and Methods

Commercial materials S3
Bacterial strains S3
Bioinformatic analysis of the HDO superfamily S3
Construction of plasmids and E. coli strains used in this study S5
Expression of His-tagged MboACDE S6
Expression of His-tagged MboB S6
Purification of His-tagged MboACDE S6
Purification of His-tagged MboA for protein crystallography S7
Purification of His-tagged MboB S7
In vitro screen of MboC, MboD, and MboE S7
In vitro screen of MboAB S7
In vitro reconstitution of MboABE with Leu-Ala-Arg S7
In vitro reconstitution of MboABCDE S8
LC/QTOF-MS analysis of di- and tripeptides by reverse-phase chromatography S8
LC/QTOF-MS analysis of in vitro Mbo assays by hydrophilic interaction chromatography (HILIC) S8
Steady-state kinetic characterization of dipeptide formation with MboC and MboD S9
Steady-state kinetic characterization of MboD with respect to Leu, Ile, and Met S9
Synthesis of Ala-Arg S9
Rapid HILIC LC/QTOF-MS analysis of column fractions S10
General methods for NMR characterization of reported compounds S10
In vitro production and purification of mangotoxin (Leu-Ala-Cit$_{alkyne}$) S10
Assessing the bioactivity of Leu-Ala-Cit and Leu-Ala-Cit$_{alkyne}$ S11
In vitro MboABE assays in H_2O and D_2O to monitor alkyne tripeptide cyclization S11
In vitro production and purification of cyclized Leu-Ala-Cit$_{alkyne}$ S11
Crystallization of apo MboA and data collection S12
Crystallization of LAR-bound apo MboA and data collection S12
Crystallization of LAR-bound Fe(II)$_2$-MboA and data collection S12
Structure determination S13
Docking a binuclear metallocofactor into an HDO AlphaFold model S13

Figures and Tables

Table S1. *Strains, plasmids, oligonucleotides, gene sequences, and amino acid sequences.* S14
Figure S1. *Bioinformatic workflow to identify HDO sequences by structural analysis* S19
Figure S2. *Bioinformatic analysis of IPR016084 members with a predicted bimetallic site* S20
Figure S3. *Representative SDS-PAGE gel of purified Mbo proteins* S25
Figure S4. *Analysis of in vitro MboC, MboD, and MboCD screening assays* S26

Figure S5. *Characterization of the product of in vitro MboCDE reconstitution* S27
Figure S6. *Steady-state kinetic characterization of dipeptide formation by MboC and MboD* S28
Figure S7. *Steady-state kinetic characterization of tripeptide formation by MboD* S29
Figure S8. *1D- and 2D-NMR characterization of Ala-Arg substrate* S30
Figure S9. *Comparison of MboB to a related reductase (GcoB)* S34
Figure S10. *Characterization of in vitro MboABCDE reconstitution* S35
Figure S11. *1D- and 2D-NMR characterization of Leu-Ala-Cit$_{alkyne}$* S36
Figure S12. *E. coli* growth inhibition assays with Leu-Ala-Cit and Leu-Ala-Cit$_{alkyne}$ S42
Figure S13. *LC-MS/MS characterization of Leu-Ala-Cit$_{alkyne}$ cyclization in H_2O and D_2O* S43
Figure S14. *1D- and 2D-NMR characterization of cyclized Leu-Ala-Cit$_{alkyne}$* S44
Table S2. *Data collection and refinement parameters for apo MboA from Pseudomonas syringae* S52
Table S3. *Data collection and refinement parameters for MboA bound to LAR* S53
Table S4. *Data collection and refinement parameters for $Fe(II)_2$ MboA bound to LAR* S54
Figure S15. *Electron density maps of Chain A and Chain B of $Fe(II)_2$ MboA bound to LAR* S55
Figure S16. *Comparison of MboA to other HDOs* S56
Figure S17. *Comparison of the three MboA structures* S57
Figure S18. *Channel and electrostatic analysis of MboA* S58
Figure S19. *Sequence and structural analysis of the MboA coordination environment* S59
Figure S20. *Comparison of substrate positioning in HDOs* S61
Figure S21. *MboA active site geometry with the substrate C–H bonds modeled* S62
Figure S22. *Analysis of the metal-metal distances in MboA and other binuclear metalloenzymes* S63
Figure S23. *Comparison of MboA with HDOs, FADs, FAD-like enzymes, and sMMO* S64

References S66

Materials and Methods

Commercial materials. Luria-Bertani (LB) Broth Miller, LB Agar Miller, and glycerol were purchased from EMD Biosciences (Darmstadt, Germany). Carbenicillin (Cb), isopropyl-β-D-thiogalactopyranoside (IPTG), sodium chloride, dithiothreitol (DTT), 4-(2-hydroxyethyl)-1-piperazineethanesulfonic acid (HEPES), potassium chloride, magnesium chloride hexahydrate, acetonitrile, and Nanosep 10K Omega filters were purchased from Fisher Scientific (Pittsburgh, PA). Adenosine triphosphate sodium salt (ATP), flavin adenine dinucleotide disodium salt (FAD), ammonium iron (II) sulfate, β-nicotinamide adenine dinucleotide reduced form disodium salt (NADH), lysozyme, DNAse from bovine pancrease lyophilized powder, magnesium sulfate heptahydrate, riboflavin 5′-monophosphate disodium salt, β-mercaptoethanol (βME), dimethylsulfoxide (DMSO), acetonitrile (LC/MS-grade), ammonium formate (LC/MS-grade), sodium ascorbate, all 20 proteinogenic amino acids (L-alanine, L-arginine, L-asparagine, L-aspartic acid, L-cysteine (HCl), L-glutamic acid (potassium salt), L-glutamine, L-glycine, L-histidine, L-isoleucine, L-leucine, L-lysine, L-methionine, L-phenylalanine, L-proline, L-serine, L-serine, L-threonine, L-tryptophan, L-tyrosine, L-valine), and L-ornithine were purchased from Sigma-Aldrich (St. Louis, MO). PageRuler Plus Prestained Protein Ladder, N-Fmoc-L-alanine (95%), sodium acetate anhydrous and tris(hydroxymethyl)aminomethane were purchased from Thermo Fisher Scientific (Waltham, MA). Bacto™ Agar was purchased from BD (Sparks, Maryland). Formic acid was purchased from Acros Organics (Morris Plains, NJ). Phusion DNA polymerase was purchased from New England Biolabs (Ipswich, MA). Deoxynucleotides (dNTPs), were purchased from Invitrogen (Carlsbad, CA). Oligonucleotides were purchased from Integrated DNA Technologies (Coralville, IA), resuspended at a stock concentration of 100 μM in 10 mM Tris-HCl, pH 8.5, and stored at 4 °C. DNA purification kits and Co/Ni-NTA agarose were purchased from Qiagen (Valencia, CA). Complete EDTA-free protease inhibitor was purchased from Roche Applied Science (Penzberg, Germany). PD-10 desalting columns were purchased from GE Healthcare Life, (Pittsburg, PA). Amicon Ultra 10,000 MWCO and 30,000 MWCO centrifugal concentrators were purchased from Millipore (Billerica, MA). 8-16% Mini-PROTEAN TGX precast protein gels were purchased from Bio-Rad (Philadelphia, PA). PyBOP and ammonium dihydrogen orthophosphate were purchased from Oakwood chemicals (Esstill, SC). Fmoc-Arg(Pbf) loaded Wang resin (100-200 mesh, 0.2-0.8 meq per g) was purchased from Chem-Impex (Wood Dale, IL). The peptides Leu-Ala-Arg and Ile-Ala-Arg were purchased as the acetate salt from Biomatik (Ontario, Canada). Lithium sulfate was purchased from Arcos Organics (Geel, Belgium). Glacial acetic acid was purchased from Rowley Biochemicals (Danvers, MA). Isotopically-labelled amino acids and D_2O were purchased from Cambridge Isotope Laboratories (Andover, MA). The bovine serum albumin was purchased as a 1 mg/mL standard from Abcam (Cambridge, UK). All chemicals were used as purchased without purification.

Bacterial strains. *Escherichia coli* DH10B-T1^R was used for plasmid construction (*Table S1*). *E. coli* BL21 Star (DE3) was used for heterologous protein production.

Bioinformatic analysis of the HDO superfamily. A key challenge in assigning a sequence as a heme oxygenase-like domain containing oxidase (HDO) is identifying the presence of the Fe_2 metallocofactor ligands. Typically, these ligands can be identified in sequence space through sequence alignments. For example, radical *S*-adenosyl methionine (SAM) enzymes utilize the characteristic CXXXCXXC motif to bind their Fe_4S_4 cluster [*1*]. Utilizing this strategy for HDOs is challenging owing to the presence of loop insertions (**Fig. S16**) that interrupt these sequence motifs [*2, 3*]. To overcome this challenge, we took inspiration from previous studies on HDOs and

other metalloenzymes [*3-8*] and sought to enrich for putative HDO sequences by utilizing both sequence and structural (using AlphaFold models [*9*]) information.

To obtain a list of putative of Fe_2-binding HDOs, we first acquired all sequence IDs from the InterPro family IPR016084 (**Fig. S1**, *step 1*; 76,090, 06/2025 and 11/2025, duplicate sequences were removed) and InterPro family IPR045776 (31, 01/2026) for a total of 76,121 sequences. The IPR016084 family encompasses multi-helical proteins with a broad range of annotations, such as coenzyme pyrroloquinoline-quinone synthase (PqqC), prokaryotic haem oxygenases, TENA/Thi4 proteins, and HDOs [*2*]. As such, only a subset of IPR016084 members are HDOs, defined by the spatial arrangement of the metal-binding residues used to coordinate the binuclear metal active site [*2*]. The IPR045776 family encompasses PolF and PolF-like sequences [*10, 11*] and were added to our list of sequences as internal controls for our study.

We next performed a structural and sequence pairwise analysis in which we individually aligned each candidate sequence and/or model to that of a characterized HDO (SznF, UndA, CADD, FlcE, AetD, PolF and BesC) [*2, 10, 11*]. Note that of the ~76,000 sequences acquired, only 63,711 had their corresponding AlphaFold model available (**Fig. S1**, *step 2*; 01/2026). As a result, the remaining sequences were only subjected to sequence alignments, which will be discussed further below.

Our workflow for identifying HDOs based on a pairwise structural alignment involved aligning each target AlphaFold model to seven reference AlphaFold models (SznF, UndA, CADD, FlcE, AetD, PolF and BesC) using the 'super' function in PyMol (**Fig. S1**, *step 3*). Once the target and reference model were in the same 3D frame of reference, we searched for all the residues present (location defined by the coordinates of their C_α) within 5 Å of the reference metal binding residue (location defined by the coordinates of their C_α) (**Fig. S1**, *step 4*). The identity and distance of all target model residues that were within 5 Å of the reference metal binding residues were then recorded and stored in a .tsv file. From the tabulated data, two pieces of information were extracted: (1) the average number of identified metal binding residues (Asp/Glu/His) over all target and reference pairs and (2) the average distance between the closest identified Asp, Glu, or His and the reference metal binding residue for all target and reference pairs. Note that for these calculations, we made sure that each matched residue is unique; *i.e.* if the same target residue is identified for multiple references, the closest one is used and the next closest Asp/Glu/His would be matched for the next reference/target pair, if present (**Fig. S1**, *step 5*). Each sequence was then binned based on a 4×4 matrix presented below (see below). Sequences that matched the constraints highlighted in green (Tier 1 + 2) were considered putative HDOs (**Fig. S1**, *step 6*). From this analysis, 20,866 unique sequences were predicted to be an Fe_2-dependent HDO.

Avg. number of putative metal binding ligands	Avg. deviation from reference residues (Å): 0-2	2-3	3-4	4+
**0-2**	Tier 4	Tier 5	Tier 6	Tier 6
**2-3**	Tier 3	Tier 4	Tier 5	Tier 6
**3-4**	Tier 2	Tier 3	Tier 4	Tier 5
**4+**	Tier 1	Tier 2	Tier 3	Tier 4

As a complementary study to the analysis presented above, we also used pairwise sequence alignments to provide (1) some information on the remaining sequences that do not have a corresponding AlphaFold model; and (2) complementary information to the structural analysis.

Using the same references discussed above, we performed a pairwise sequence analysis with each target. A sequence was designated as an HDO if at least five of the six metal-binding residues were aligned with an Asp, Glu, or His in the target sequence relative to the reference. All sequence alignments were carried out using ClustalO (23,621 sequences identified) [*12*]. This strategy likely introduces a number of false positives, which can be verified either by (1) cross-referencing the information with the structural analysis or (2) submitting the sequence to the AlphaFold server and manually analyzing the corresponding AlphaFold model to verify if the sequence has the appropriate ligands in the proper locations to bind an Fe_2 metallocofactor. A .tsv file with each target sequence was then generated with their corresponding grade from the structural analysis, and whether or not their sequence was flagged as a putative HDO based on the pairwise sequence alignment.

The data was visualized by generating an SSN of the full IPR016084 family (alignment score of 42, which corresponds to a sequence identity of approximately 35%) using the EFI-EST tool [*13*] and visualized using Cytoscape [*14*] (**Fig. S2A**). Following the sequence cutoffs (min = 130 and max = 600 amino acids) and redundancy filters by the EFI server, 64,061 sequences remained. We elected to have (1) the highlighted nodes correspond to the structural information; and (2) the shape of each node correspond to the sequence information. Note that a supplementary .tsv file is provided that contains all the information from the structure and sequence analysis. The SSN presented is a representative node network set to 50%.

A second SSN was generated using the sequences identified as putative HDOs [*13*] from our structural analysis (**Fig. S2B**). This network was constructed using a sequence alignment score of 42 (corresponding to a sequence identity of approximately 40%). Following the sequence cutoffs (min = 130 and max = 600 amino acids) and redundancy filters by the EFI server, 19,778 sequences remained. This SSN was then submitted to the EFI-GNT tool to obtain the genomic information surrounding each putative HDO. Neighborhood distance and co-occurrence cutoffs were set to 10 and 20, respectively. The second SSN presented is a representative node network set to 65%.

Using Cytoscape [*14*], we attempted to identify HDO sequences (824 out of 8754 representative nodes, 1,447 sequences) that colocalize with at least one ATP-grasp using the following InterPro families: 013651, 011761, 053705, 003806, 013815, 00315, 040570, and 052032. From this analysis, we observed several clusters of nodes that contained biosynthetic gene clusters (BGCs) our desired BGC architecture (**Fig. S2BCD**), including the BGCs that contain SznF [*4*] and BesC [*15*]—these serve as positive controls for our workflow.

From this analysis, we identified at least ~25 unique biosynthetic gene clusters that match these criteria. While HDOs also colocalize with other biosynthetic machinery, such as nonribosomal peptide synthetases (NRPSs), polyketide synthases (PKSs), ribosomally synthesized and posttranstionally modified peptide (RiPP) enzymes (1970 sequences for PKS/NRPS-containing clusters; InterPro families: 016039, 050091, 014031, 020841, 012223, and 010071) [*2*], we elected to focus on ATP-grasp containing BGCs because we find that these provide standalone enzymes that are useful for biocatalytic applications.

Construction of plasmids and *E. coli* strains used in this study. Gibson assembly [*16*] was used to carry out plasmid construction using *E. coli* DH10B-$T1^R$ as the cloning host. *E. coli* codon-optimized GeneBlocks were purchased from Twist Bioscience (San Francisco, CA). All PCR amplifications were carried out with Phusion polymerase. Oligonucleotides used are listed in *Table S1*. Following plasmid construction, all cloned inserts were sequenced using Plasmidsaurus (San Francisco, CA).

pET16b-His_{10} (N-term) was used for as the plasmid backbone for protein expression. Vectors for protein expression were constructed by (1) PCR amplification of the pET16b backbone (*Table S1B*) followed by (2) a two-piece Gibson assembly with the synthetic gene block and PCR-amplified pET16b backbone. Following assembly, the Gibson reaction was added to a 100 µL aliquot of chemically-competent *E. coli* along with 20 mL of KCM buffer (500 mM KCl, 150 mM $CaCl_2$, 250 mM $MgCl_2$), and 160 µL MilliQ H_2O. The cells were incubated on ice for 30 min, followed by heat shock at 42 °C for 90 s. The cells were plated after a 1 h recovery incubation in LB at 37 °C.

Expression of His-tagged MboACDE. Single colonies of *E. coli* BL21 Star (DE3) transformed with the appropriate expression plasmid were inoculated into LB (5 mL) containing 50 µg/mL carbenicillin (Cb) and grown overnight at 37°C with shaking at 200 rpm. The overnight culture (1 mL) was then inoculated into 1 L of LB Cb in a 2.8 L-baffled shake flask. Cultures were incubated at 37 °C and 200 rpm until the OD_{600} reached 0.4 - 0.6 at which point they were cooled on ice for 20 min. Protein expression was induced with IPTG (0.1 mM) and then grown for an additional 18 h at 16°C and 200 rpm. Cell pellets were harvested by centrifugation at 8,000 × g for 7 min at 4ºC and stored at -80ºC.

Expression of His-tagged MboB. Single colonies of *E. coli* BL21 Star (DE3) transformed with the appropriate expression plasmid were inoculated into LB (5 mL) containing 50 µg/mL Cb and grown overnight at 37°C with shaking at 200 rpm. The overnight culture (1 mL) was then inoculated into 1 L of LB Cb in a 2.8 L-baffled shake flask. Cultures were incubated at 37 °C and 200 rpm until the OD_{600} reached 0.6 - 0.8 at which point they were supplemented with 50 µM each of ammonium iron (II) sulfate, riboflavin, and L-cysteine. Cultures were then cooled on ice for 20 min before inducing protein expression with IPTG (0.05 mM) and growing for an additional 18 h at 16°C and 200 rpm. Cell pellets were harvested by centrifugation at 8,000 × g for 7 min at 4ºC and stored at -80ºC.

Purification of His-tagged MboACDE. Frozen cell pellets from a 1 L growth were thawed and resuspended in 45 ml of lysis buffer (25 mM Tris pH 8.0, 250 mM NaCl, and 20% (*v/v*) glycerol) supplemented with EDTA-free protease inhibitor cocktail (Roche), lysozyme (0.3 mg per mL), and DNAse (100 µg per mL). The cell paste was lysed by sonication while stirring on ice with a Qsonica Sonicator 3000 (power = 35%, 2 s on, 45 s off, 1 min total process time, 0.5-inch tip). The resulting lysate was then clarified through centrifugation at 10,000 × *g* for 60 min at 4 °C. The soluble fraction was then loaded onto a Ni-NTA column (Qiagen) at 6 °C by gravity flow. The column was washed with 5 column volumes of wash buffer (25 mM Tris pH 8.0, 250 mM NaCl, 20 mM imidazole, and 20% (*v/v*) glycerol). The protein was then eluted with elution buffer (25 mM Tris pH 8.0, 250 mM NaCl, 200 mM imidazole, and 5% (*v/v*) glycerol). Fractions containing the target protein were pooled according to absorbance at 280 nm and concentrated using an Amicon Ultra spin concentrator (10 kD MWCO; Millipore). Protein was then exchanged into storage buffer (25 mM Tris pH 8.0, 250 mM sodium chloride, and 5% (*v/v*) glycerol) using PD-10 desalting columns. Final protein concentrations before storage were estimated using the $\varepsilon_{280\ nm}$ calculated by ExPASy ProtParam [*17*] and measured using a Nanodrop spectrometer. They are as follows: His_{10}-MboA: $\varepsilon_{280\ nm}$ = 20,400 $M^{-1}cm^{-1}$, *E. coli* His_{10}-MboC: $\varepsilon_{280\ nm}$ = 27,850 M^{-1} cm^{-1}, His_{10}-MboD: $\varepsilon_{280\ nm}$ = 26,360 M^{-1} cm^{-1}, and His_{10}-MboE: $\varepsilon_{280\ nm}$ = 33,920 M^{-1} cm^{-1}. All proteins were aliquoted, flash frozen in liquid nitrogen and stored at −80 °C. Working concentrations for each protein ranged from 600-1500 µM, 100-400 µM, 300-600 µM, and 100-300 µM, respectively.

Purification of His-tagged MboA for protein crystallography. Following Ni-NTA purification, His_{10}-MboA was further purified via size-exclusion chromatography. The protein was concentrated to >2 mL and loaded onto a HiLoad 16/600 Superdex 200 pg column (GE Healthcare) equilibrated in 250 mM NaCl, 25 mM Tris pH 8.0, and 5% (*v/v*) glycerol. Protein-containing fractions were eluted within 10-15 column volumes of the equilibration buffer. Fractions (1 mL) containing His_{10}-MboA were determined by SDS-PAGE gel electrophoresis, pooled, and concentrated to ~18 mg per mL (~650 μM). The protein was aliquoted, flash frozen in LN_2, and stored at −80 °C.

Purification of His-tagged MboB. Frozen cell pellets from a 2 L growth were thawed and resuspended in 90 ml of lysis buffer (25 mM Tris pH 9.0, 100 mM NaCl, and 20% (*v/v*) glycerol) supplemented with EDTA-free protease inhibitor cocktail (Roche), lysozyme (0.3 mg per mL), DNAse (100 μg per mL), and 100 μM FAD. The cell paste was lysed by sonication while stirring on ice with a Qsonica Sonicator 3000 (power = 35%, 2 s on, 45 s off, 1 min total process time, 0.5-inch tip). The lysate was then clarified by centrifugation at 10,000 × *g* for 60 min at 4 °C. The soluble fraction was then loaded onto a Co-NTA column (Qiagen) at 6 °C by gravity flow. The column was washed with 5 column volumes of wash buffer (25 mM Tris pH 8.0, 100 mM NaCl, 20 mM imidazole, and 20% (*v/v*) glycerol). The protein was then eluted with elution buffer (25 mM Tris pH 9.0, 250 mM NaCl, 200 mM imidazole, and 5% (*v/v*) glycerol). Fractions containing the target protein were pooled according to absorbance at 450 nm and concentrated using an Amicon Ultra spin concentrator (10 kD MWCO; Millipore). Protein was then exchanged into storage buffer (25 mM Tris pH 9.0, 100 mM sodium chloride, and 5% (*v/v*) glycerol) using PD-10 desalting columns. Final protein concentrations before storage were estimated via the Bradford protein assay [*18*] prior to storage. Working concentrations for MboB ranged from 50 to 150 μM.

***In vitro* screen of MboC, MboD, and MboE.** Assays (100 μL) contained a 1 mM amino acid mix (20 proteinogenic amino acids and L-Orn), 5 mM ATP, 5 mM $MgCl_2$, and 1 μM of each enzyme included in the assay (MboC, MboD, MboCD, MboCDE) in 500 mM ammonium formate pH 8.0. [$^{13}C_6$, ^{15}N]-L-Ile is used in place of natural abundance L-Ile to distinguish between L-Leu and L-Ile-containing products. Reactions were incubated at room temperature for at least 1 h before quenching with one volume of 1% (*v/v*) formic acid in MeCN. Samples were centrifuged for 10 min at 21,000 × *g* at room temperature before analysis by LC/QTOF-MS using the reverse-phase method for di- and tri-peptides.

***In vitro* screen of MboAB.** Assays (100 μL) contained a 0.1 mM amino acid mix (20 proteinogenic amino acids and L-Orn), 0.1 mM [$^{13}C_6$, $^{15}N_4$]-L-Arg, 5 mM ATP, 5 mM $MgCl_2$, 2 mM NADH, 500 μM ferrous ammonium sulfate, 1 mM ascorbic acid, and 1 μM each of MboCDE in 500 mM ammonium formate pH 8.0. When included, MboB was added at 1 μM final concentration and MboA as added at 10 μM final concentration. Reactions were incubated at room temperature for at least 1 h before quenching with one volume of 1% (*v/v*) formic acid in MeCN. Samples were centrifuged for 10 min at 21,000 × *g* at room temperature before analysis by LC/QTOF-MS using the reverse-phase method for di- and tri-peptides.

***In vitro* reconstitution of MboABE with Leu-Ala-Arg.** Reactions (100 μL) contained 1 mM 0.25 mM Leu-Ala-Arg, 1 mM ascorbic acid, 100 μM ferrous ammonium sulfate, and 2.5 mM NADH in 500 mM ammonium formate pH 8.0. The final concentrations for MboB and MboE were 1 μM each when included whereas MboA was added to 10 μM final concentration when included. In order to assess the ordering of MboAB vs. MboE, reactions were prepared with either MboAB or MboE included and incubated at room temperature for 2 h. MboE was then added to

the MboAB reaction and MboAB was added to the MboE reaction followed by an additional incubation for 1 h. Reactions were also run to test the contribution of individual components to alkyne formation by systematic omission and incubated at room temperature for 1 h. When used, phenazine was added to a final concentration of 100 µM. Assays were quenched with one volume of 1% (*v/v*) formic acid in MeCN and centrifuged for 10 min at 21,000 × *g* at room temperature before analysis by LC/QTOF-MS using the HILIC method for *in vitro* Mbo assays.

***In vitro* reconstitution of MboABCDE.** Reactions (100 µL) contained 1 mM L-Ala, 1 mM L-Arg, 1 mM L-Leu, 5 mM ATP, 5 mM $MgCl_2$, 1 mM ascorbic acid, 100 µM ferrous ammonium sulfate, 2.5 mM NADH in 500 mM ammonium formate pH 8.0. The final concentrations for MboB, MboC, MboD and MboE were 1 µM each when included whereas MboA was added to 10 µM final concentration when included. For LC-MS/MS analysis of Leu-Ala-Cit and Leu-Ala-Cit_{alkyne}, the MboABCDE reconstitution assay also included labeled L-Arg, as such the assays contained 0.5 mM natural abundance L-Arg and 0.5 mM [$^{13}C_6$, $^{15}N_4$]-L-Arg.

Reactions were incubated at room temperature for at least 1 h before quenching with one volume of 1% (*v/v*) formic acid in MeCN. Samples were centrifuged for 10 min at 21,000 × *g* at room temperature before analysis by LC/QTOF-MS using the HILIC method for *in vitro* Mbo assays.

LC/QTOF-MS analysis of di- and tripeptides by reverse-phase chromatography. Samples were analyzed using an Agilent 1290 UPLC equipped with an ACQUITY Premier Peptide HSS T3, 100 Å column (1.8 µm, 2.1×100 mm; Waters). The mobile phase consisted of buffer A (0.1% *v/v* formic acid in 100% water) and buffer B (100% acetonitrile). After an initial 1 min hold at 100% buffer A, a linear gradient from 100% to 50% buffer A was applied over 8 min, followed by an isocratic wash at 50% buffer A for 1 minute before being re-equilibration at 100% buffer A. The flow rate was set at 0.4 mL/min and the column temperature was maintained at 37.5 ºC. Data were acquired in positive-ionization mode using an Agilent 6530 QTOF-MS with the following source and acquisition settings: Gas temperature = 275 ºC; drying gas = 12 L/min; nebulizer = 25 psi; sheath gas temperature = 325 °C; capillary voltage = 3500 V; fragmentor 180 V; skimmer 65 V; oct 1 RF vpp = 750 V; acquisition rate = 3 spectra/s; acquisition time = 333.3 ms/spectrum; min range = 120 *m/z*; max range = 600 *m/z*.

The data was converted to an .abf file and imported into MSDial [*19*]. The MSDial ion detection and alignment algorithms were used with default settings to identify unique ions, which were compiled into a table. The *m/z*, retention time, and average signal-to-noise ratio was extracted for each ion and transferred to an Excel file. A custom Python script was then used to compared the ion list against the *m/z* values of all potential dipeptides or tripeptides. The signal-to-noise ratio for any ions that matched within error tolerance of Δ35 ppm for dipeptides and Δ20 ppm for tripeptides were extracted into an output file. This output file was used to generate heat maps using a custom MATLAB script.

LC/QTOF-MS analysis of *in vitro* Mbo assays by hydrophilic interaction chromatography (HILIC). Samples were analyzed using an Agilent 1290 UPLC with an ACQUITY Premier BEH Amide column (1.8 µm, 2.1×50 mm; Waters). The mobile phase consisted of buffer A (90% water, 10% acetonitrile and 10 mM ammonium formate, pH 4.5) and buffer B (90% acetonitrile, 10% water and 10 mM ammonium formate pH 4.5). A linear gradient from 0% to 10% buffer A was applied over 1 min, followed by a linear gradient from 10% to 70% buffer A over 6 min. Buffer A was then held at 70% for 1 min before returning the column to 0% buffer A using a linear gradient over 2 min. The flow rate was set to 0.5 mL/min and the column temperature was maintained at

35 ºC. Data were acquired in positive-ionization mode using an Agilent 6530 QTOF-MS. For most samples, data were collected using the following source and acquisition parameters: Gas temperature = 300 ºC; drying gas = 8 L/min; nebulizer = 35 psi; sheath gas temperature = 350 °C; capillary voltage = 3500 V; fragmentor 175 V; skimmer 65 V; oct 1 RF vpp = 750 V; acquisition rate = 1 spectra/s; acquisition time = 1000 ms/spectrum; min range = 80 *m/z*; max range = 800 *m/z*.

For samples that required targeted MS/MS fragmentation data collection, the following source and acquisition parameters were used: Gas temperature = 300 ºC; drying gas = 8 L/min; nebulizer = 35 psig; sheath gas temperature = 350 °C; capillary voltage = 3500 V; fragmentor = 180 V; skimmer 65 V; oct 1 RF vpp = 750 V; acquisition rate = 5 spectra/s; acquisition time = 200 ms/spectrum; min range = 120 *m/z*; max range = 800 *m/z*; collision energy = 15 V; Δ-retention time: 0.02 min; and an of iso. width = 1.3 *m/z*.

Steady-state kinetic characterization of dipeptide formation with MboC and MboD. Steady-state kinetic analysis was performed using a coupled assay that links ATP hydrolysis to NADH consumption. Reactions (100 µL) contained 65 µL of a master mix (100 mM HEPES pH 7.5, 5 mM ATP, 10 mM $MgCl_2$, 2 mM PEP, 5 mM L-Ala, 0.6 mM NADH, and 10 µM pyruvate kinase and lactate dehydrogenase) as well as 1.5 µM MboC or MboD and varying amounts of either L-Arg or L-Cit (2.5, 1.0, 0.5, 0.25, 0.1, 0.05, 0.025, and 0 mM). Reactions were performed in 96-well plates and initiated with addition of the master mix. Initial rates of NADH consumption were measured at A_{340} using a SpectraMax M2 Microplate Reader (Molecular Devices) at room temperature. k_{cat} and K_M were determined by simulating the initial rate data using the following equation with a custom MatLab script:

$$v_o = \frac{k_{cat}[S]}{K_M + [S]}$$

Steady-state kinetic characterization of MboD with respect to Leu, Ile, and Met. Steady-state kinetic analysis was performed using a coupled assay that links ATP hydrolysis to NADH consumption. Reactions (100 µL) contained 93 µL of a master mix (100 mM HEPES pH 7.5, 0.25 mM Ala-Arg, 10 mM $MgCl_2$, 2 mM PEP, 0.6 mM NADH, and 10 µM pyruvate kinase and lactate dehydrogenase) as well as 1.5 µM MboD and varying amounts of either L-Leu, L-Ile, or L-Met (2.0, 1.0, 0.5, 0.2, 0.1, 0.05, and 0 mM). Reactions were performed in 96-well plates and initiated with addition of the master mix. Kinetic traces showed an appreciable amount of background ATP hydrolysis in the no substrate control which was accounted for by subtraction of the raw data for the control from the samples. Data were then fit as described above.

Synthesis of Ala-Arg. Ala-Arg (0.1 mmol) was synthesized manually on L-Arg(Pbf)-Wang resin (0.2 to 0.9 meq per gram). The solid phase synthesis cycle included: (i) Fmoc deprotection with 20% (*v/v*) piperidine in dimethylformamide (DMF) for 20 min and (ii) formation of the peptide by incubating 4 eq of Fmoc-Ala, 4 eq of (benzotriazol-1-yloxy)tripyrrolidinophosphonium hexafluorophosphate (PyBOP) and 8 eq diisopropylethylamine (DIPEA) for 30 min in DMF. The coupling step was repeated twice. Peptide cleavage and side-chain deprotection were carried out by incubating the resin with a cleavage cocktail (95% *v/v* TFA, 2.5% *v/v* TIPS, 2.5% *v/v* H_2O) for 2 h. The cleaved peptide was obtained through vacuum filtration with the flow-through collected in cold diethyl ether to precipitate the product. The mixture was centrifuged at 2,200 × *g* for 10 min at 4 ºC. The peptide was dissolved in 50:50 (*v/v*) acetonitrile:water and purified using an Agilent 1260 II preparative HPLC with a SeQuant ZIC-HILIC column (5 µm, 200 Å, 21.1×50 mm; Sigma-Aldrich) column.

The mobile phase consisted of buffer A (90% water, 10% acetonitrile and 10 mM ammonium formate pH 3.5) and buffer B (90% acetonitrile, 10% water and 10 mM ammonium formate pH 3.5). The column was held at 20% buffer A for 3 min before applying a linear gradient from 20% to 73% buffer A over 7 min followed by another linear gradient from 73% to 80% buffer A over 5 min. The column was then returned to 20% buffer A using a linear gradient over 1 min. The flow rate was set to 15 mL/min. Fractions (4 mL) were collected based on absorbance at 215 nm. Fractions were analyzed using the rapid HILIC LC/QTOF-MS method and fractions containing the product were pooled together and lyophilized to afford a white powder (31 mg, 51% yield).

Rapid HILIC LC/QTOF-MS analysis of column fractions. Fractions from preparative HPLC purifications were screened (1 µL) using an Agilent 1290 UPLC with a SeQuant ZIC-HILIC column (5 µm, 2.1×50 mm; Sigma-Aldrich). The mobile phase consisted of buffer A (90% water, 10% acetonitrile and 10 mM ammonium formate pH 3.5) and buffer B (90% acetonitrile, 10% water and 10 mM ammonium formate pH 3.5). The column was held at 10% buffer A for 0.2 min before applying a linear gradient from 10% to 50% buffer A over 1.3 min. Buffer A was then held at 50% buffer A for 0.75 min before returning the column to 10% buffer A using a linear gradient over 0.75 min. The flow rate was set to 0.5 mL/min, and the column temperature was maintained at 40 ºC. Mass spectra were acquired in positive-ionization mode using an Agilent 6530 QTOF-MS with the following source and acquisition parameters: Gas temperature = 300 ºC; drying gas = 8 L/min; nebulizer = 35 psig; sheath gas temperature = 350 °C; capillary voltage = 3500 V; fragmentor 175 V; skimmer 65 V; oct 1 RF vpp = 750 V; acquisition rate = 1 spectra/s; acquisition time = 1000 ms/spectrum; min range = 80 *m/z*; max range = 400 *m/z*.

General methods for NMR characterization of reported compounds. NMR spectra (^{1}H, ^{13}C, HMBC, and HSQC) were collected at the Princeton University Department of Chemistry NMR facility. Spectra were acquired at 298K on a Bruker Avance NMR 500 MHz instrument using Bruker TopSpin and equipped with a He-cooled cryoprobe equipped with a tunable multinuclear probe or a ^{13}C-detection optimized C-H Dual probe. Data were processed and analyzed in MestReNova using automatic phasing and Bernstein Polynomial Fit baseline correction capabilities. Splitting was determined using the automatic multiplet analysis function and checked manually. The ^{1}H and ^{13}C chemical shifts are internally referenced to H_2O and formate, respectively.

***In vitro* production and purification of mangotoxin (Leu-Ala-Cit$_{alkyne}$).** The reaction mixture (10 mL) contained 1 mM Leu-Ala-Arg, 5 mM NADH, 100 µM ferrous ammonium sulfate, 1 mM ascorbic acid in 500 mM ammonium formate pH 8.0. The reaction was initiated by addition of 10 µM MboA and 1 µM MboB. After incubation at room temperature for 2 h, 5 µM MboE was added and the reaction was incubated for an additional 2 h. The reaction was then lyophilized and resuspended in ddH_2O to a final volume of 2 mL. The crude mixture was centrifuged for 10 min at 21,000 × *g* at room temperature and any remaining soluble protein was removed using a Nanosep 10 kD MWCO centrifuge filter (Pall). Formic acid was added to the flow through to reach a final concentration of 1% (*v/v*) formic acid. Small particulates were removed using a hydrophobic PTFE syringe filter (13 mm, 0.45 µM; Avantor). The product was isolated from the mixture using an Agilent 1260 II preparative HPLC with a XBridge BEH Prep OBD Amide column (5 µm, 18×150 mm; Waters). The mobile phase consisted of buffer A (90% water, 10% acetonitrile and 10 mM ammonium formate pH 4.0) and buffer B (90% acetonitrile, 10% water and 10 mM ammonium formate pH 4.0) and was held for 5 min at 10% buffer A before applying a linear gradient from 10% to 30% buffer A over 9 min. Buffer A was then increased linearly to 70% over 1 min and

held constant for 5 min before returning the column to 10% buffer A using a linear gradient over 1 min. The flow rate was set to 12.5 mL/min at room temperature. Fractions (5 mL) were collected based on absorbance at 215 nm. Fractions were analyzed using the rapid HILIC LC/QTOF-MS method and fractions containing the product were pooled together and lyophilized. The resulting product was then dissolved in 600 μL D_2O for NMR characterization. The product was further purified in a second round using an Agilent 1260 II preparative HPLC with a XSelect HSS Prep T3 column (5 μM, 19×150 mm). The mobile phase consisted of buffer A (0.1% formic acid in water) and buffer B (0.1% formic acid in acetonitrile) and was held decreased using a linear gradient from 97% to 95% buffer A for 5 min. Buffer A was then decreased from 95% to 60% over 10 min and then decreased to 40% and held constant for 4 min. The mobile was then returned to 97% buffer A using a linear gradient over 1 min. The flow rate was set to 10 mL/min at room temperature. Fractions (5 mL) were collected based on absorbance at 215 nm. Fractions were analyzed using the rapid HILIC LC/QTOF-MS method and fractions containing the product were pooled together and lyophilized. The resulting product was then dissolved in 600 μL D_2O for NMR characterization.

Assessing the bioactivity of Leu-Ala-Cit and Leu-Ala-Cit$_{alkyne}$. The bioactivity of Leu-Ala-Cit and Leu-Ala-Cit$_{alkyne}$ was determined following previously reported protocols [*20, 21*]. Briefly, *Pseudomonas* minimal medium (PMS, consisted of 1 g of ammonium dihydrogen orthophosphate, 0.2 g of potassium chloride, and 0.2 g of magnesium sulfate per 1 L, pH 7) molten agar (1.2 % w/v) was inoculated with an overnight *E. coli* K12 MG1655 starter (1 in 1000 dilution) and supplemented with 0.2% glucose. 1 mM Orn was added to the molten agar when needed. Once the agar was solidified, 10 μL of 50 μM peptide was added in triplicate to the solidified agar. Plates were incubated at 30ºC for 24 hours before analyzed for zones of inhibited growth.

***In vitro* MboABE assays in H_2O and D_2O to monitor alkyne tripeptide cyclization.** Assays (100 μL) contained 0.5 mM Leu-Ala-Arg, 2.5 mM NADH, 200 μM ferrous ammonium sulfate, 1 mM ascorbic acid, 15 μM MboA, and 0.5 μM MboB in 50 mM sodium phosphate pH 8.0 in H_2O or D_2O. When included, MboE was added at 1 μM final concentration. Reactions were incubated at room temperature for 1 h before quenching with one volume of MeCN or 1 % (*v/v*) formic acid in MeCN. Samples were incubated at room temperature for 10 min, 2 h, or overnight before analysis. Samples were centrifuged for 10 min at 21,000 × *g* at room temperature before analysis by LC/QTOF-MS using the HILIC method for *in vitro* Mbo assays.

***In vitro* production and purification of cyclized Leu-Ala-Cit$_{alkyne}$.** The reaction mixture (10 mL) contained 1 mM Leu-Ala-Arg, 5 mM NADH, 100 μM ferrous ammonium sulfate, 1 mM ascorbic acid in 500 mM ammonium formate pH 8.0. The reaction was initiated by addition of 10 μM MboA and 1 μM MboB. After incubation at room temperature for 2 h, 5 μM MboE was added and the reaction was incubated for an additional 2 h. The reaction was then quenched by addition of formic acid to a final concentration of 1% (*v/v*) and incubated overnight and room temperature lyophilized and resuspended in ddH_2O to a final volume of 2 mL. The crude mixture was centrifuged for 10 min at 21,000 × *g* at room temperature and any remaining soluble protein was removed using a Nanosep 10 kD MWCO centrifuge filter (Pall). Small particulates were removed using a hydrophobic PTFE syringe filter (13 mm, 0.45 μM; Avantor). The product was isolated from the mixture using an Agilent 1260 II preparative HPLC with a XBridge BEH Prep OBD Amide column (5 μm, 18×150 mm; Waters). The mobile phase consisted of buffer A (90% water, 10% acetonitrile and 10 mM ammonium formate pH 4.0) and buffer B (90% acetonitrile, 10% water and 10 mM ammonium formate pH 4.0) and was held for 1 min at 0% buffer A before

applying a linear gradient from 0% to 60% buffer A over 20 min. Buffer A was then increased linearly to 70% over 2 min and held constant for 2 min before returning the column to 0% buffer A using a linear gradient over 1 min. The flow rate was set to 20 ml/min at room temperature. Fractions (4 mL) were collected based on absorbance at 215 nm. Fractions were analyzed using the rapid HILIC LC/QTOF-MS method and fractions containing the product were pooled together and lyophilized. The resulting product was then dissolved in 600 µL D_2O for NMR characterization as the higher concentration allowed us to visualize low intensity ^1H-^{13}C HMBC HMBC cross peaks (**Fig. S14D**).

A second round of HPLC purification was carried out to remove the impurities with proton resonances ~ 3.5 ppm. The product was purified using an Agilent 1260 II preparative HPLC with a XSelect HSS Prep T3 column (5 µM, 19 x 150 mm). The mobile phase consisted of buffer A (0.1% formic acid in water) and buffer B (0.1% formic acid in acetonitrile) and was held decreased using a linear gradient from 97% to 95% buffer A for 5 minutes. Buffer A was then decreased from 95% to 60% over 10 minutes and then decreased to 40% and held constant for 4 minutes. The mobile was then returned to 97% buffer A using a linear gradient over 1 min. The flow rate was set to 10 ml/min at room temperature. Fractions (5 mL) were collected based on absorbance at 215 nm. Fractions were analyzed using the rapid HILIC LC/QTOF-MS method and fractions containing the product were pooled together and lyophilized. The resulting product was then dissolved in 600 µL D_2O for NMR characterization (*Figs. S12A-E* and *S12F*).

Crystallization of apo MboA and data collection. MboA crystals were prepared via the hanging drop vapor diffusion method. Hanging drops were prepared by combining equal volumes of MboA (18 mg/mL) protein solution and reservoir solution (1.2 M lithium sulfate, 0.1 M sodium acetate pH 4.6) for a total drop volume of 2 µL. Crystals typically grew in about approximately 1 to 2 weeks. Crystals were cryoprotected with the addition of 0.75 µL of 40% (*v/v*) glycerol in 0.1 M sodium acetate pH 4.6 and flash frozen in LN_2. Data were collected at Beamline 17-ID-2 at Brookhaven National Laboratory at a wavelength of 0.98 Å.

Crystallization of LAR-bound apo MboA and data collection. MboA crystals were prepared via the hanging drop vapor diffusion method. Hanging drops were prepared by combining equal volumes of MboA (9 mg/mL) protein solution and reservoir solution (0.4 M lithium sulfate, 0.1 M sodium acetate pH 4.8) for a total drop volume of 2 µL. The LAR-bound structure was prepared by incubating apo crystals with 0.3 µL of 100 mM LAR tripeptide in 0.1 M sodium acetate pH 4.6 for 2 h prior to looping. Data were collected at Beamline 17-ID-1 at Brookhaven National Laboratory at a wavelength of 0.92 Å.

Crystallization of LAR-bound Fe(II)$_2$-MboA and data collection. MboA crystals were prepared via the hanging drop vapor diffusion method in an anerobic Coy Chamber with an atmosphere of >5 ppm O_2. Hanging drops were prepared by combining equal volumes of MboA (9 mg/mL) protein solution, reservoir solution (0.6 M lithium sulfate, 0.1 M sodium acetate pH 4.6), and 0.2 µL of a 1:1000 dilution microseed master stock generated from apo crystals for a total drop volume of 2.2 µL. The Fe(II)- and LAR-bound structure was prepared by incubating apo crystals with 0.4 µL of 100 mM LAR tripeptide and 100 mM ammonium Fe(II) sulfate in 0.1 M sodium acetate pH 4.6 for 2 h prior to looping. Data were collected at Beamline 8.3.1 at the Advanced Light Source Facility at a wavelength of 1.11 Å. Due to radiation damage of the crystal, we were unable to acquire data at the Fe anomalous wavelength and provide instead an anomalous map calculated from the native wavelength data, which supports the presence of two heavy elements. Thus, we modeled in two ferrous ions.

Structure determination. Data were integrated with XDS [*22*] and scaled and merged with AIMLESS [*23*] within the CCP4 suite [*24*]. That data were phased via molecular replacement using the corresponding AlphaFold [*25*] MboA model. The structures were then refined iteratively in COOT [*26*] and Phenix [*27*]. Ligands were added to the model using COOT and refined in Phenix. F_o-F_c and POLDER [*28*] maps were calculated using Phenix. Structures and figures were analyzed and generated in ChimeraX [*29*].

Docking a binuclear metallocofactor into an HDO AlphaFold model. Metal-bound HDO models were generated by using the AlphaFold 3 server [*9*] by supplementing with two Co(II) ions as a surrogate for Fe(II) as Fe(II) is not available as an addable entity. Structures were manipulated and visualized using ChimeraX [*30*].

Figures and Tables

Table S1. Strains, plasmids, oligonucleotides, gene sequences, and amino acid sequences. (A) Strains and plasmids used in this study. (B) Oligonucleotides used for plasmid construction. (C) Protein sequences of synthetic genes. (D) DNA sequences for synthetic genes (codon-optimized for overexpression in *E. coli*).

A. Strains and plasmids

Strain	Description	Source
E. coli DH10b-T1^R	*F-mcrA Δ(mrr-hsdRMS-mcrBC) φ80lacZΔM15 ΔlacX74 recA1 endA1 araD139 Δ(ara-leu)7697 galU galK λrpsL(StrR) nupG tonA*	Thermo-Fisher
E. coli BL21 Star (DE3)	*F-ompT hsdSB (rB-, mB-) gal dcm rne131* (DE3)	Thermo-Fisher
**Plasmid**	**Description**	**Source**
pET16b-His$_{10}$-MboA	His$_{10}$-MboA (T7), *lacI*, Cb^R, ColE1	This study
pET16b-His$_{10}$-MboB	His$_{10}$-MboB (T7), *lacI*, Cb^R, ColE1	This study
pET16b-His$_{10}$-MboC	His$_{10}$-MboC (T7), *lacI*, Cb^R, ColE1	This study
pET16b-His$_{10}$-MboD	His$_{10}$-MboD (T7), *lacI*, Cb^R, ColE1	This study
pET16b-His$_{10}$-MboE	His$_{10}$-MboE (T7), *lacI*, Cb^R, ColE1	This study

B. Oligonucleotide sequences

Name	Sequence
pET16b MboA Fwd	TCTAGAAGTGCTTTTTCAGGGCCCGCATATGATTTCGACGGCTGAAATGC
pET16b MboA Rev	TCCTTTCGGGCTTTGTTAGCAGCCGGATCCTTAAAGAGTTGCGGCACGCAG
pET16b MboB Fwd	TCTAGAAGTGCTTTTTCAGGGCCCGCATATGGCAATTTACAATGTGCGTATTG
pET16b MboB Rev	TCCTTTCGGGCTTTGTTAGCAGCCGGATCCCTAACCGAACGATTCAAGCTCACAC
pET16b MboC Fwd	TCTAGAAGTGCTTTTTCAGGGCCCGCATATGTCCATCGTAGTTATCGACCC
pET16b MboC Rev	TCCTTTCGGGCTTTGTTAGCAGCCGGATCCTCATAAGATTCCGACTTCTGCGTG
pET16b MboD Fwd	TCTAGAAGTGCTTTTTCAGGGCCCGCATATGAAGCACGTTTTGGTTATTAACC
pET16b MboD Rev	TCCTTTCGGGCTTTGTTAGCAGCCGGATCCTCAAAGATCAGGAGGACGGATAC
pET16b MboE Fwd	TCTAGAAGTGCTTTTTCAGGGCCCGCATATGAACGTGCTTTTGCAGTTGC
pET16b MboE Rev	TCCTTTCGGGCTTTGTTAGCAGCCGGATCCTTATAAAAGTTGAGTCGCGCAGTG
pET16b Backbone Fwd	ATGCGGGCCCTGAAAAAGC
pET16b Backbone Rev	GGATCCGGCTGCTAACAAAGC

C. Protein sequences

MboA

>tr|A0A244EXR3|A0A244EXR3_PSESX Mangotoxin biosynthesis protein MboA OS=*Pseudomonas syringae* OX=317 GN=BW686_01200 PE=4 SV=1
MISTAEMLKTLETTVDTCLEAEFKKDTVMGEFHKGDWFNESYYKRHILECVIRIHMNNE
LDARAVQVAAIDNISAAKQLSYYLYDEFGHDEMFGQDLITYGYSASDIKAEFAFPETWK
LMGYLNFCVSKFGPLSSITWDWFLEYYGDKYSSFITQKASASMGQPAVSGAASHVAFD
EAEDHSGMMNNMLSSVIKDEKDLAKAVVHIKAFVPMVGEYFQALRAATL

MboB

>tr|A0A244EY36|A0A244EY36_PSESX Oxidoreductase OS=*Pseudomonas syringae* OX=317 GN=BW686_01205 PE=4 SV=1
MAIYNVRIGGQTYTAERQQPLTDAIPHEALVRGCLKGVCRVCKCTLVSGRVLEHGKAV
ALKDTFLPCVSHAETDIDIRAAISTFHAARLKSKKMLSGQVMEVVLEVKKVFYNAKSVI
TLKHPDVAALRSYSVVTLGGKDYDSLTCHVKLRAGGVFSALLEQLSVGDPLEYSIASPA
LPVYDDSLSCLNVVSGGSGMGAALSRAQELASKYNISEVAIYAINRSGLSDYHAGCIEAF
RQTVECNLQVTNFPFAEWTHADFDIGEHLLPNALTLGVGSEVVIGTLKNLPLCELESFG

MboC

>tr|A0A244EXU4|A0A244EXU4_PSESX Mangotoxin biosynthesis protein MboC OS=*Pseudomonas syringae* OX=317 GN=BW686_01210 PE=4 SV=1
MSIVVIDPVSSGISYIHAAQQLGVDVYVFSSDGGEQELNAELRAKVRQVISIDTGDFDVQ
LKKLNELGNIRAVLPGVEYAVPMAARLGAASGTTHLNDSAVEKVRNKFNFRSRLTDVG
LSSIGFFLLDPQAPVSVPEGFSFPAVVKPIDMAGSIGVRKLYSHEELVEAVQAFRQSLPDD
IGFTASGRLIVEEYIPGNEYSVEGIVRNDGSITVASITEKLLGSEPYFVEVGHIVGQGYEEA
FRTTLTDYSLAVLDAINLNVGPFHLELRVTPQGQPVAVELAARLPGDNIVELIKRASGID
LAKETLCEYLNIDSPLVPRANGVSAIAFIPRGDKSEFTELRGLSDIFDSPYYVSHQVYYRS
GDAMGSDQDWTSRIGYVMFGGTDEEVVRGLVGRVHAEVGIL

MboD

>tr|A0A244EXY0|A0A244EXY0_PSESX Biotin carboxylase OS=*Pseudomonas syringae* OX=317 GN=BW686_01215 PE=4 SV=1
MKHVLVINRYDDELSDYRKYIDHSQVDVSYISLAGQSRLIDPQVSATVVEVSALDADLIL
QEARAIHRRQPIDFVIAFSEYDLDAAALVRTEFNIRGAKVADNLLCRNKASMKEALTGS
SVRYPQYRNVVSRGGVEAFCHEHGYPVILKPQVGAASDGVVKIERPEDIPDLPDFNGYE
VEEYIEGDIFHVDAILAAGSMPYFKVSKYLNTCLDFRNGLPLGSVTVDNPAFIERVRAFT
EEVCVRLNLRNQAIHLEIIERRGELVFLEIGGRVGGGEIPFVALRSEGIDLFELWTRAALEI
AIAPVETRITGFLMMPNPFPGGFTFDPAMELSHPLLSYSSVQSHGGSNGFSYEDIPARLHF
TADSQAEVERAVLQCMDILQTSIRPPDL

MboE

>tr|A0A244EXR2|A0A244EXR2_PSESX Amidinotransferase OS=*Pseudomonas syringae* OX=317 GN=BW686_01220 PE=4 SV=1
MNVLLQLPSVVQPVSMSVFMEAQQPYSLRQADADVMALAGSLNAAGIPTIFLSDALFSE
EGPHVAIEDMRRLAARALRFADNVEQTRQQLAHEQLARCSRQELAEIIINQPELCLHYD
DELGRVSPDATYESYRIQPLYGLLFPRDHFMSVGGQPVFGRLKRQDRAREVDVVKAVV

AAHGHVPVELDQVLEGGDYQENARVSVINTGFRTDAAVLTFLLASSLLKGDVVLAVQD
VGCNPEQFHLDHYACLLRDALLIDAKRADAVDRSRVSVYRRTPAGWHLTHEGLTLRQA
AQVAEVEPIVLDDEMMAAWCANAFSWGNHVWIDQHAPSRLLDQLAGRDGQVHLIAFT
EHQKQFGGIHCATQLL

D. DNA sequences of synthetic genes (codon-optimized for *E. coli* expression)

MboA
ATGATTTCGACGGCTGAAATGCTGAAGACTTTGGAGACGACAGTAGACACGTGTTTG
GAGGCTGAATTCAAGAAGGACACCGTTATGGGGGAGTTCCACAAAGGCGATTGGTT
CAACGAAAGTTATTACAAGCGTCATATTCTGGAGTGCGTCATCCGCATTCATATGAA
CAACGAGTTAGATGCGCGTGCGGTACAGGTAGCGGCAATTGATAATATTTCGGCGG
CAAAACAACTGTCGTATTACTTATATGATGAGTTTGGACATGATGAAATGTTCGGGC
AAGACCTTATCACCTACGGTTATTCAGCCTCAGATATTAAGGCGGAATTCGCATTTC
CAGAAACGTGGAAACTGATGGGATATTTGAACTTTTGTGTCAGTAAATTCGGGCCTT
TGTCAAGCATCACGTGGGACTGGTTCTTAGAATACTATGGTGACAAATACAGTTCTT
TTATCACCCAAAAGGCAAGCGCGAGTATGGGACAACCAGCCGTTTCCGGCGCAGCG
AGCCACGTCGCGTTTGATGAGGCTGAGGACCATTCGGGGATGATGAACAATATGTT
AAGCTCAGTTATCAAAGACGAAAAGGACTTAGCGAAAGCAGTAGTCCACATCAAGG
CATTCGTCCCGATGGTGGGCGAATATTTCCAAGCACTGCGTGCCGCAACTCTTTAA

MboB
ATGGCAATTTACAATGTGCGTATTGGCGGGCAAACTTATACAGCCGAGCGCCAGCA
ACCTCTGACTGACGCAATCCCCCATGAAGCATTAGTGCGTGGATGCCTGAAGGGTGT
ATGTCGCGTGTGTAAATGCACATTGGTTTCGGGGCGTGTACTGGAGCACGGCAAAGC
AGTCGCTTTGAAGGATACGTTCTTGCCGTGCGTATCTCATGCGGAAACGGACATTGA
TATTCGTGCAGCGATCTCGACTTTCCACGCTGCGCGTCTGAAGTCGAAGAAGATGTT
ATCTGGTCAAGTTATGGAAGTCGTCTTAGAGGTTAAGAAGGTCTTCTACAATGCGAA
GTCGGTCATCACGCTGAAGCATCCAGACGTCGCGGCATTACGCTCTTACAGCGTAGT
CACATTGGGGGGAAAAGATTATGATTCACTTACTTGCCATGTTAAACTGCGTGCGGG
GGGTGTTTTCTCCGCGCTTCTGGAGCAGTTGTCCGTGGGAGACCCTTTAGAATATTCC
ATTGCCAGCCCAGCTTTGCCGGTTTACGATGATAGCCTTTCATGCTTGAACGTTGTAT
CTGGGGGTTCGGGGATGGGTGCTGCTTTAAGCCGCGCGCAAGAGCTTGCATCAAAG
TACAACATTTCAGAAGTCGCTATCTATGCTATCAACCGCAGCGGTTTGTCGGACTAC
CATGCTGGTTGTATTGAGGCATTTCGCCAGACCGTTGAGTGCAACTTACAAGTCACG
AATTTCCCGTTTGCCGAATGGACCCATGCGGACTTTGACATTGGAGAACATTTATTA
CCCAACGCATTGACTCTGGGGGTTGGCAGCGAGGTCGTCATCGGAACATTAAAGAA
TCTGCCCTTGTGTGAGCTTGAATCGTTCGGTTAG

MboC
ATGTCCATCGTAGTTATCGACCCGGTGTCATCAGGAATTTCTTATATCCATGCCGCGC
AACAATTAGGCGTCGATGTCTACGTTTTTTCCTCTGATGGTGGGGAACAAGAATTGA
ACGCCGAATTGCGCGCCAAAGTTCGCCAGGTAATTTCGATCGATACGGGGGACTTCG
ATGTACAACTTAAGAAGTTAAATGAATTAGGAAATATCCGCGCCGTACTTCCAGGCG
TAGAGTATGCTGTTCCTATGGCGGCACGCCTTGGGGCTGCGTCCGGCACAACACATT
TGAATGATTCCGCTGTGGAGAAGGTGCGTAATAAGTTCAATTTCCGCTCCCGTCTTA
CCGACGTGGGGTTGTCGTCAATTGGATTTTTCCTTTTGGATCCACAGGCACCAGTATC

AGTACCCGAAGGCTTTTCCTTCCCGGCCGTGGTGAAGCCTATCGACATGGCAGGAAG
TATCGGAGTCCGTAAGCTGTACAGCCACGAGGAGCTGGTTGAAGCCGTGCAGGCGT
TCCGTCAAAGTCTGCCGGACGATATTGGATTCACGGCTAGTGGCCGCTTAATCGTGG
AGGAGTATATTCCAGGGAACGAGTATAGTGTAGAGGGAATCGTCCGTAATGACGGG
TCAATCACGGTGGCGTCGATTACGGAAAAACTTTTAGGGTCAGAACCTTATTTCGTT
GAGGTCGGACATATTGTAGGCCAAGGTTACGAAGAGGCCTTTCGCACGACGTTGAC
TGACTATTCATTGGCCGTCCTGGATGCGATCAACTTAAACGTAGGTCCCTTTCACTTA
GAGTTACGTGTCACCCCACAAGGCCAACCTGTCGCGGTAGAGTTGGCTGCCCGTCTG
CCTGGAGATAATATTGTGGAGTTAATCAAACGTGCCAGCGGAATCGACTTAGCGAA
AGAAACATTGTGTGAGTACTTGAACATCGACTCTCCCCTTGTTCCGCGCGCAAATGG
AGTCAGTGCGATTGCTTTCATCCCGCGCGGTGACAAATCGGAGTTTACCGAACTTCG
CGGACTTAGTGACATTTTCGATTCGCCGTACTACGTTTCGCACCAAGTTTACTATCGT
AGTGGAGACGCTATGGGCTCTGACCAGGATTGGACCAGTCGCATCGGTTACGTGAT
GTTTGGTGGGACTGACGAAGAGGTGGTCCGCGGATTAGTGGGCCGTGTGCACGCAG
AAGTCGGAATCTTATGA

MboD
ATGAAGCACGTTTTGGTTATTAACCGCTATGACGACGAATTATCAGACTATCGCAAA
TATATCGACCACTCCCAGGTTGACGTTTCGTACATCTCCTTAGCAGGGCAGAGCCGC
CTTATCGATCCGCAAGTTTCCGCTACTGTTGTTGAGGTATCCGCTCTGGACGCGGATC
TTATTCTGCAAGAGGCGCGCGCAATCCATCGTCGCCAGCCAATCGATTTCGTGATCG
CTTTTAGCGAATACGACCTGGATGCAGCTGCTCTGGTACGTACCGAATTCAATATTC
GTGGCGCCAAGGTAGCCGACAATCTTCTGTGCCGTAACAAAGCCTCAATGAAGGAG
GCCCTTACAGGATCTTCTGTGCGCTACCCACAGTATCGTAATGTAGTCTCACGCGGT
GGCGTAGAGGCATTTTGCCACGAACACGGGTATCCTGTTATCCTGAAACCGCAAGTC
GGAGCGGCTTCGGATGGCGTAGTAAAAATTGAACGTCCCGAGGATATCCCCGACTT
ACCCGATTTTAATGGTTATGAAGTTGAGGAGTACATCGAGGGAGACATCTTTCATGT
CGACGCCATTTTGGCAGCGGGTTCAATGCCTTATTTCAAAGTTAGTAAATATTTGAA
TACTTGCCTTGACTTTCGTAACGGTTTGCCCCTGGGTTCCGTTACCGTGGATAATCCA
GCATTTATCGAGCGTGTACGCGCGTTTACCGAAGAGGTTTGTGTCCGTTTGAATTTA
CGCAATCAGGCAATCCACTTGGAAATTATTGAACGCCGTGGAGAGCTGGTGTTCCTG
GAAATTGGAGGGCGTGTTGGAGGCGGAGAAATTCCCTTCGTTGCATTACGTTCAGAG
GGCATTGATCTTTTTGAGTTATGGACGCGCGCAGCTTTGGAAATTGCTATTGCCCCA
GTGGAAACCCGCATCACAGGCTTCTTAATGATGCCGAATCCCTTTCCTGGGGGGTTT
ACTTTTGACCCAGCGATGGAGTTAAGTCACCCGTTACTGAGCTATAGCTCAGTACAA
TCGCACGGGGGCTCCAATGGTTTTTCCTATGAAGACATTCCGGCGCGTTTACACTTC
ACCGCCGATTCACAAGCTGAAGTCGAGCGCGCAGTCCTTCAGTGCATGGATATCCTG
CAAACTAGTATCCGTCCTCCTGATCTTTGA

MboE
ATGAACGTGCTTTTGCAGTTGCCATCAGTGGTTCAGCCAGTCTCTATGAGTGTATTCA
TGGAGGCCCAACAACCCTATTCCTTGCGCCAAGCTGACGCAGACGTAATGGCATTA
GCAGGTAGTTTGAATGCCGCCGGCATTCCTACTATCTTCTTGTCAGATGCCTTATTCT
CGGAGGAAGGCCCGCATGTAGCGATCGAGGACATGCGCCGCTTAGCGGCCCGTGCG
TTGCGCTTTGCAGATAATGTAGAGCAAACGCGCCAGCAGTTAGCTCATGAACAGTTA
GCCCGCTGTTCGCGTCAAGAGTTAGCAGAAATCATTATTAACCAGCCCGAGTTGTGC

CTGCACTATGATGATGAGTTGGGCCGCGTCAGCCCCGACGCAACCTACGAGAGTTA
CCGTATCCAGCCGCTGTACGGGCTTCTGTTTCCTCGCGATCACTTTATGTCTGTGGGC
GGACAGCCCGTCTTTGGGCGTTTAAAGCGTCAGGACCGCGCCCGTGAGGTTGACGTT
GTGAAGGCCGTGGTCGCTGCGCATGGGCATGTCCCAGTGGAATTGGACCAAGTATT
AGAAGGTGGAGATTATCAGGAAAATGCGCGCGTCTCCGTAATTAACACAGGATTTC
GCACGGACGCGGCAGTCCTGACCTTTCTTTTAGCATCAAGCCTGCTTAAGGGCGACG
TTGTTCTTGCCGTTCAGGACGTTGGTTGTAACCCCGAACAGTTCCATTTAGACCACTA
CGCTTGCCTTCTTCGCGATGCCTTGTTGATCGACGCTAAACGCGCGGACGCCGTAGA
TCGCTCTCGCGTATCGGTGTATCGTCGTACACCAGCAGGCTGGCACCTTACCCATGA
GGGCCTGACTCTGCGTCAGGCTGCCCAGGTGGCTGAGGTGGAACCAATCGTCTTAGA
TGACGAGATGATGGCAGCTTGGTGCGCTAACGCTTTTAGCTGGGGTAATCACGTCTG
GATCGACCAGCACGCACCATCTCGCTTACTGGATCAGCTTGCCGGCCGTGATGGGCA
AGTGCATCTTATTGCGTTTACTGAGCACCAAAAGCAATTCGGCGGCATCCACTGCGC
GACTCAACTTTTATAA

Figure S1. Bioinformatic workflow to identify HDO sequences by structural analysis. Sequences from the IPR016084 family were assessed for their likelihood of being an HDO with a diiron center by structural alignment of their AlphaFold models to seven reference HDOs (SznF, UndA, CADD, FlcE, AetD, PolF and BesC). This structural alignment was used to tabulate possible metal-binding residues in the candidate or target model. After tabulation of the data with respect to all reference HDOs, HDOs were binned based on the number of predicted metal-binding residues as well as their deviation in location from the reference metal-binding residue. Using this analysis, 20,866 HDOs were identified from the 63,711 available AlphaFold models corresponding to the members of IPR016084. The remaining members without AlphaFold models were then analyzed by sequence alignment alone.

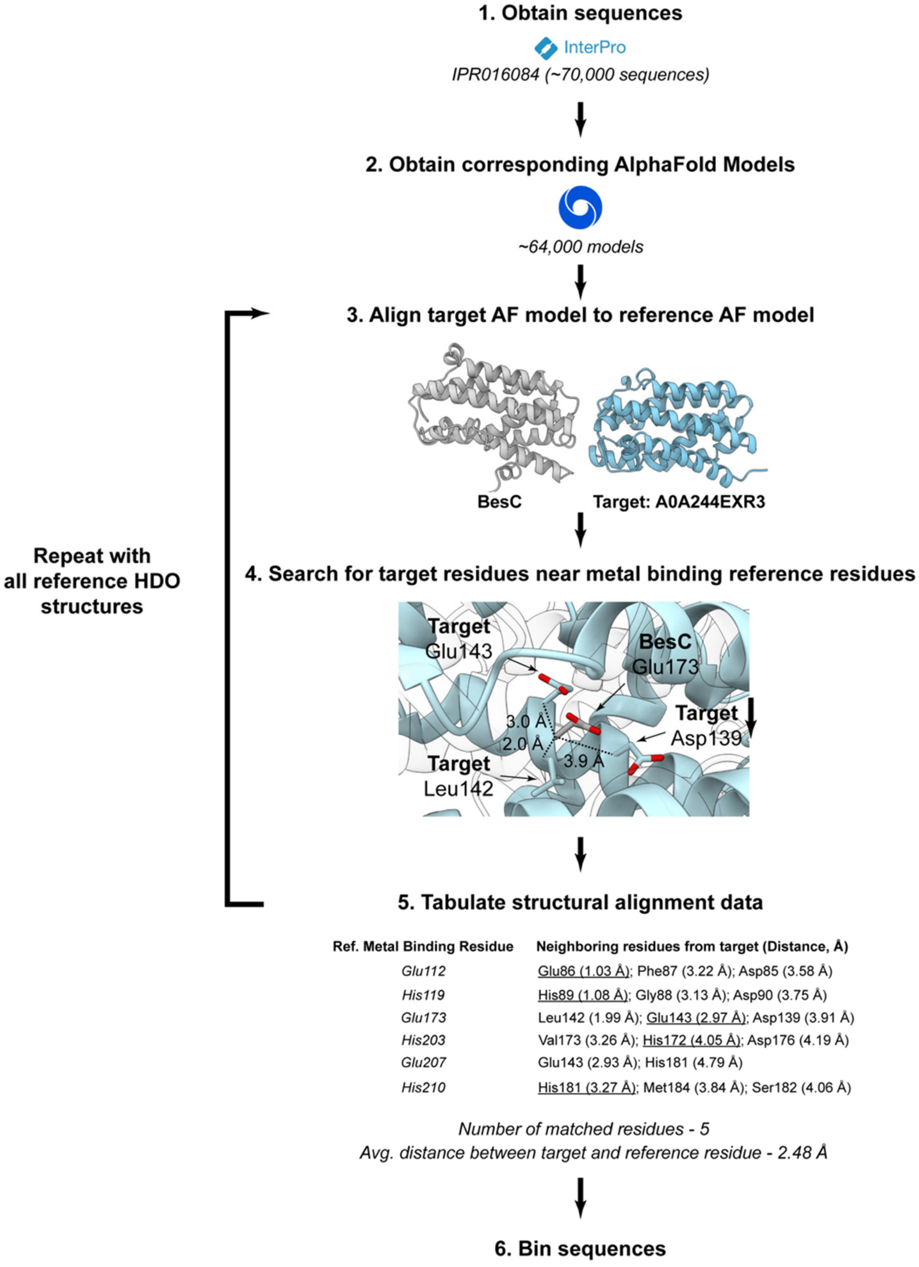

Figure S2. Bioinformatic analysis of IPR016084 and IPR045776 members with a predicted bimetallic site. (A) SSN of sequences from IPR016084 and IPR045776. Edges correspond to sequences above an alignment threshold of 42 (~35% sequence identity). Sequences sharing >50% sequence identity are collapsed into single nodes (18,927 nodes). Nodes that reflect sequences that are predicted to bind a bimetallic site based on our structural analysis are highlighted in purple. Nodes that reflect sequences that are predicted to bind a bimetallic site based on our sequence analysis are diamond shaped. Note that all this information is available in a supplementary .tsv file. (B) SNN of sequences predicted to bind a binuclear metallocofactor based on our structural analysis. Once metal-binding sequences were identified, their neighboring genes were analyzed using the EFI tools [*13*]. Edges correspond to sequences above an alignment threshold of 42 (~40% sequence identity). Sequences sharing >65% sequence identity are collapsed into single nodes (8,754 nodes). Nodes highlighted in red indicate putative HDOs that neighbor an ATP-grasp. Clusters highlighted in panel C are circled. (C) List of putative biosynthetic gene clusters (BGCs) containing HDOs that are colocalized with ATP-grasps as observed from the EFI-GNT. This list includes SznF and BesC. (D) A more detailed annotation of representative BGCs from Panel C.

A

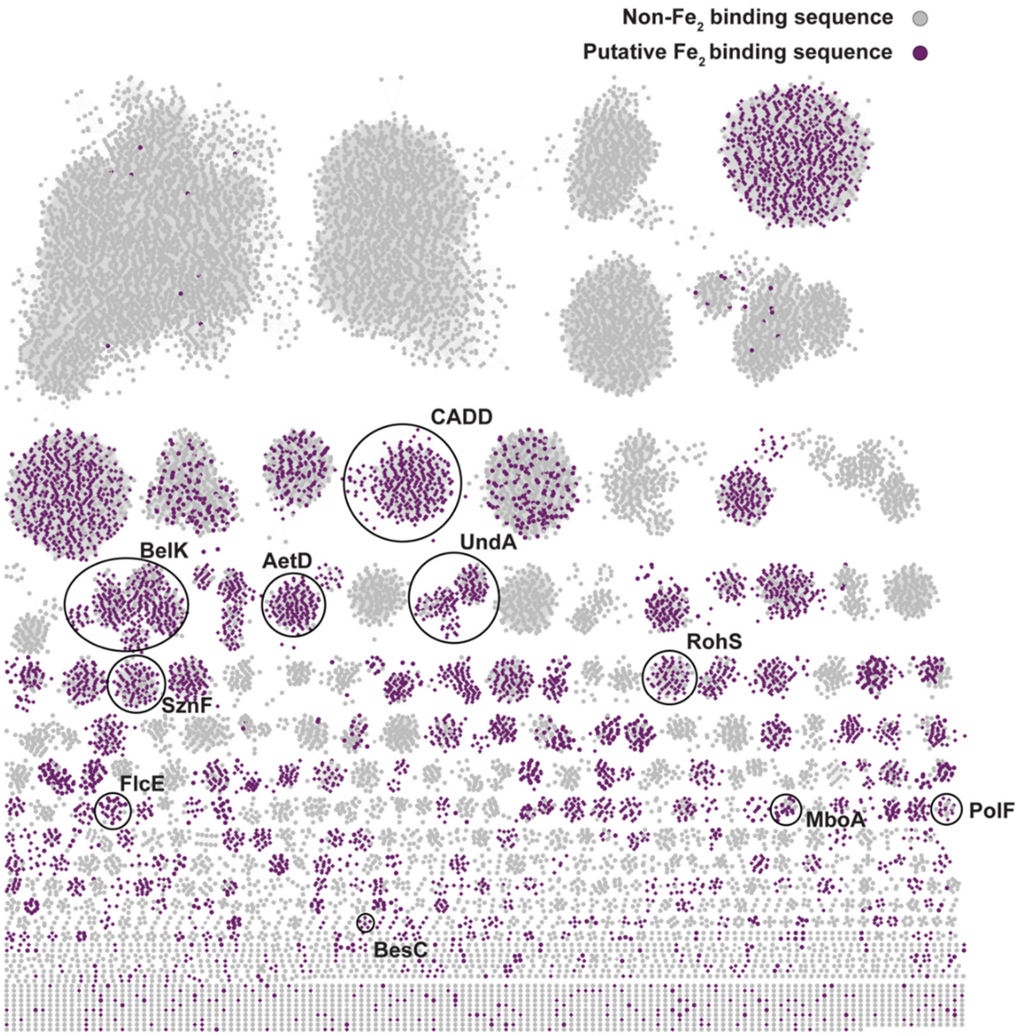

B

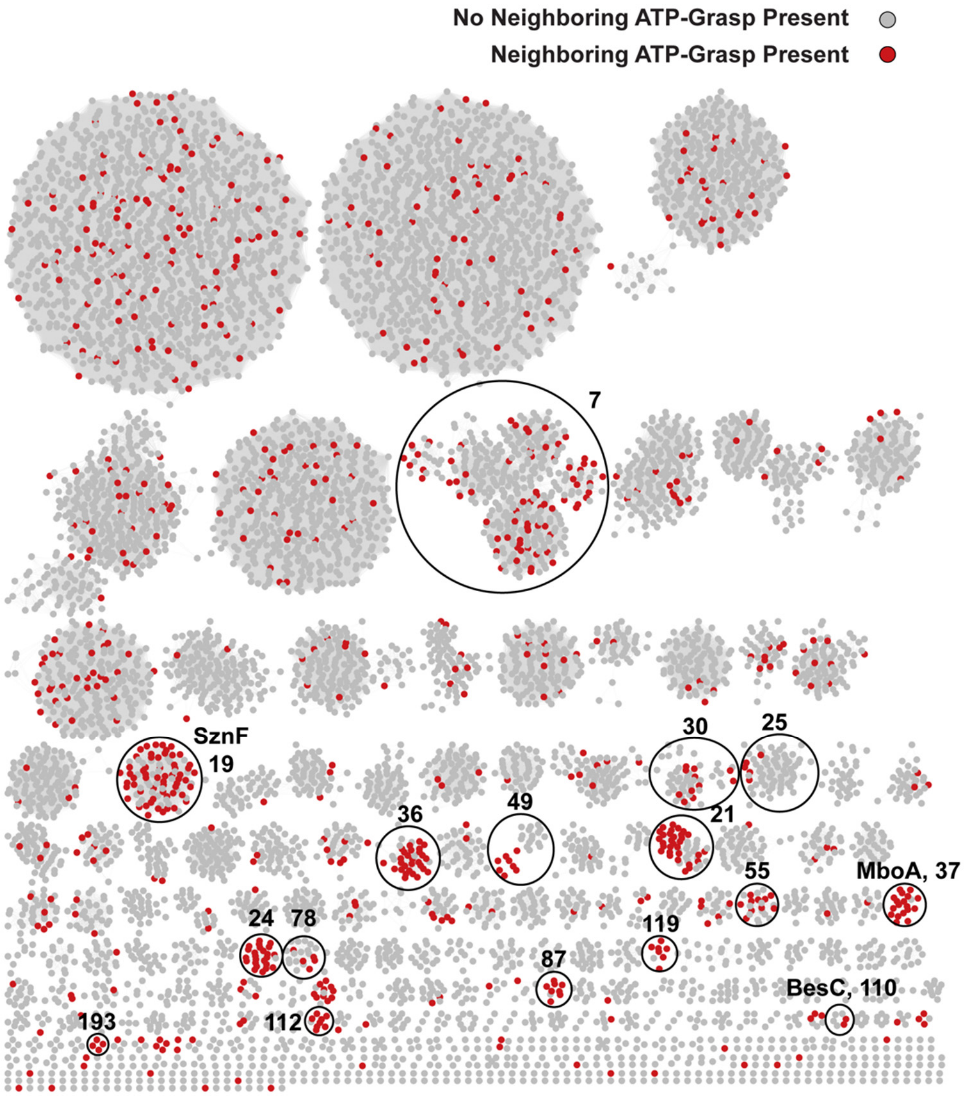

No Neighboring ATP-Grasp Present
Neighboring ATP-Grasp Present
7
SznF
19
30
25
36
49
21
55
MboA, 37
24
78
119
87
BesC, 110
193
112

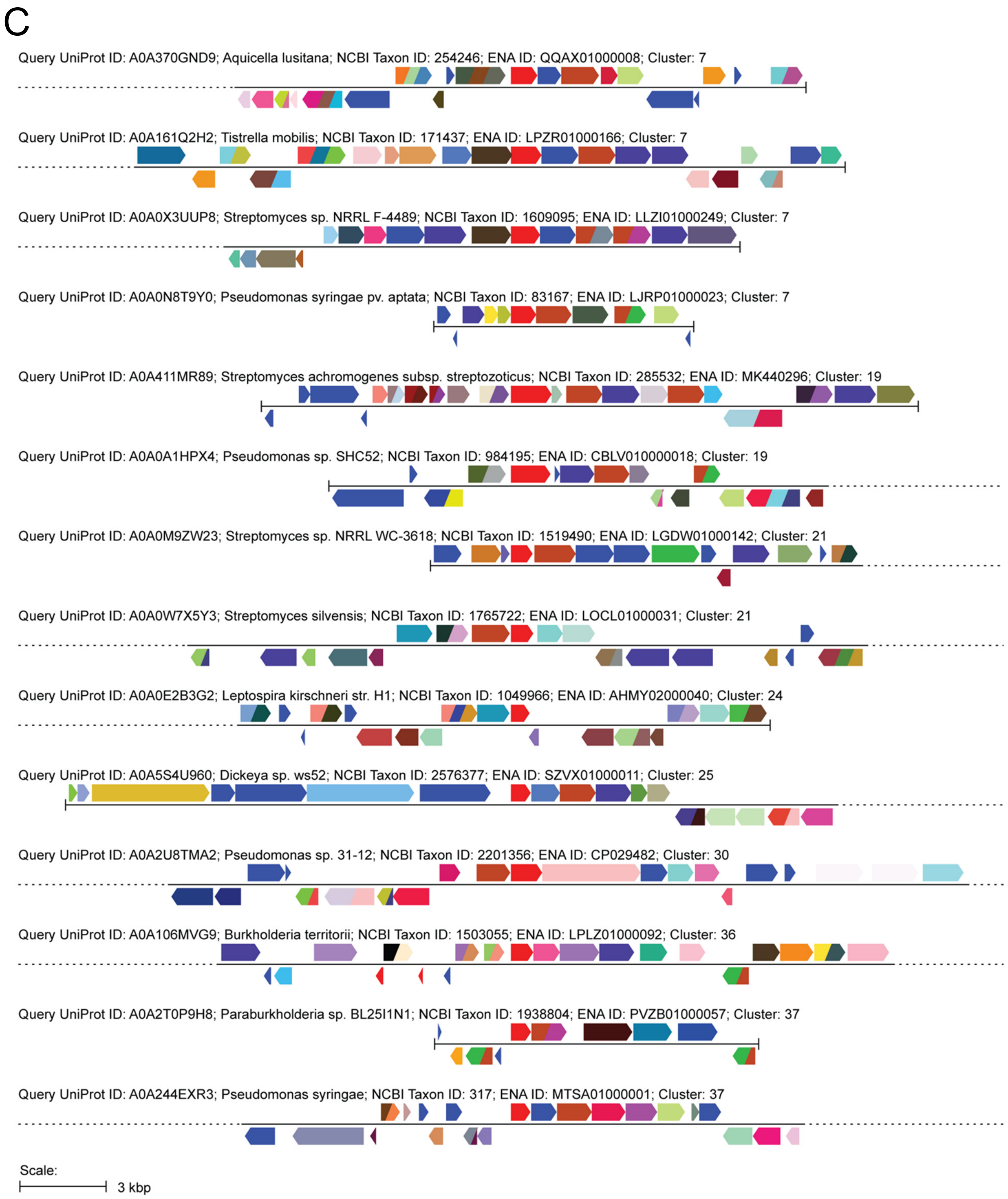
C
Query UniProt ID: A0A370GND9; Aquicella lusitana; NCBI Taxon ID: 254246; ENA ID: QQAX01000008; Cluster: 7
Query UniProt ID: A0A161Q2H2; Tistrella mobilis; NCBI Taxon ID: 171437; ENA ID: LPZR01000166; Cluster: 7
Query UniProt ID: A0A0X3UUP8; Streptomyces sp. NRRL F-4489; NCBI Taxon ID: 1609095; ENA ID: LLZI01000249; Cluster: 7
Query UniProt ID: A0A0N8T9Y0; Pseudomonas syringae pv. aptata; NCBI Taxon ID: 83167; ENA ID: LJRP01000023; Cluster: 7
Query UniProt ID: A0A411MR89; Streptomyces achromogenes subsp. streptozoticus; NCBI Taxon ID: 285532; ENA ID: MK440296; Cluster: 19
Query UniProt ID: A0A0A1HPX4; Pseudomonas sp. SHC52; NCBI Taxon ID: 984195; ENA ID: CBLV010000018; Cluster: 19
Query UniProt ID: A0A0M9ZW23; Streptomyces sp. NRRL WC-3618; NCBI Taxon ID: 1519490; ENA ID: LGDW01000142; Cluster: 21
Query UniProt ID: A0A0W7X5Y3; Streptomyces silvensis; NCBI Taxon ID: 1765722; ENA ID: LOCL01000031; Cluster: 21
Query UniProt ID: A0A0E2B3G2; Leptospira kirschneri str. H1; NCBI Taxon ID: 1049966; ENA ID: AHMY02000040; Cluster: 24
Query UniProt ID: A0A5S4U960; Dickeya sp. ws52; NCBI Taxon ID: 2576377; ENA ID: SZVX01000011; Cluster: 25
Query UniProt ID: A0A2U8TMA2; Pseudomonas sp. 31-12; NCBI Taxon ID: 2201356; ENA ID: CP029482; Cluster: 30
Query UniProt ID: A0A106MVG9; Burkholderia territorii; NCBI Taxon ID: 1503055; ENA ID: LPLZ01000092; Cluster: 36
Query UniProt ID: A0A2T0P9H8; Paraburkholderia sp. BL25I1N1; NCBI Taxon ID: 1938804; ENA ID: PVZB01000057; Cluster: 37
Query UniProt ID: A0A244EXR3; Pseudomonas syringae; NCBI Taxon ID: 317; ENA ID: MTSA01000001; Cluster: 37
Scale:
3 kbp

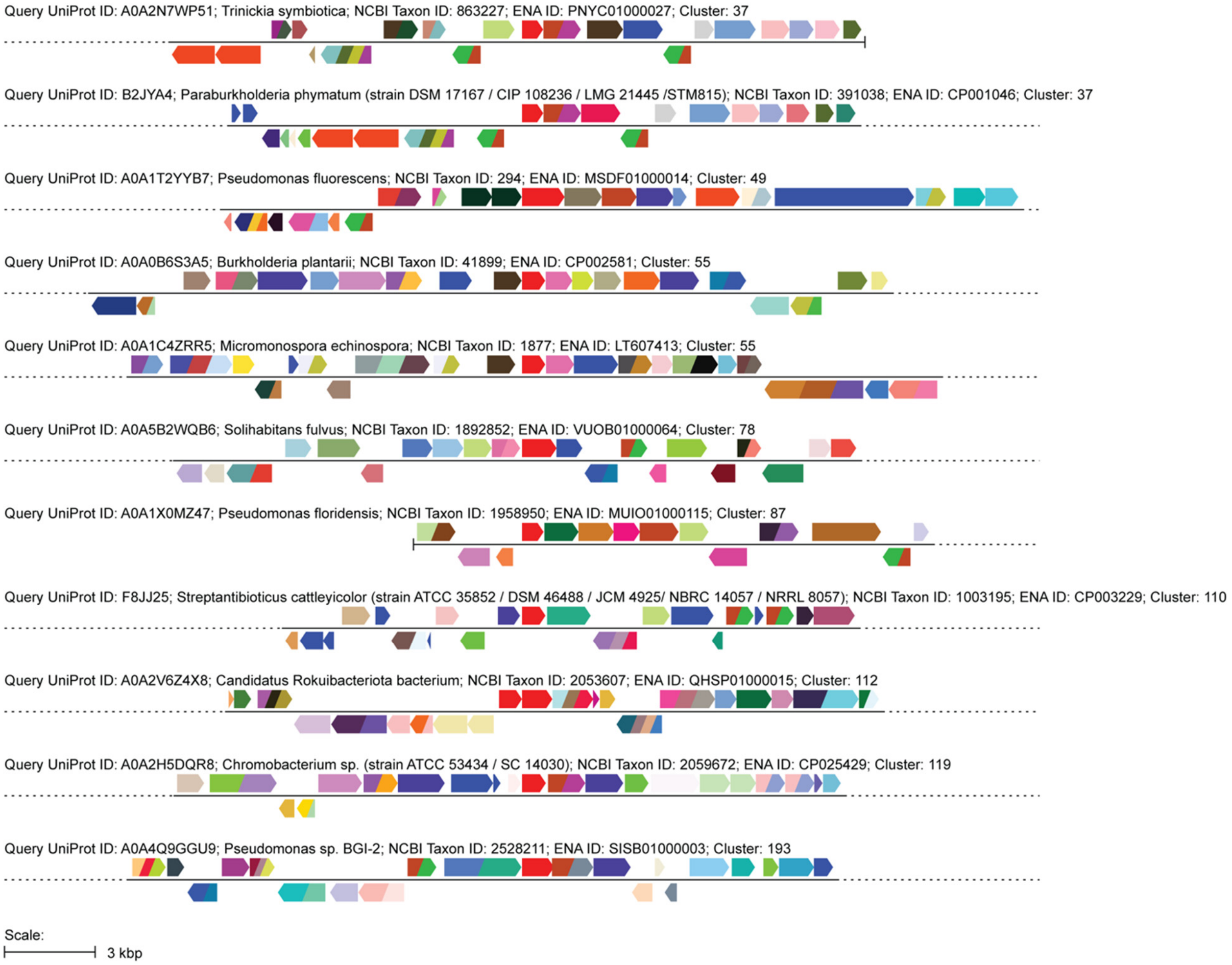
Query UniProt ID: A0A2N7WP51; Trinickia symbiotica; NCBI Taxon ID: 863227; ENA ID: PNYC01000027; Cluster: 37
Query UniProt ID: B2JYA4; Paraburkholderia phymatum (strain DSM 17167 / CIP 108236 / LMG 21445 /STM815); NCBI Taxon ID: 391038; ENA ID: CP001046; Cluster: 37
Query UniProt ID: A0A1T2YYB7; Pseudomonas fluorescens; NCBI Taxon ID: 294; ENA ID: MSDF01000014; Cluster: 49
Query UniProt ID: A0A0B6S3A5; Burkholderia plantarii; NCBI Taxon ID: 41899; ENA ID: CP002581; Cluster: 55
Query UniProt ID: A0A1C4ZRR5; Micromonospora echinospora; NCBI Taxon ID: 1877; ENA ID: LT607413; Cluster: 55
Query UniProt ID: A0A5B2WQB6; Solihabitans fulvus; NCBI Taxon ID: 1892852; ENA ID: VUOB01000064; Cluster: 78
Query UniProt ID: A0A1X0MZ47; Pseudomonas floridensis; NCBI Taxon ID: 1958950; ENA ID: MUIO01000115; Cluster: 87
Query UniProt ID: F8JJ25; Streptantibioticus cattleyicolor (strain ATCC 35852 / DSM 46488 / JCM 4925/ NBRC 14057 / NRRL 8057); NCBI Taxon ID: 1003195; ENA ID: CP003229; Cluster: 110
Query UniProt ID: A0A2V6Z4X8; Candidatus Rokuibacteriota bacterium; NCBI Taxon ID: 2053607; ENA ID: QHSP01000015; Cluster: 112
Query UniProt ID: A0A2H5DQR8; Chromobacterium sp. (strain ATCC 53434 / SC 14030); NCBI Taxon ID: 2059672; ENA ID: CP025429; Cluster: 119
Query UniProt ID: A0A4Q9GGU9; Pseudomonas sp. BGI-2; NCBI Taxon ID: 2528211; ENA ID: SISB01000003; Cluster: 193
Scale:
3 kbp

D

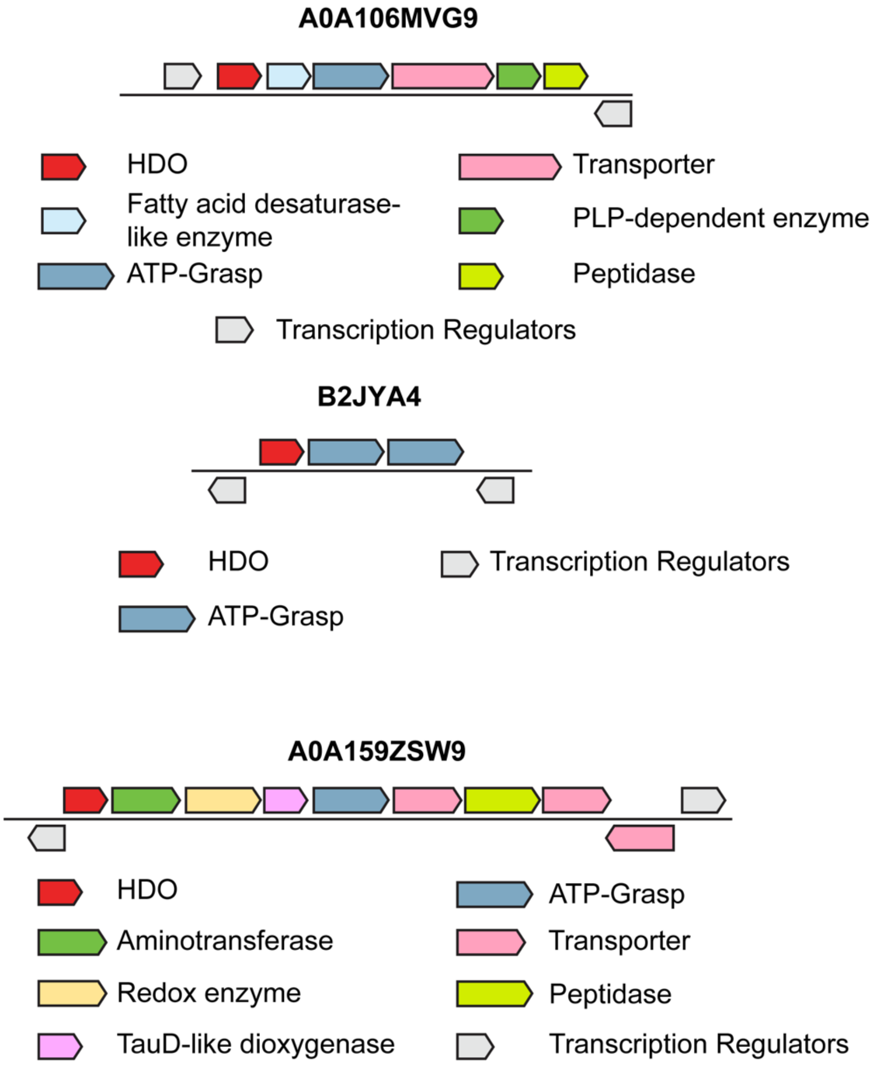
A0A106MVG9
HDO
Fatty acid desaturase-like enzyme
ATP-Grasp
Transporter
PLP-dependent enzyme
Peptidase
Transcription Regulators
B2JYA4
HDO
ATP-Grasp
Transcription Regulators
A0A159ZSW9
HDO
Aminotransferase
Redox enzyme
TauD-like dioxygenase
ATP-Grasp
Transporter
Peptidase
Transcription Regulators

Figure S3. Representative SDS PAGE gel of purified Mbo proteins. SDS-PAGE gel with purified MboA, MboB, MboC, MboD, MboE with a molecular weight standard ladder (kD).

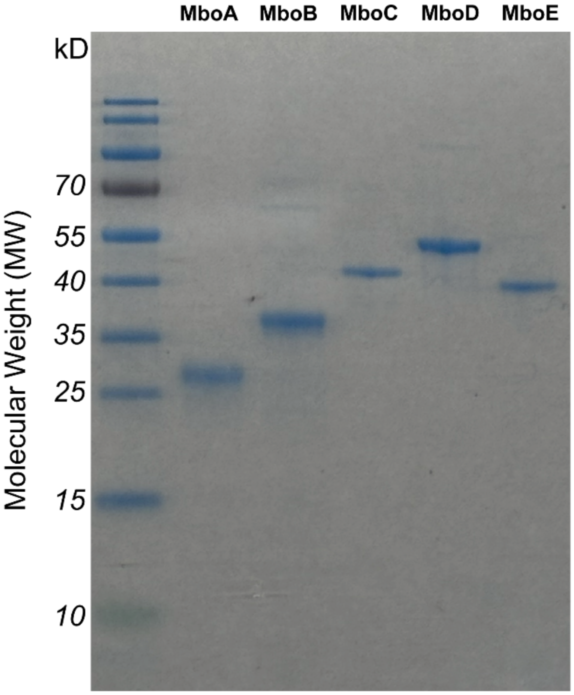

Figure S4. Analysis of *in vitro* MboC, MboD, and MboCD screening assays. The selectivity of the ATP-grasp enzymes, MboC and MboD, was screened *in vitro* using an assay including a mix of the 20 proteinogenic amino acids and L-ornithine (1 mM) with ATP (5 mM) and $MgCl_2$ (5 mM) and analyzed by LC/QTOF-MS. The data was visualized by plotting a heatmap of the ion abundance for each $[M+H]^+$ consistent for a given dipeptide extracted from each chromatogram using MS-Dial. [$^{13}C_6$, ^{15}N]-L-Ile is used to distinguish between L-Leu and L-Ile-containing products. Note that the heat map does not contain any information on the order of each amino acid. These data are representative of n = 3 technical replicates. (A) Heat map for MboC. The dipeptides that contain Ala/Arg, Asn/Leu, and Gln /Val appear to have the same abundance because they exhibit similar $[M+H]^+$ ions within the allowed error (35 ppm). (B) MS spectrum extracted at t_R = 0.76 min, when the ion with m/z $[M+H]^+$ = 246.1530 (246.1561) elutes. The y_1 fragment ion for Ala-Arg (m/z_{obs} = 175.1118 (175.1190)) is highlighted in red, leading to the assignment of this species as Ala-Arg. Observed ions are reported with m/z_{calc} in parentheses. (C) Heat map for MboD. The overall counts for all dipeptide combinations were low, suggesting that MboD activity was quite low in this assay. (D) Heat map for MboCD. The plot generated for dipeptides shows that Ala/Arg has the highest response rate, indicating that there are several tripeptides in high abundance that contain Ala and Arg. The next most abundant pairs are Leu/Ala, Ile/Ala, Leu/Arg, and Ile/Arg. Together, these data indicate that the two highest abundant tripeptides made by MboCD contain Leu/Ile, Ala, and Arg.

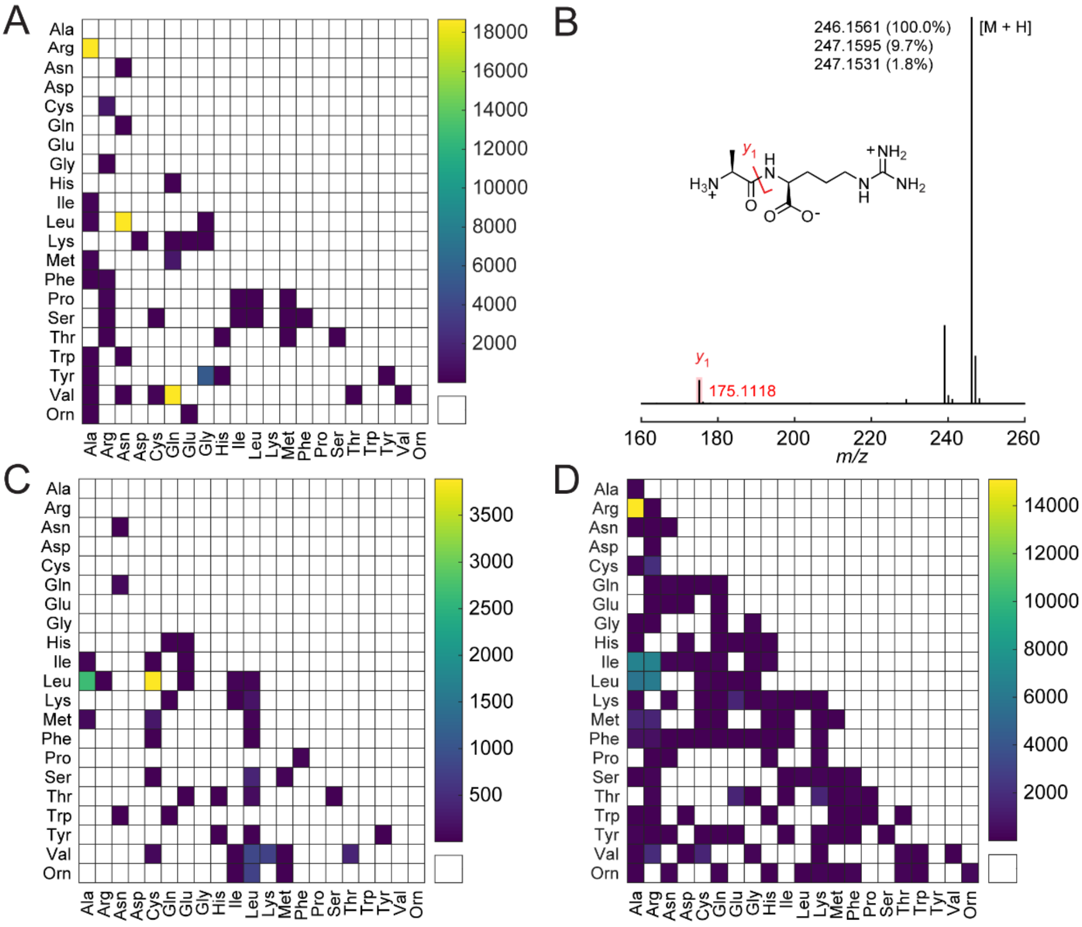

Figure S5. Characterization of the product of *in vitro* MboCDE reconstitution. The activity of MboE was screened *in vitro* using an assay including a mix of 20 proteinogenic amino acids and L-ornithine (1 mM) with ATP (5 mM) and $MgCl_2$ (5 mM) and analyzed by LC/QTOF-MS. MboC and MboD were also included when noted. [$^{13}C_6$, ^{15}N]-L-Ile is used to distinguish between L-Leu and L-Ile-containing products. (A) EIC from the MboCDE screen where each trace represents the sum of extracted ions for Ala-Arg (*m/z* = 246.1533), Leu-Ala-Arg (*m/z* = 359.2407) and Leu-Ala-Cit (*m/z* = 360.2243). Data are representative of traces for technical replicates (n = 3). (B) Comparison of the EICs from the MboCDE screen for Leu-Ala-Cit (*m/z* = 360.2243), Ile-Ala-Cit (*m/z* = 367.2399), and Met-Ala-Cit (*m/z* = 378.1793). From this analysis, Leu-Ala-Cit appears to be the preferred product. Data are representative of traces for technical replicates (n = 3). (C) MS/MS spectra supporting the assignment of the MboCDE product as Leu-Ala-Cit. When [$^{13}C_6$, $^{15}N_4$]-Arg is included, we observe the expected labeling in the y_2 and z_1 ions, which both contain atoms derived from L-Arg. The b_2 fragment ion does not contain atoms from L-Arg and thus does not demonstrate a shift in *m/z*. Please note that the y_1 fragment ion (m/z_{calc} = 185.1143) for [$^{13}C_6$, $^{15}N_3$]-LACit is obscured by the b_2 fragment ion (m/z_{obs} = 185.1229). LACit: *m/z* $[M+H]^+$ = 360.2227 (360.2241); *m/z* y_2 = 247.1392 (247.1401); *m/z* b_2 = 185.1279 (185.1285); *m/z* y_1 = 176.1024 (176.1030); *m/z* z_1 = 159.0757 (159.0764). [$^{13}C_6$, $^{15}N_3$]-LACit: *m/z* $[M+H]^+$ = 369.2350 (369.2359), *m/z* y_2 = 256.1506 (256.1514); *m/z* b_2 = 185.1229 (185.1285); *m/z* z_1 = 167.0905 (167.0907). Observed ions are reported with m/z_{calc} in parentheses.

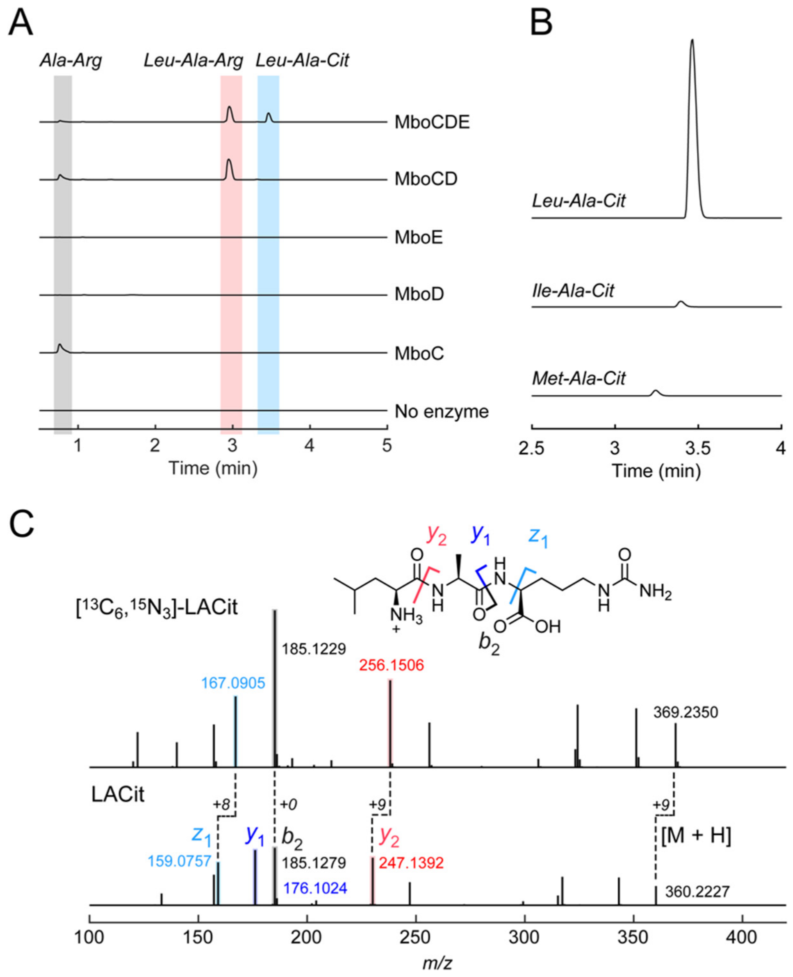

Figure S6. Steady-state kinetic characterization of dipeptide formation by MboC and MboD. MboC and MboD were assayed for amino acid ligation activity of either L-Arg or L-citrulline (0-3 mM) to L-Ala (3.25 mM) by coupling ATP-hydrolysis to NADH-consumption [*31*]. Reactions were initiated with either MboC (1.5 μM) and MboD (1.5 μM). Data points are mean ± s.d. (n = 3). (A) MboC. No appreciable activity above background was observed for Ala-Cit formation with MboC (B) MboD. No appreciable activity above background was observed for either Ala-Arg or Ala-Cit formation. (C) Table of Michaelis-Menten parameters. k_{cat}, K_M, and k_{cat}/K_M determined using a custom MatLab script to simulate the data. k_{cat} and K_M are mean ± s.e., with error in k_{cat}/K_M is propagated from the error of each kinetic term. (n.d. = not detected)

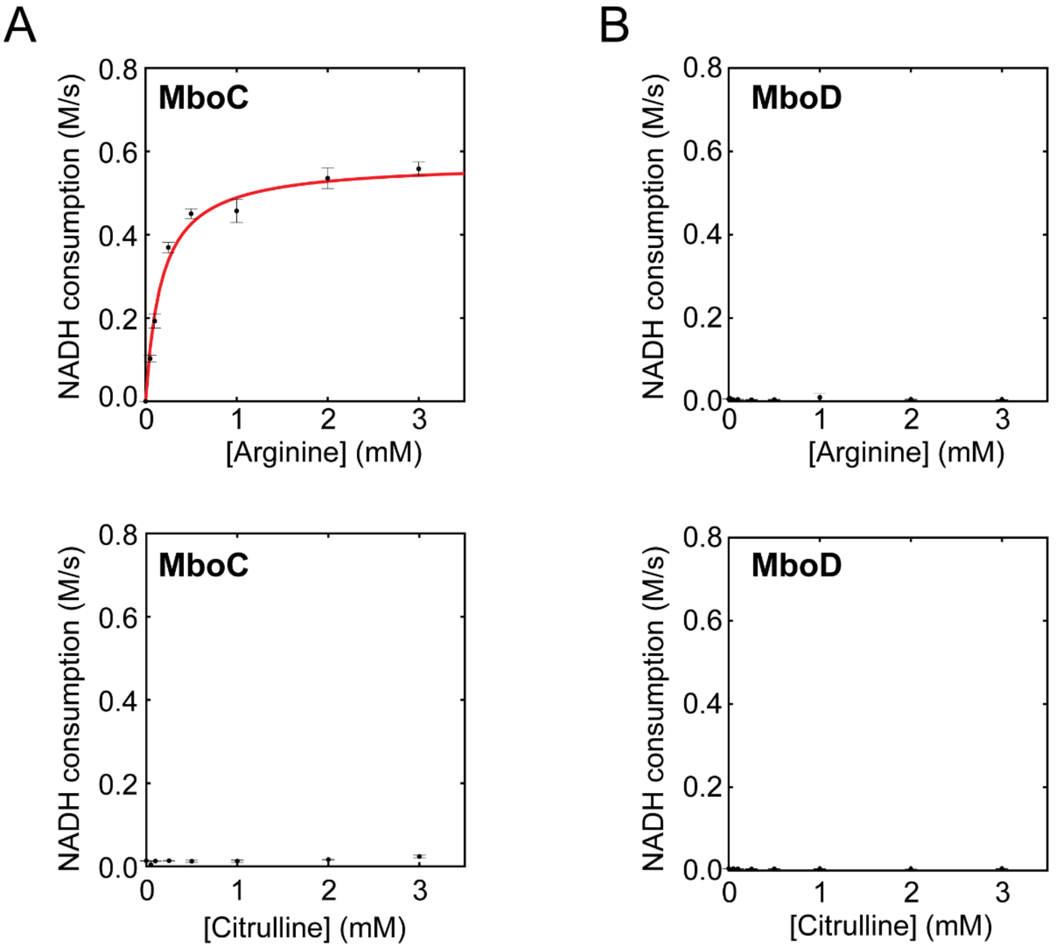

E

Enzyme	Substrate	K_M (μM)	k_{cat} (M s^{-1})	k_{cat}/K_M (M^{-1} s^{-1})
MboC	L-Arg	174 ± 15	0.57 ± 0.1	3300 ± 630
MboC	L-Cit	n.d.	n.d.	n.d.
MboD	L-Arg	n.d.	n.d.	n.d.
MboD	L-Cit	n.d.	n.d.	n.d.

Figure S7. Steady-state kinetic characterization of tripeptide formation by MboD. MboD was assayed for amino acid ligation of either L-Leu (green), L-Ile (black), or L-Met (blue) (0-2 mM) to Ala-Arg (232.5 μM) by coupling ATP-hydrolysis to NADH-consumption [*31*]. Reactions were initiated with MboD (1.5 μM). Data points are mean ± s.d. (n = 3). (B) Table of Michaelis-Menten parameters. k_{cat}, K_M, and k_{cat}/K_M determined using a custom MatLab script to simulate the data. k_{cat} and K_M are mean ± s.e., with error in k_{cat}/K_M is propagated from the error of each kinetic term.

A

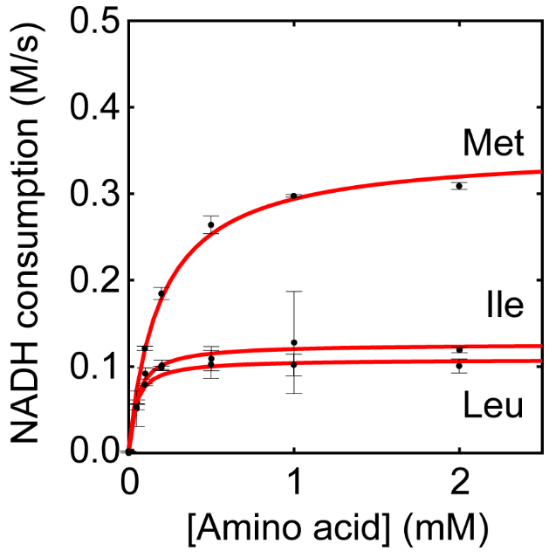

B

Substrate	K_M (μM)	k_{cat} (M s^{-1})	k_{cat}/K_M (M^{-1} s^{-1})
L-Ile	50 ± 15	0.13 ± 0.008	2600 ± 180
L-Leu	42 ± 8	0.11 ± 0.004	2620 ± 110
L-Met	200 ± 16	0.35 ± 0.008	1750 ± 40

Figure S8. 1D- and 2D-NMR characterization of Ala-Arg substrate synthesized by solid-phase peptide synthesis. (A) ^{1}H 1D-NMR spectrum of Ala-Arg in D_2O. (B) ^{1}H–^{13}C HSQC spectrum of Ala-Arg in D_2O. (C) ^{1}H-^{13}C HMBC spectrum of Ala-Arg in D_2O. (D) ^{1}H and ^{13}C NMR Shifts for Ala-Arg. NMR shifts (δ) for Ala-Arg where d = doublet, t = triplet, q = quartet, dd = doublet of doublets, and m = multiplet.

A

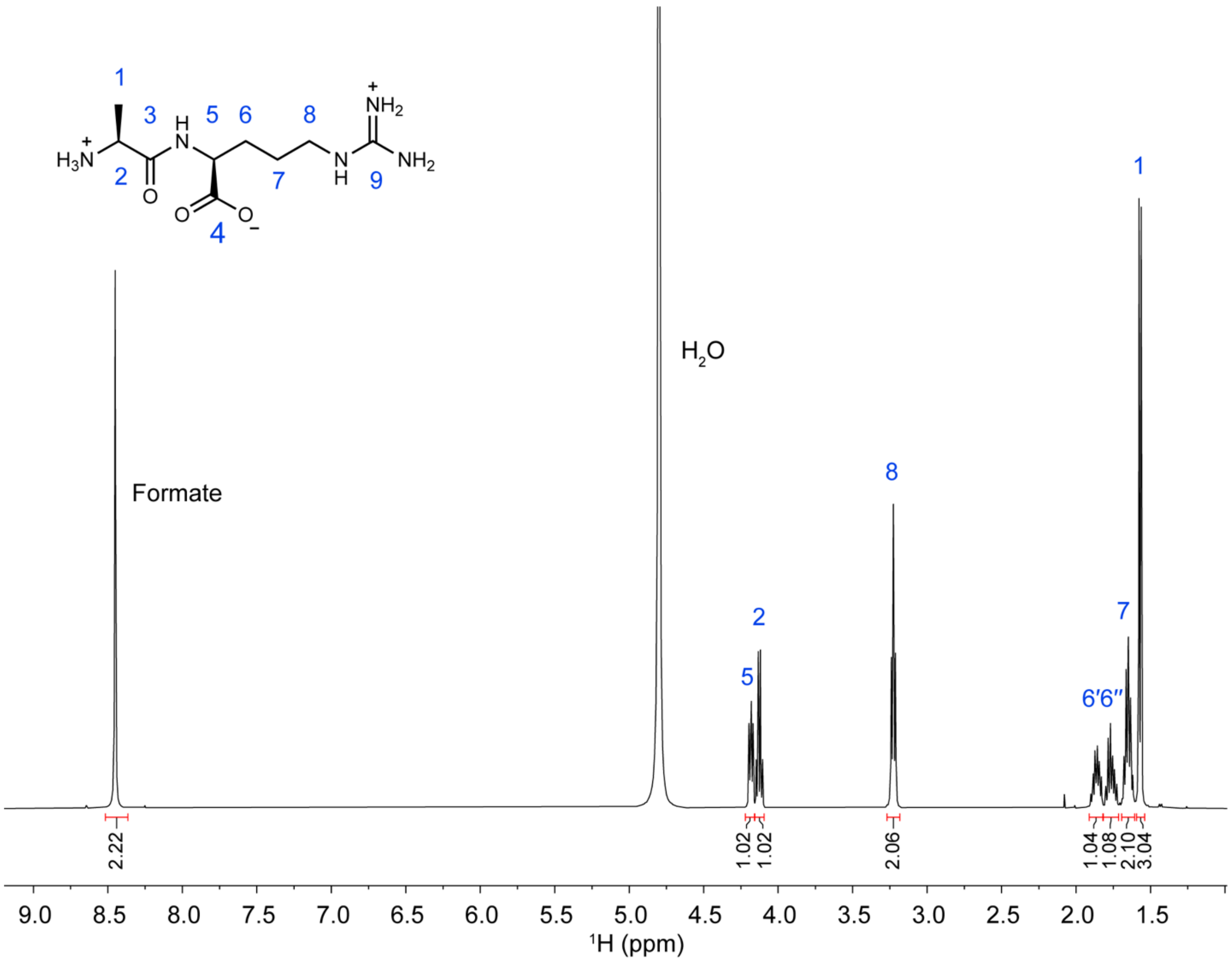

B

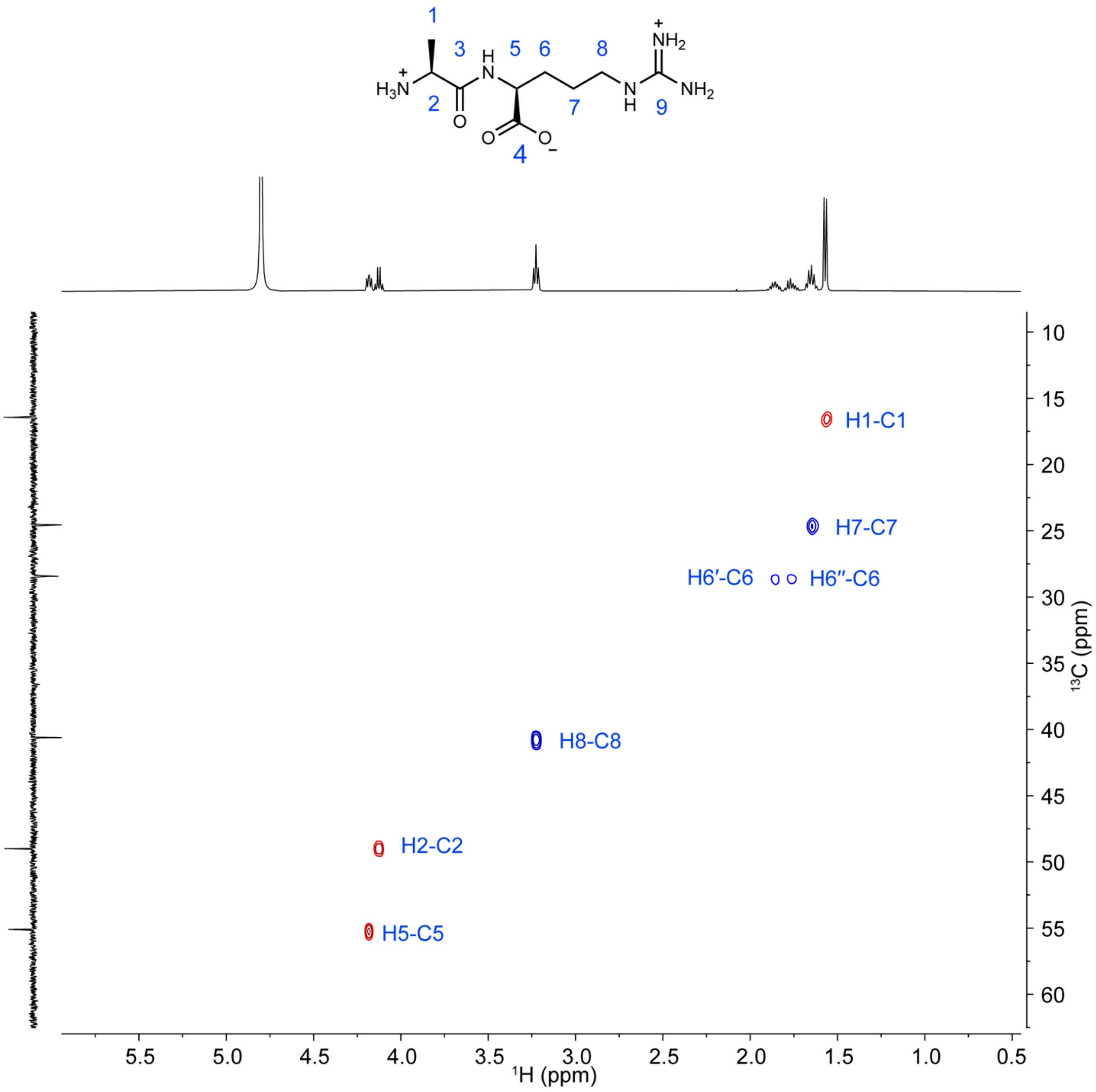

C

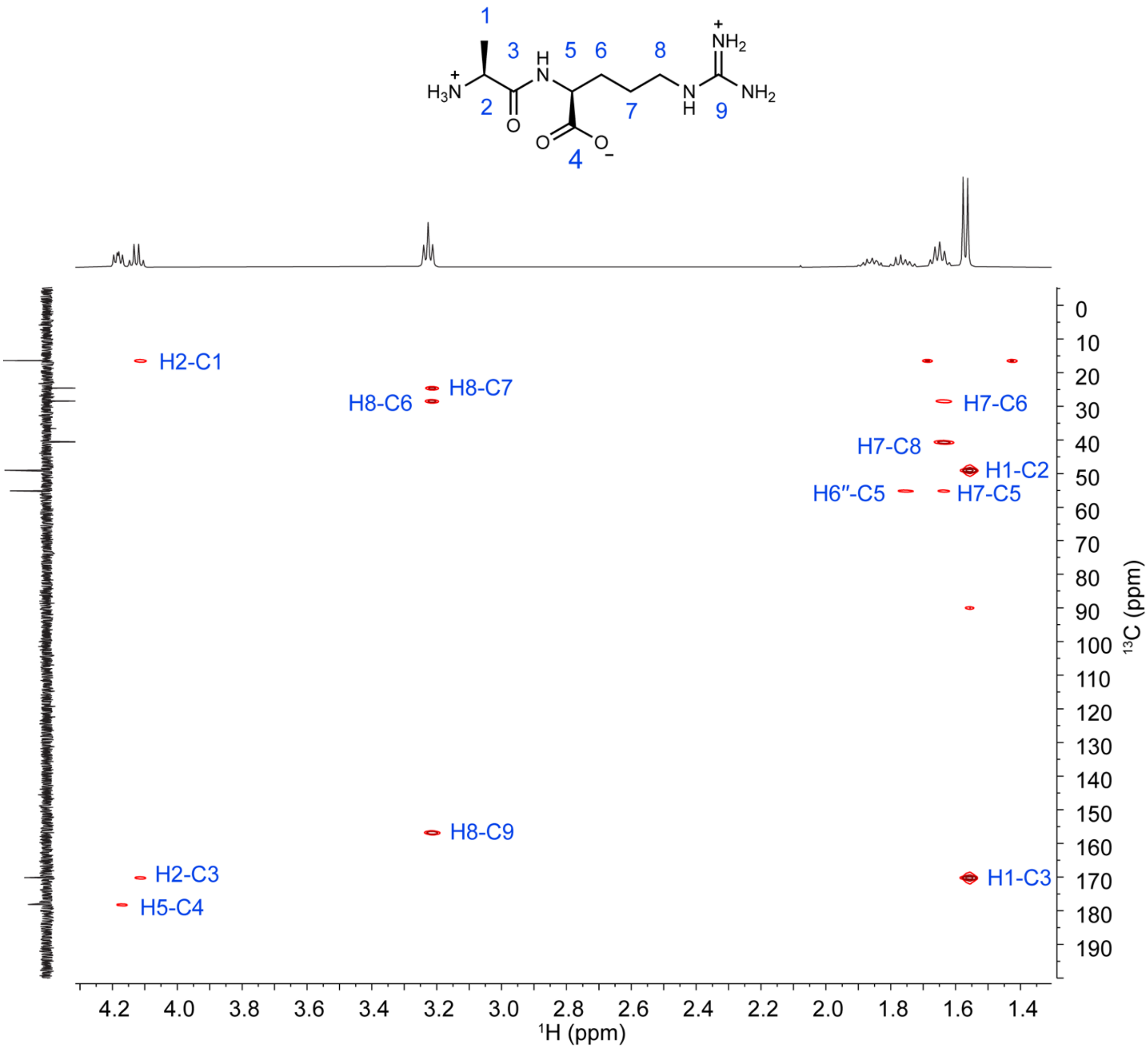
H2-C1
H8-C7
H8-C6
H7-C6
H7-C8
H1-C2
H6″-C5
H7-C5
H8-C9
H2-C3
H1-C3
H5-C4
^{1}H (ppm)
^{13}C (ppm)

D

Atom	Chemical shift (ppm)
H1	1.57 (d)
H2	4.12 (q)
H5	4.17 (dd)
H6’	1.86 (m)
H6’’	1.76 (m)
H7	1.65 (m)
H8	3.22 (t)
C1	16.5
C2	49.2
C3	170.3
C4	178.8
C5	55.4
C6	28.7
C7	24.7
C8	40.8
C9	157.7

Figure S9. Comparison of MboB to a related reductase (GcoB). GcoB is a reductase that serves as a redox partner to a P450 and has been experimentally and structurally characterized [*32*]. These iron-sulfur (FeS) flavoproteins utilize three domains (FAD/FMN, NAD(P)H, and FeS) to mediate one-electron transfer from NAD(P)H to the target protein, such examples include P450s [*32*], Rieske non-heme Fe dioxygenases [*33-35*], and ferritin-like dioxygenases [*36, 37*]. This family of flavoprotein reductases are distinct as they feature a fused ferredoxin-NADPH reductase and ferredoxin domains (N-terminal) into one polypeptide. Highlighted (in green) are the ligands for the Fe_2S_2 cluster in the N-terminal ferredoxin domain of MboB and GcoB.

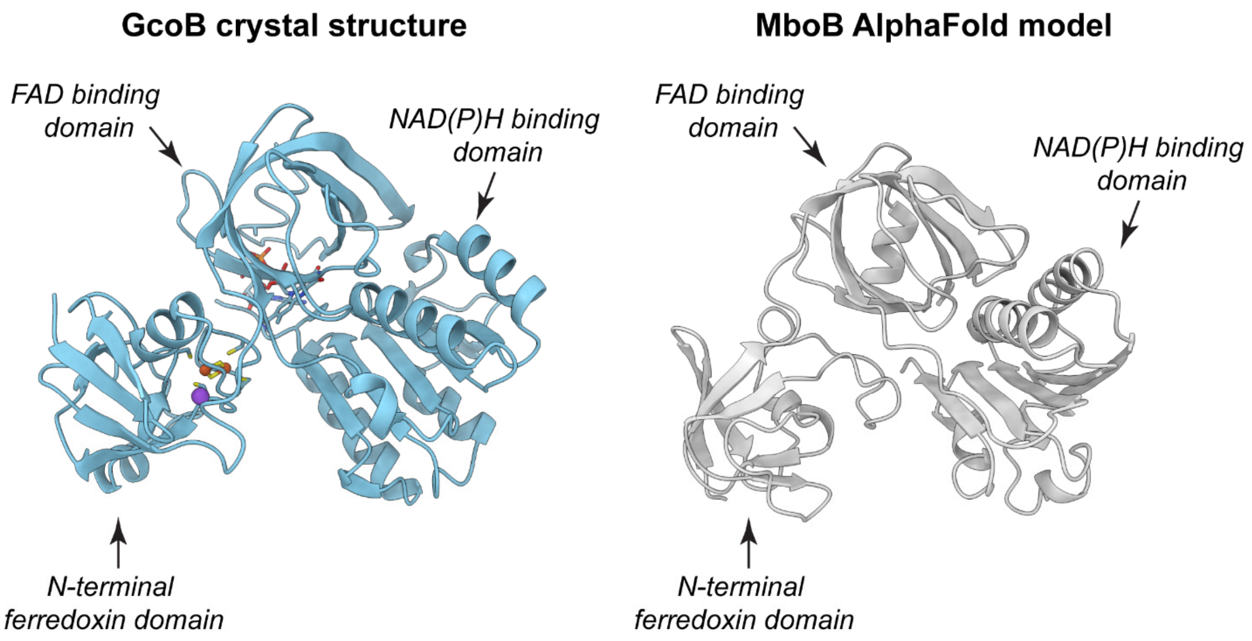

MboB C L K G V C R V C K C T L V S G R V L E H G K A V A L - - - - - - - K D T F L P C
GcoB C N Q G T C G T C K L Q V L S G E V D H G G A P E D T L S A E E R A S G L A L A C

Figure S10. Characterization of *in vitro* MboABCDE reconstitution. (A) MboABCDE was reconstituted using L-Ala, L-Arg, and L-Leu (1 mM each) in the presence of ATP (5 mM), Fe(II) (100 µM), NADH (2.5 mM), $MgCl_2$ (5 mM), and ascorbate (1 mM). The following ions were extracted to monitor different products: Ala-Arg (**1**, *m/z* = 246.1569), Leu-Ala-Arg (**2**, *m/z* = 359.2414), Leu-Ala-Arg_{alkyne} (**3**, *m/z* = 355.2114), Leu-Ala-Cit_{alkyne} (**4**, *m/z* = 356.1954) and Leu-Ala-Cit (**5**, *m/z* = 360.2267). (*, cyclized product of **3**). (B) EICs monitoring order of addition studies of MboAB compared to MboE using the Leu-Ala-Arg tripeptide substrate (0.25 mM). This experiment indicates that Leu-Ala-Cit does not appear to be accepted by MboAB, indicating that the MboE-catalyzed step occurs after alkyne formation. EICs are the sum of the extracted ions for Leu-Ala-Cit (*m/z* = 360.2262) and Leu-Ala-Cit_{alkyne} (*m/z* = 356.1943). (C) Assessment of necessary components are required for alkyne formation from Leu-Ala-Arg by MboAB. Alkyne formation is only observed when MboA, MboB, NADH and Fe^{2+} are all present. Omission of MboB and NADH results in formation of Leu-Ala-Arg_{alkene}, indicating that alkyne formation stalls in their absence. The redox equivalents provided by MboB cannot be substituted by ascorbic acid or an electron mediator like phenazine methosulfate (PMS) as observed in other HDOs [*2*]. Omission of Fe^{2+} results in only trace amounts of activity, suggesting that MboA may be isolated with a low level of mature cofactor. EICs represent the extracted ion for Leu-Ala-Cit_{alkyne} (*m/z* = 356.1929) or Leu-Ala-Arg_{alkene} (*m/z* = 357.2229).

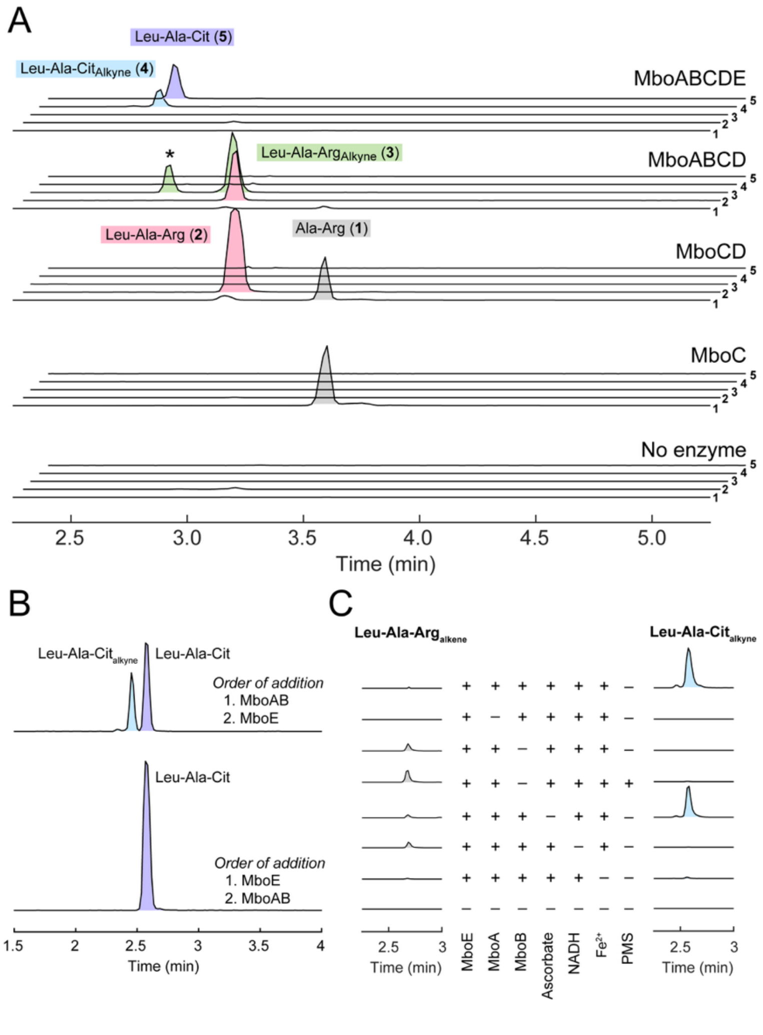

Figure S11. 1D- and 2D-NMR characterization of Leu-Ala-Cit$_{alkyne}$. Leu-Ala-Cit$_{alkyne}$ was prepared from the *in vitro* reconstitution of MboABE from Leu–Ala-Arg (1 mM) with NADH (5 mM) Fe(II) (100 μM), and ascorbate (1 mM) and purified by HILIC chromatography. (A) Summary of key ^{1}H-^{1}H COSY and ^{1}H-^{13}C HMBC correlations for Leu-Ala-Cit$_{alkyne}$. The correlations highlighted in red support the presence of an internal alkyne in the MboABCDE *in vitro* product. In the ^{1}H-^{13}C HMBC spectrum (Panel C), H13 (H$_δ$) was first assigned by its correlation to the urea carbon [3.83 ppm, 161.12 ppm]. Through this assignment, we identified the position of the internal alkyne to be between C11 (C$_β$) and C12 (C$_γ$), via two critical correlations with H13 (H$_δ$) that are diagnostic of an internal alkyne [3.83 ppm, 79.90 ppm] and [3.83 ppm, 77.97 ppm]. In the ^{1}H-^{1}H COSY spectrum (Panel D), H13 (C$_δ$) exhibited a cross peak with one other proton that had a moderate downfield shift [3.83 ppm, 4.80 ppm]. This proton was assigned to H10 (H$_α$) as it was the only remaining proton in the Cit residue. Moreover, the ^{1}H-^{13}C HSQC cross peak [4.80, 46.82] indicates that it is attached to a methine carbon with a chemical shift typical for C$_α$'s. This coupling is notably at a greater distance (5-bond, J_5) than what can be usually observed in a COSY experiment. However, it has been established that the presence of unsaturated motifs (*e.g.* alkynes) can facilitate long-range interactions [*38, 39*]. Thus, the observation of the [3.83 ppm, 4.80 ppm] cross peak provides additional support for the presence of an alkyne. (B) ^{1}H NMR spectrum of Leu-Ala-Cit$_{alkyne}$ in D_2O. The aliphatic region lacks peaks that would be expected if the structure contained aliphatic cyclic moieties. Instead, the ^{1}H NMR spectrum is consistent with a linear structure exhibiting two degrees of unsaturation. (C) ^{1}H-^{13}C HMBC NMR spectrum of Leu-Ala-Cit$_{alkyne}$ in D_2O. Cross peaks between H13 (H$_δ$) and C14 (urea carbonyl) allow assignment of H13. Cross peaks between H13 (H $_δ$) and C12 (C $_γ$)/C11 (C$_β$) allow assignment of the alkyne carbons. (D) ^{1}H-^{1}H COSY NMR spectrum of Leu-Ala-Cit$_{alkyne}$ in D_2O. Notably, peaks for H11 (C$_β$) and H12 (C$_γ$) are absent but a pair of cross peaks between H10 (C$_α$) and H13 (C$_δ$) are present. The lower intensity of these cross peaks is consistent with the 3-bond separation. (E) ^{1}H-^{13}C HSQC NMR spectrum of Leu-Ala-Cit$_{alkyne}$ in D_2O. Given the diagnostic ^{13}C chemical shifts for alkenes (100-140 ppm) and allenes (~80, 100, and 200 ppm), the HSQC experiment should be able to distinguish between these motifs compared to an alkyne. Indeed, the HSQC spectrum lacks any of the expected correlations between with an alkene or allene carbon. The lack of any further observable ^{1}H-^{13}C signals in this experiment is consistent with the presence of the two quaternary carbons from the internal alkyne. (F) Chemical shifts (δ) of each atom for Leu-Ala-Cit$_{alkyne}$ where d = doublet, t = triplet, q = quartet, dd = doublet of doublets, and m = multiplet. Note that our NMR data supports the isolation of a single diastereomer, however we cannot rule out that desaturation is concomitant with epimerization. As a result, we cannot assign the absolute stereochemistry of the modified Cit.

A

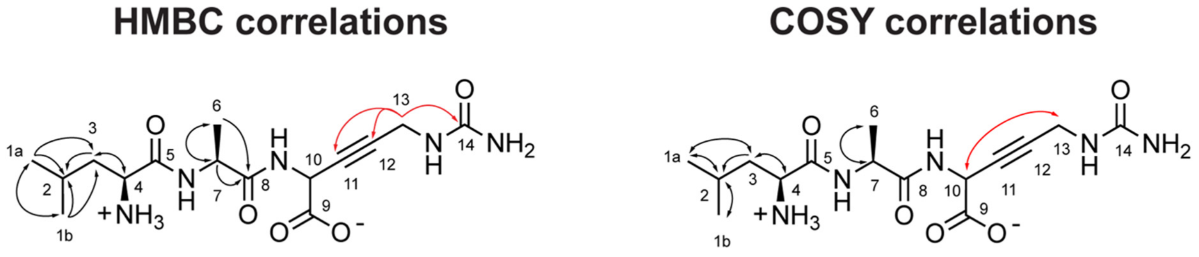

B

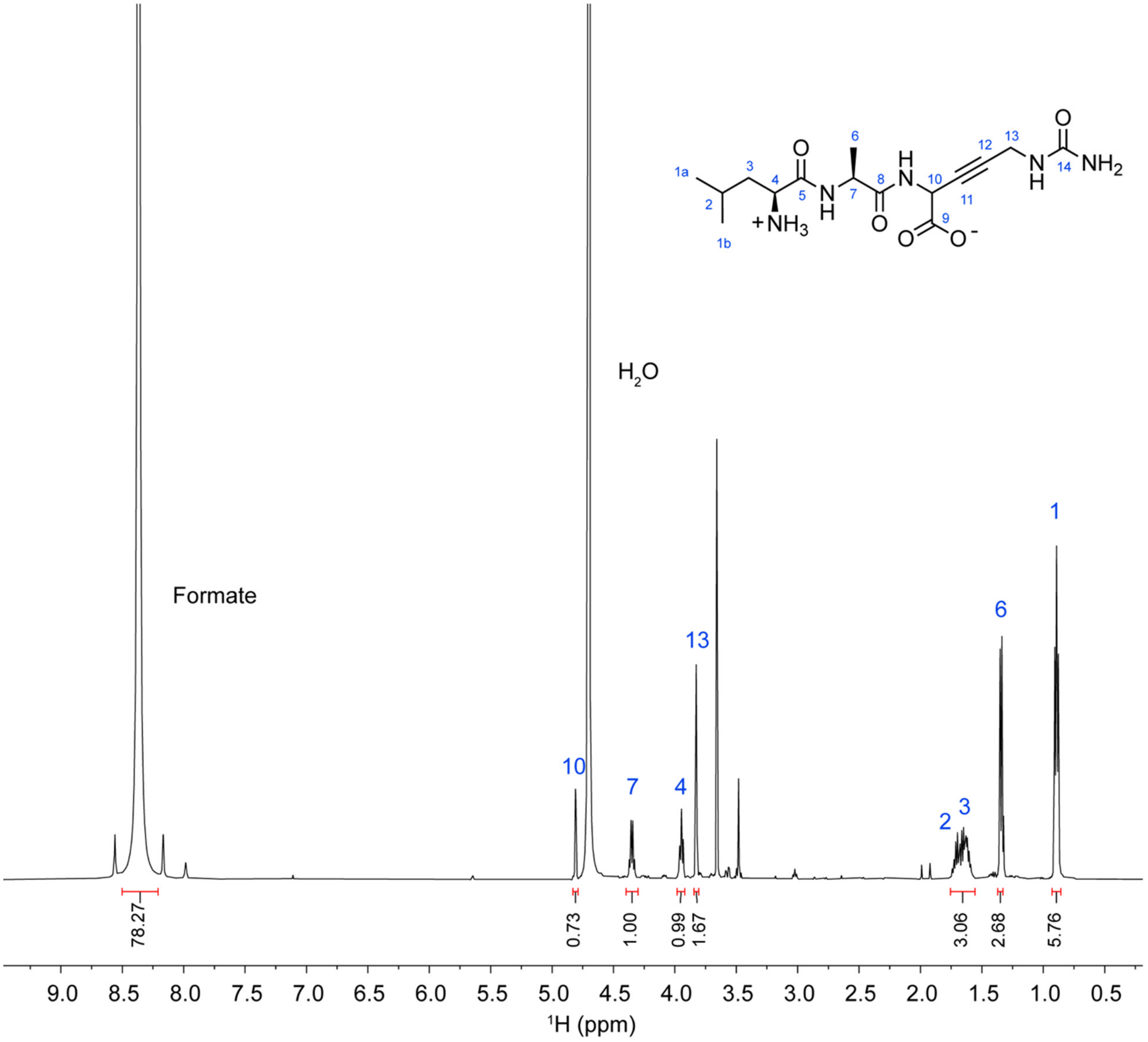
H2O
Formate
1
6
13
10
7
4
2
3
78.27
0.73
1.00
0.99
1.67
3.06
2.68
5.76
9.0 8.5 8.0 7.5 7.0 6.5 6.0 5.5 5.0 4.5 4.0 3.5 3.0 2.5 2.0 1.5 1.0 0.5
1H (ppm)

C

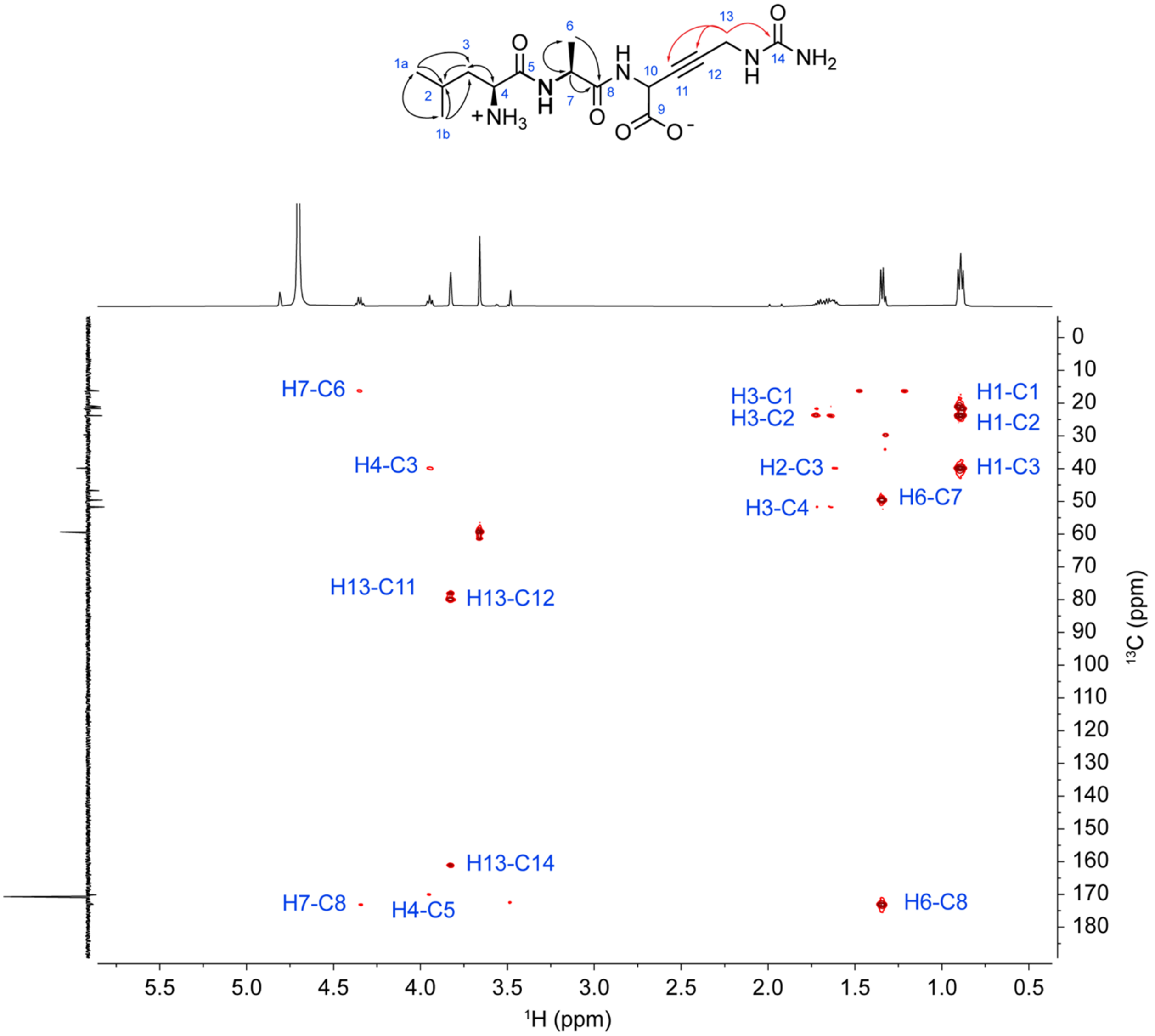

1a
2
1b
3
4
+NH3
5
6
7
8
9
10
11
12
13
14
NH2
H7-C6
H3-C1
H3-C2
H1-C1
H1-C2
H4-C3
H2-C3
H1-C3
H6-C7
H3-C4
H13-C11
H13-C12
H13-C14
H7-C8
H4-C5
H6-C8
^{13}C (ppm)
^{1}H (ppm)

D

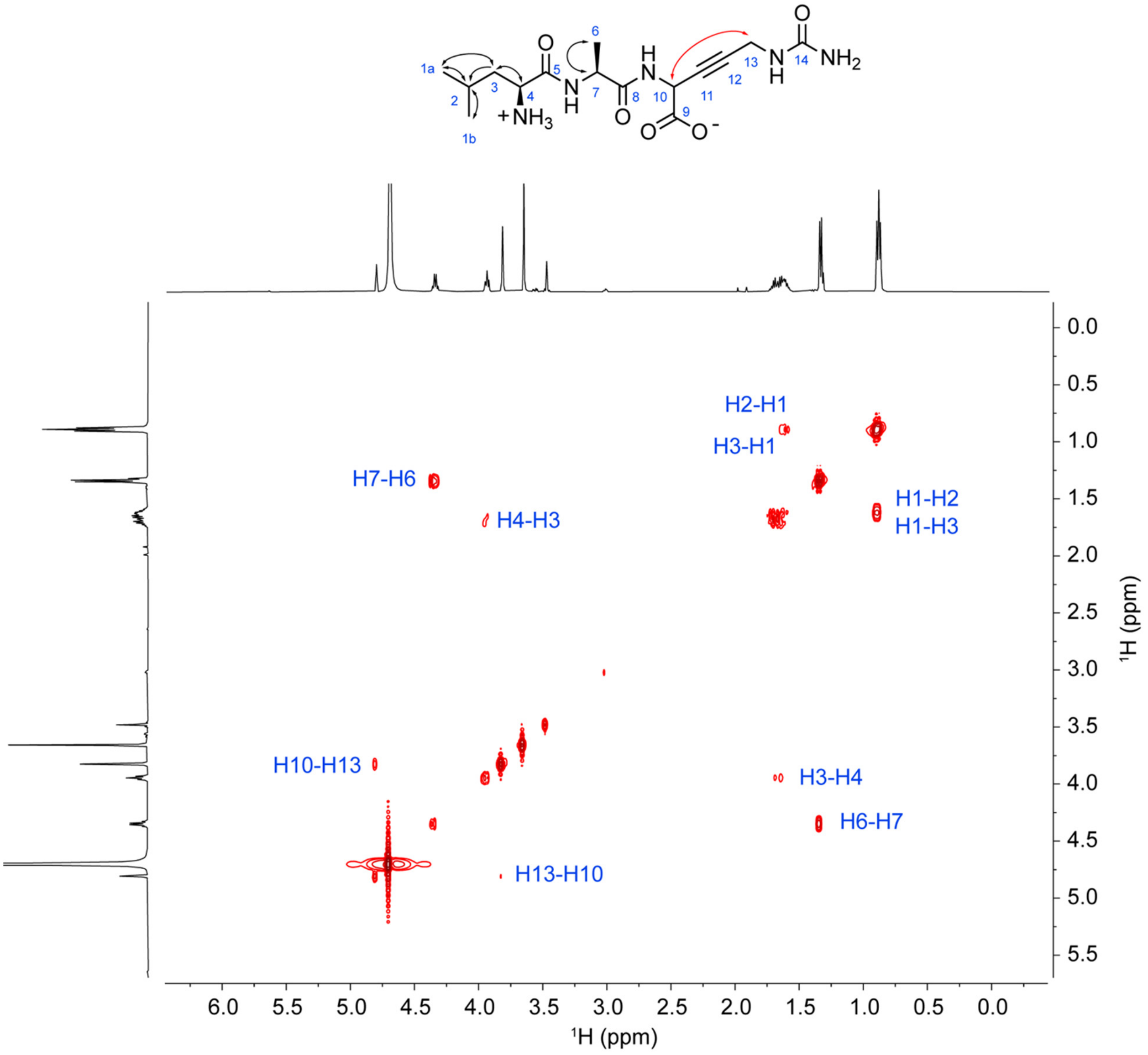
1a
2
1b
3
4
+NH3
5
O
N
H
6
7
8
O
H
N
10
9
O
O-
11
12
13
N
H
14
O
NH2
H2-H1
H3-H1
H7-H6
H4-H3
H1-H2
H1-H3
H10-H13
H3-H4
H6-H7
H13-H10
6.0
5.5
5.0
4.5
4.0
3.5
3.0
2.5
2.0
1.5
1.0
0.5
0.0
5.5
¹H (ppm)
¹H (ppm)

E

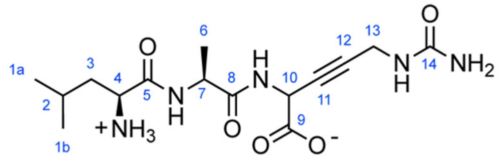

1a
2
1b
3
4
+NH3
5
O
N
H
6
7
8
O
H
N
10
9
O
O-
12
11
13
N
H
14
O
NH2

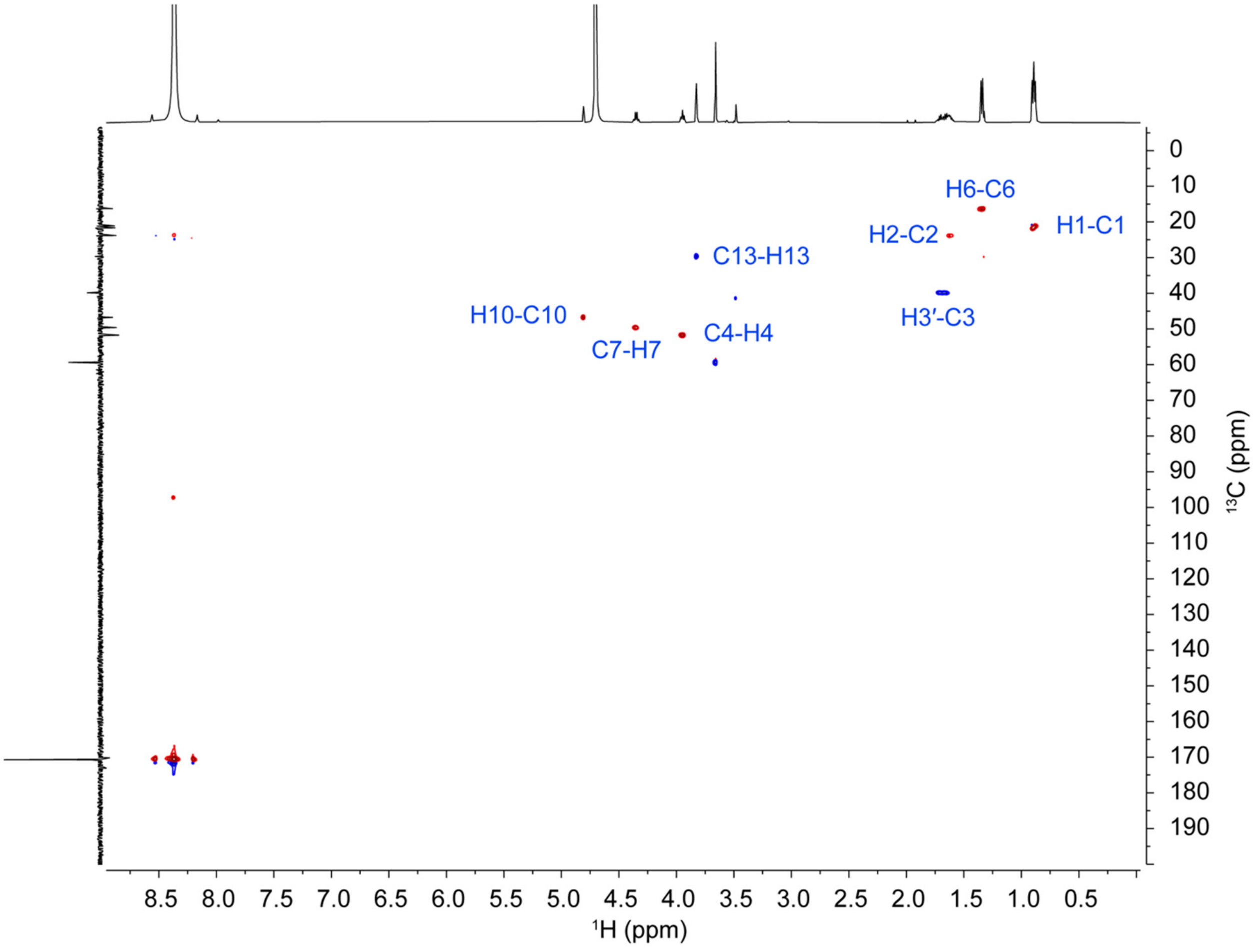

H6-C6
H2-C2
H1-C1
C13-H13
H3'-C3
H10-C10
C7-H7
C4-H4
0
10
20
30
40
50
60
70
80
90
100
110
120
130
140
150
160
170
180
190
13C (ppm)
8.5
8.0
7.5
7.0
6.5
6.0
5.5
5.0
4.5
4.0
3.5
3.0
2.5
2.0
1.5
1.0
0.5
1H (ppm)

F

Atom	NMR Shift (ppm)
H1[a,b]	0.88,0.89 (d)
H2	1.63 (m)
H3	1.66, 1.71 (m,m)
H4	4.36 (q)
H6	1.34 (d)
H7	3.95 (t)
H10	4.80 (t)
H13	3.83 (m)
C1	21.03, 21.46
C2	23.89
C3	39.94
C4	51.72
C5	170.14
C6	16.31
C7	49.59
C8	173.15
C9	172.72.
C10	46.82
C11	77.97
C12	79.90
C13	29.63
C14	161.12

Figure S12. *E. coli* growth inhibition assays with Leu-Ala-Cit and Leu-Ala-Cit$_{alkyne}$. Assessing the antibacterial properties of Leu-Ala-Cit and Leu-Ala-Cit$_{alkyne}$ via an *E. coli* growth inhibition assay. Plates were incubated for 1 d of growth. (A) Plate with 50 µM Leu-Ala-Cit (10 µL) added. (B) Plate supplemented with 1 mM Orn with 50 µM Leu-Ala-Cit (10 µL) added. (C) Plate with 50 µM Leu-Ala-Cit$_{alkyne}$ (10 µL) added. Diameter of growth inhibition regions: 1.04, 1.10, and 1.06 cm (D) Plate supplemented with 1 mM Orn with 50 µM µM Leu-Ala-Cit$_{alkyne}$ (10 µL). Diameter of growth inhibition regions: 0.89, 0.88, and 0.88 cm. Note that the regions of inhibited growth are only present when Leu-Ala-Cit$_{alkyne}$ is added. Moreover, these regions are less intense when 1 mM Orn is supplemented into the agar media, consistent with previous studies of mangotoxin [*20, 21*].

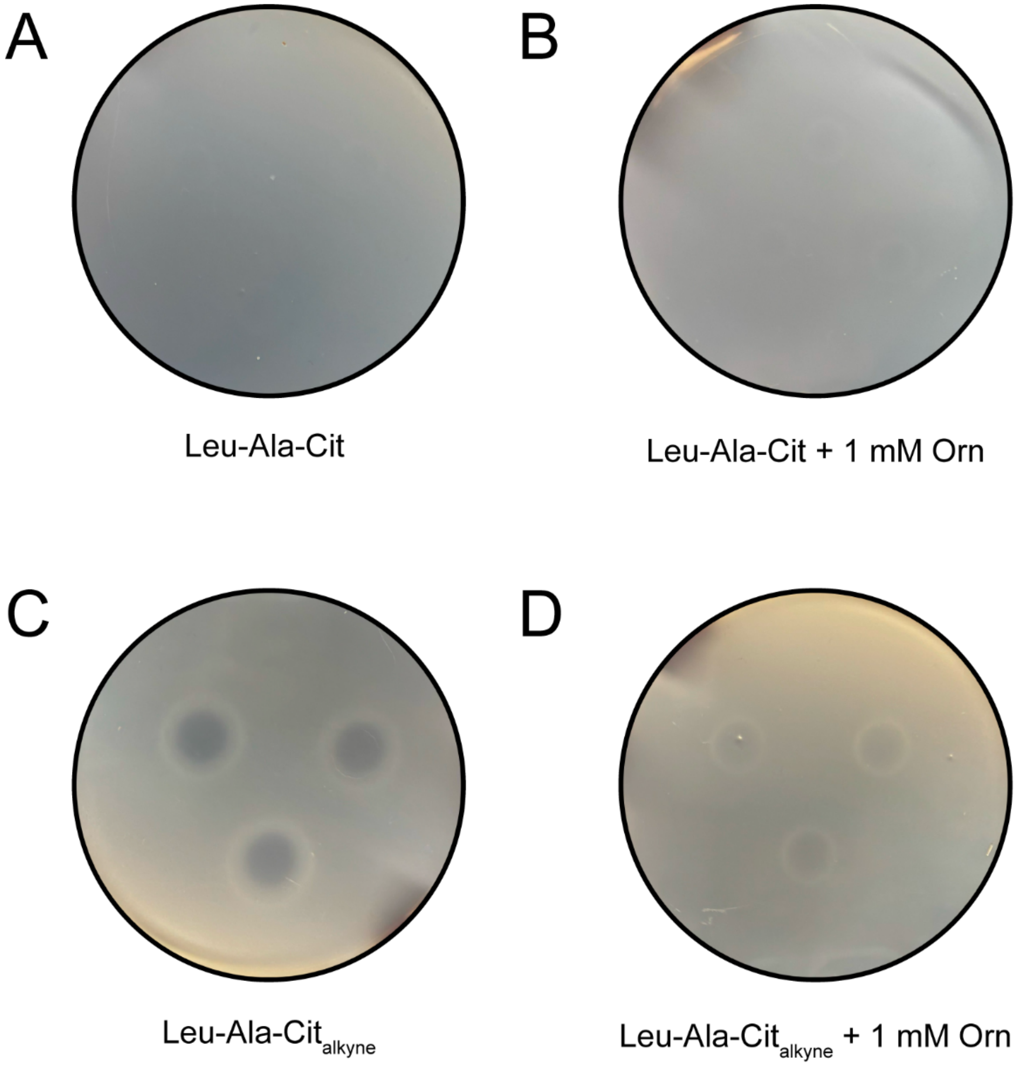

Figure S13. LC-MS/MS characterization of alkyne tripeptide cyclization in H_2O and D_2O. The formation of the side-product of the alkyne tripeptides from reactions with MboAB (Leu-Ala-Arg_{alkyne}) or MboABE (Leu-Ala-Cit_{alkyne}) was characterized by varying quenching conditions. (A) EICs following Leu-Ala-Arg_{alkyne} (*m/z* 355.2089) with different exposures to acidic conditions (FA, formic acid). (B) Extracted mass spectra for peak 1 and 2 from LC-QTOF/MS analysis of *in vitro* reconstitution of Leu-Ala-Cit_{alkyne} production with MboABE in H_2O or D_2O quenched with acidic MeCN and incubated for 120 min (m/z_{calc} $[M+H]^+$ = 355.2089, m/z_{calc} $[M+1+H]^+$ = 356.1929). (C) Extracted mass spectra for peak 1 and 2 from LC-QTOF/MS analysis of *in vitro* reconstitution of Leu-Ala-Cit_{alkyne} production with MboABE in H_2O or D_2O quenched with acidic MeCN and incubated overnight (m/z_{calc} $[M+H]^+$ = 356.2152, m/z_{calc} $[M+1+H]^+$ = 357.1992).

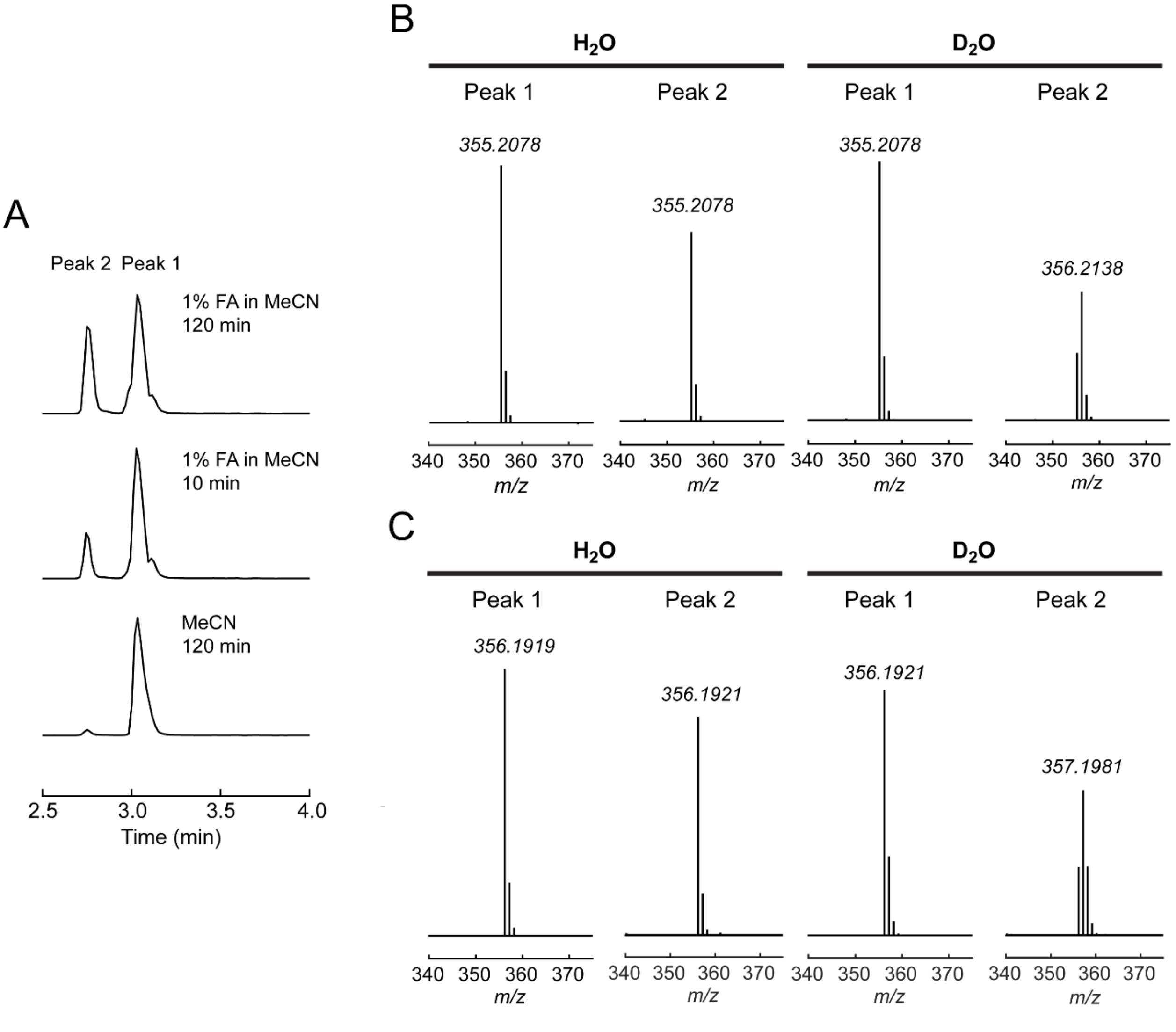

Figure S14. 1D- and 2D-NMR characterization of cyclized Leu-Ala-Cit$_{alkyne}$. The side-product of Leu-Ala-Cit$_{alkyne}$ formation was prepared from the *in vitro* reconstitution of MboABE from Leu-Ala-Arg (1 mM) with NADH (5 mM), Fe(II) (100 μM), and ascorbate (1 mM). The reaction was quenched in 1% (*v/v*) formic acid in MeCN and incubated overnight to increase the yield of cyclized tripeptide and purified by two rounds of HILIC chromatography. (A) Summary of key ^{1}H-^{13}C HMBC and ^{1}H-^{1}H COSY correlations for the cyclized Leu-Ala-Cit$_{alkyne}$. The correlations highlighted in red support the assigned heterocycle connectivity. Comparison of the ^{1}H 1D-NMR spectrum of Leu-Ala-Cit$_{alkyne}$ and its side-product suggest that an intramolecular cyclization involving C$_{\alpha}$ of the Cit$_{alkyne}$ residue has taken place resulting in two new downfield protons observed at δ 7.37 ppm and 5.22 ppm (Panel B). The ^{1}H-^{13}C HSQC [7.37, 130.88] (Panel C) and ^{1}H-^{13}C HMBC [7.37, 170.62] (Panel D) cross peaks indicate that the new proton at δ 7.37 ppm is a vinyl proton near the C-terminal carboxylate. As no second vinyl proton was observed, the double bond location is assigned to C10 (C$_{\alpha}$) and C11 (C$_{\beta}$) rather than C11 (C$_{\beta}$) and C12 (C$_{\gamma}$), where the alkyne was originally located in Leu-Ala-Cit$_{alkyne}$. The new proton at δ 5.22 ppm was assigned to a methine proton at C12 (C$_{\gamma}$) based on its ^{1}H-^{13}C HSQC phase and the observed ^{1}H-^{1}H COSY cross peaks between H12 (H$_{\gamma}$) and H11 (C$_{\beta}$) [7.37 ppm, 5.22 ppm] as well as between H12 (H$_{\gamma}$) and the two H13s (H$_{\delta}$) [5.22 ppm, 3.40 ppm]/[5.22 ppm, 3.61 ppm] (Panel E). Based on the downfield chemical shift of C12 (C$_{\gamma}$, 81.76 ppm) and its attached proton H12 (H$_{\gamma}$, 5.22 ppm), it should participate in either a C–N or C–O bond, consistent with cyclization. Cyclization at this moiety could occur with either the urea group or the C-terminal carboxylate. At this time, we favor cyclization with the C-terminal carboxylate as this results in chemical shifts that show better agreement with the predicted NMR spectrum as cyclization through the urea would result in a less downfield-shifted proton (~4-5 ppm) (Panel H) [*39*]. (B) Comparison of the ^{1}H 1D-NMR spectrum of Leu-Ala-Cit$_{alkyne}$ and the cyclized product in D_2O. Notably, H10 (H$_{\alpha}$) of the Cit$_{alkyne}$ residue has disappeared in the spectrum of the cyclized product and two new protons appear downstream. (C) ^{1}H-^{13}C HSQC NMR spectrum in D_2O. (D) ^{1}H-^{13}C HMBC NMR spectrum of Leu-Ala-Cit$_{alkyne}$ in D_2O after only a single round of purification with residual impurities at ~3.5 ppm. This higher concentration sample allows lower intensity crosspeaks to be visualized. (E) ^{1}H-^{13}C HMBC NMR spectrum of Leu-Ala-Cit$_{alkyne}$ in D_2O. (F) ^{1}H-^{1}H COSY NMR spectrum in D_2O. (G) Chemical shifts for each atom for Leu-Ala-Cit$_{alkyne}$ where d = doublet, t = triplet, q = quartet, dd = doublet of doublets, and m = multiplet. (H) Comparison of the predicted ^{1}H 1D-NMR spectra for various cyclized products of Leu-Ala-Cit$_{alkyne}$ (ChemDraw Professional 23.1.1.3).

A

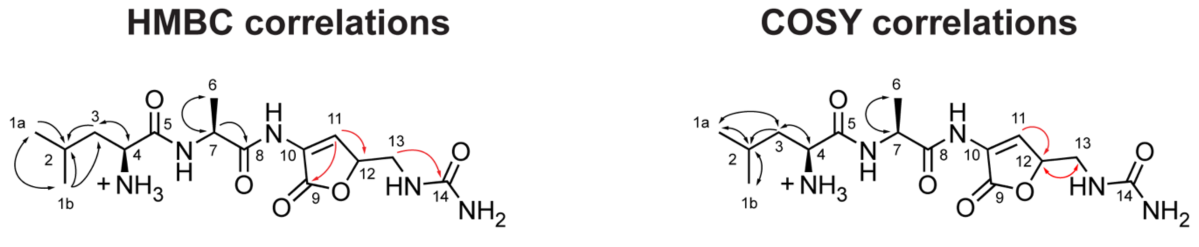

B

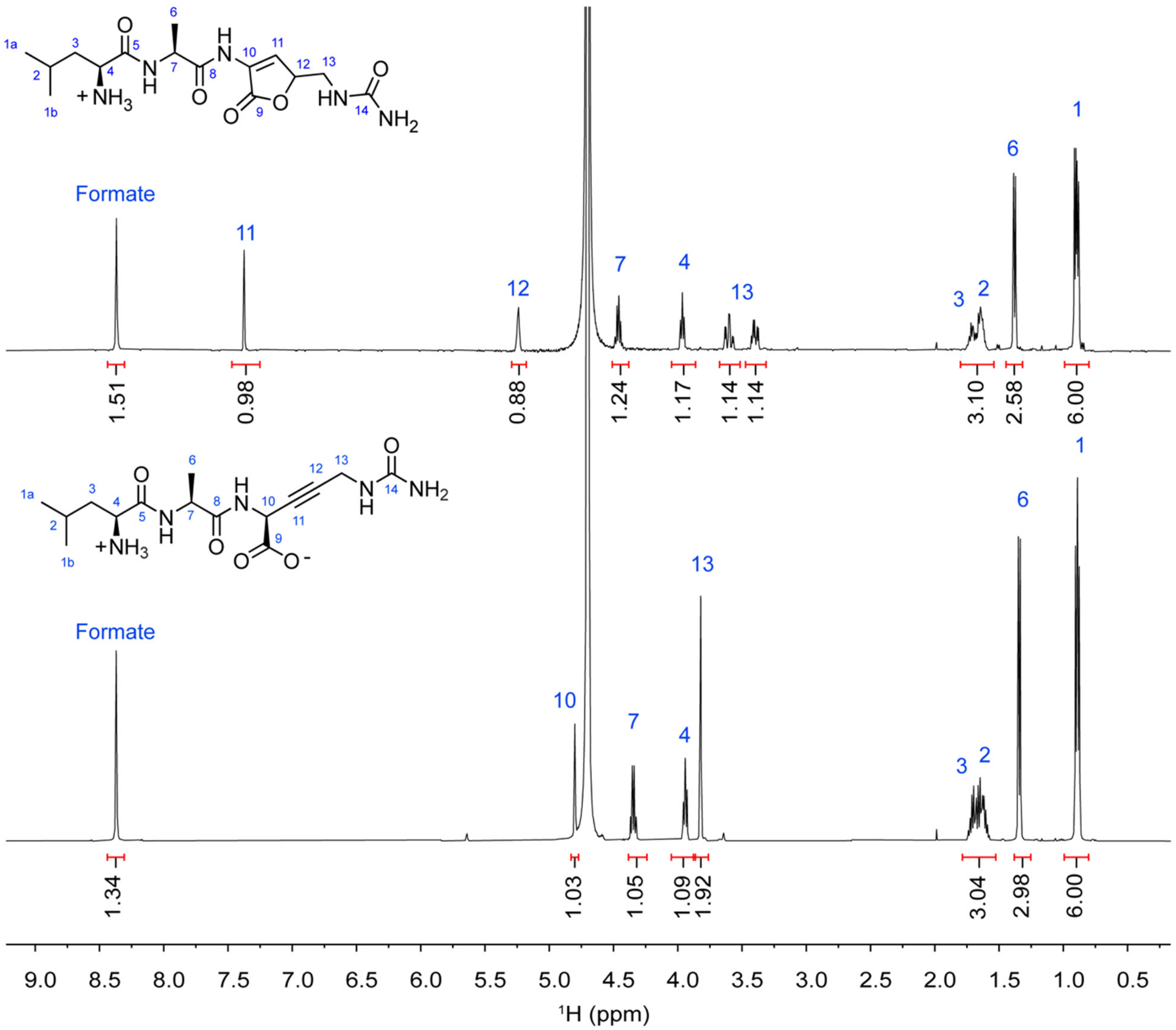

Formate
11
12
7
4
13
3
2
6
1
1.51
0.98
0.88
1.24
1.17
1.14
1.14
3.10
2.58
6.00
Formate
10
7
4
13
3
2
6
1
1.34
1.03
1.05
1.09
1.92
3.04
2.98
6.00
9.0 8.5 8.0 7.5 7.0 6.5 6.0 5.5 5.0 4.5 4.0 3.5 3.0 2.5 2.0 1.5 1.0 0.5
1H (ppm)

C

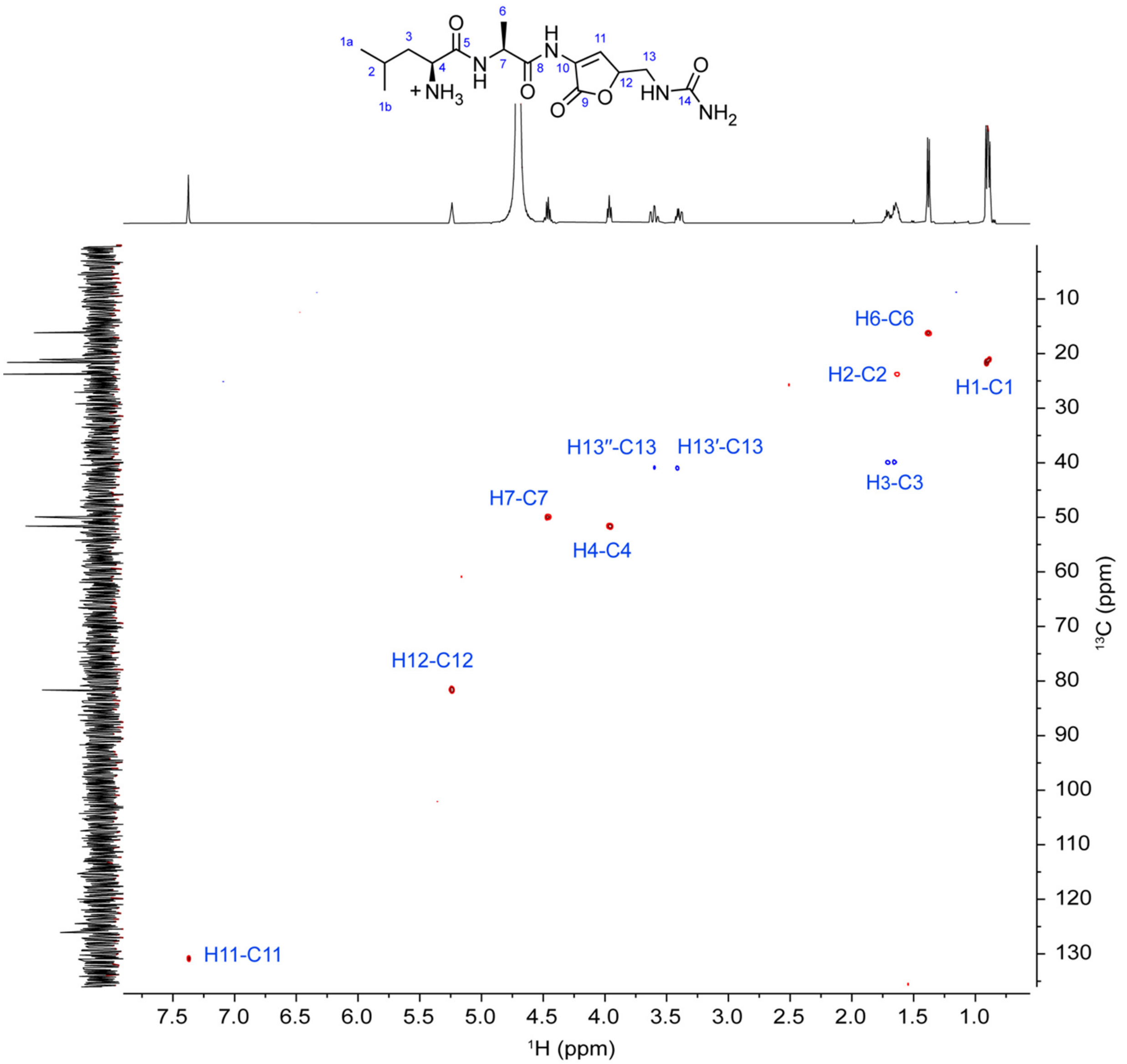

1a
2
1b
3
4
+NH3
5
H
N
6
7
8
10
11
9
12
13
14
O
HN
NH2
H6-C6
H2-C2
H1-C1
H13″-C13
H13′-C13
H3-C3
H7-C7
H4-C4
H12-C12
H11-C11
13C (ppm)
1H (ppm)

D

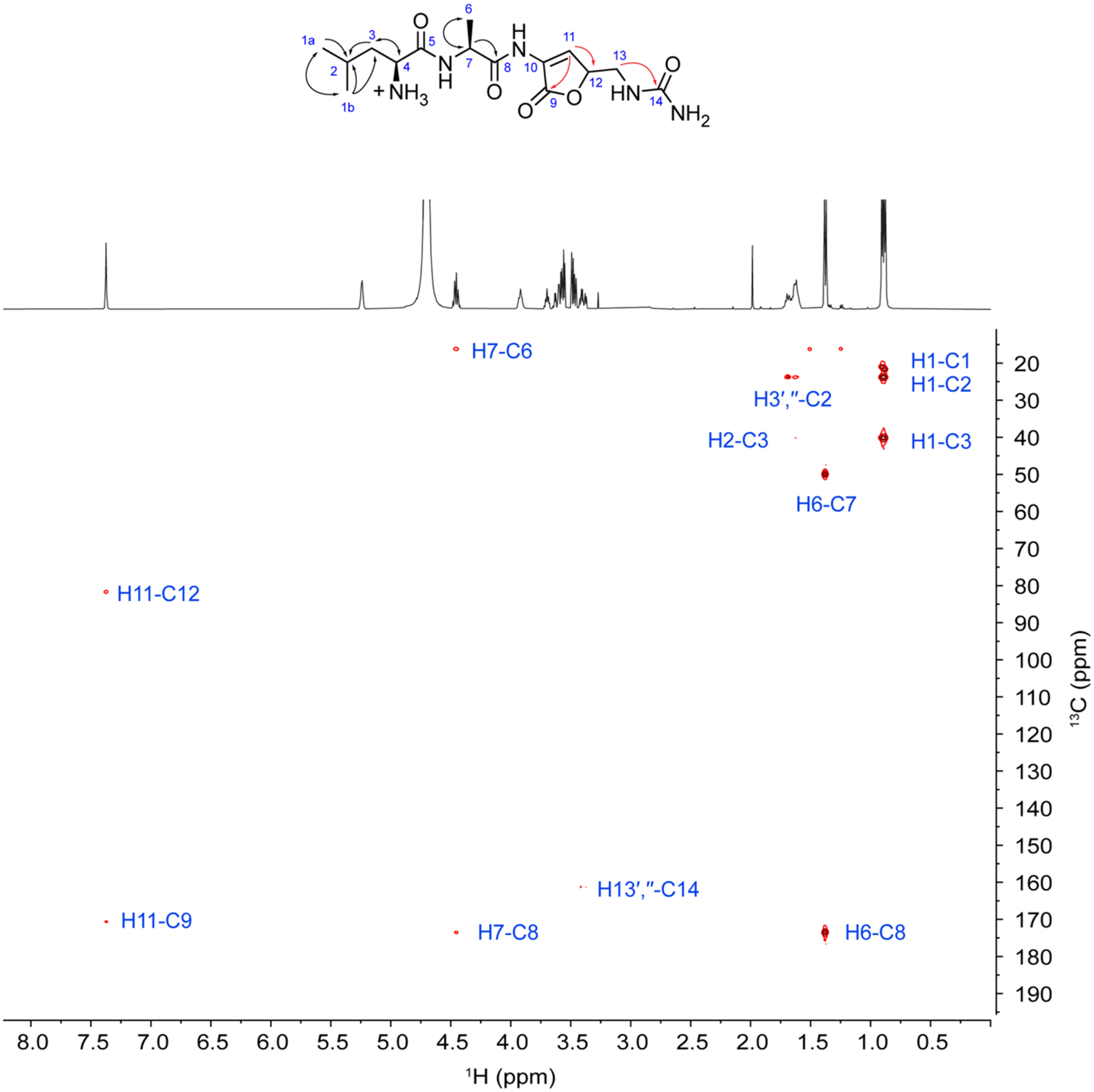
H7-C6
H1-C1
H1-C2
H3′,″-C2
H2-C3
H1-C3
H6-C7
H11-C12
H13′,″-C14
H11-C9
H7-C8
H6-C8
^{1}H (ppm)
^{13}C (ppm)

E

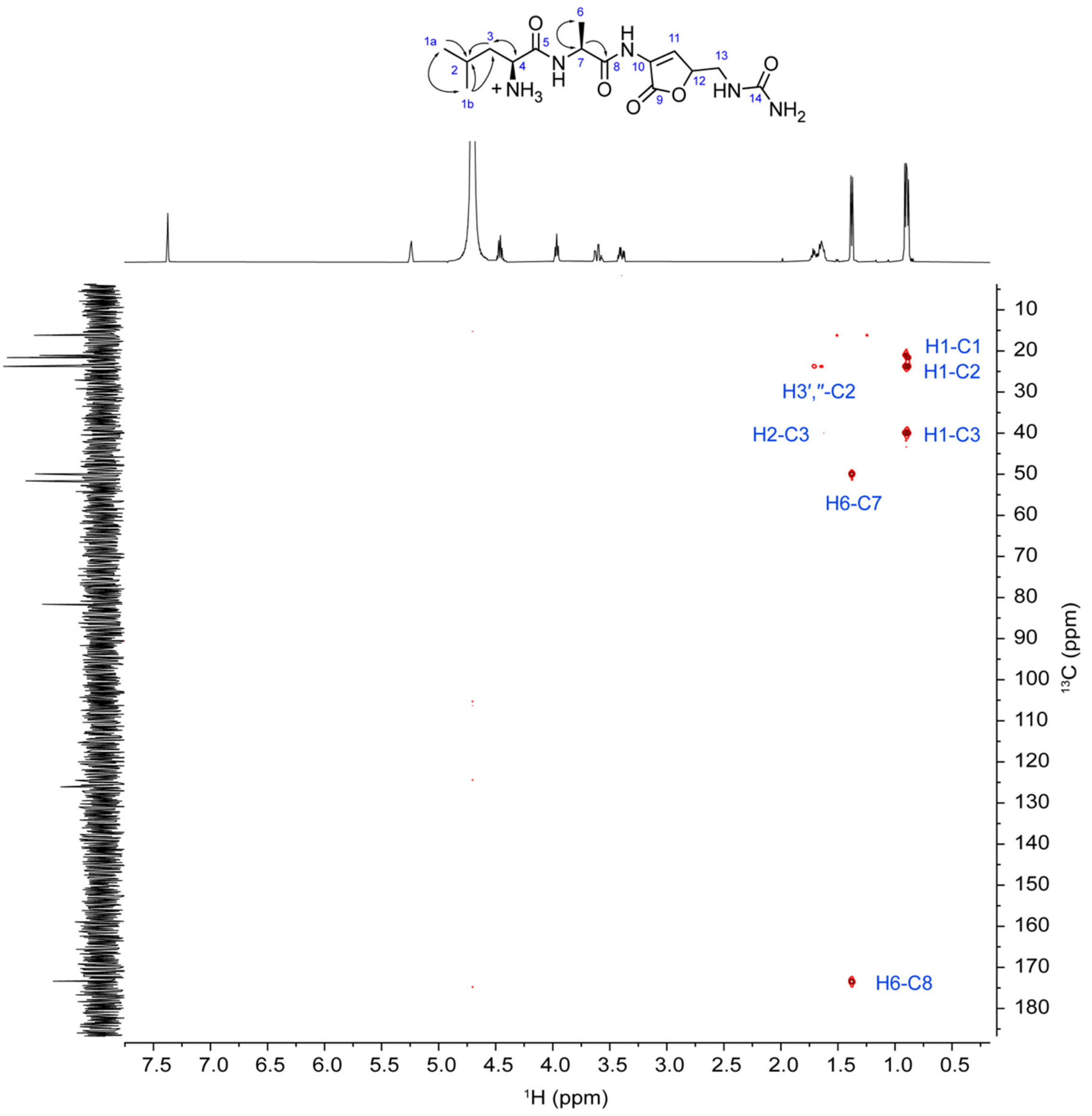

F

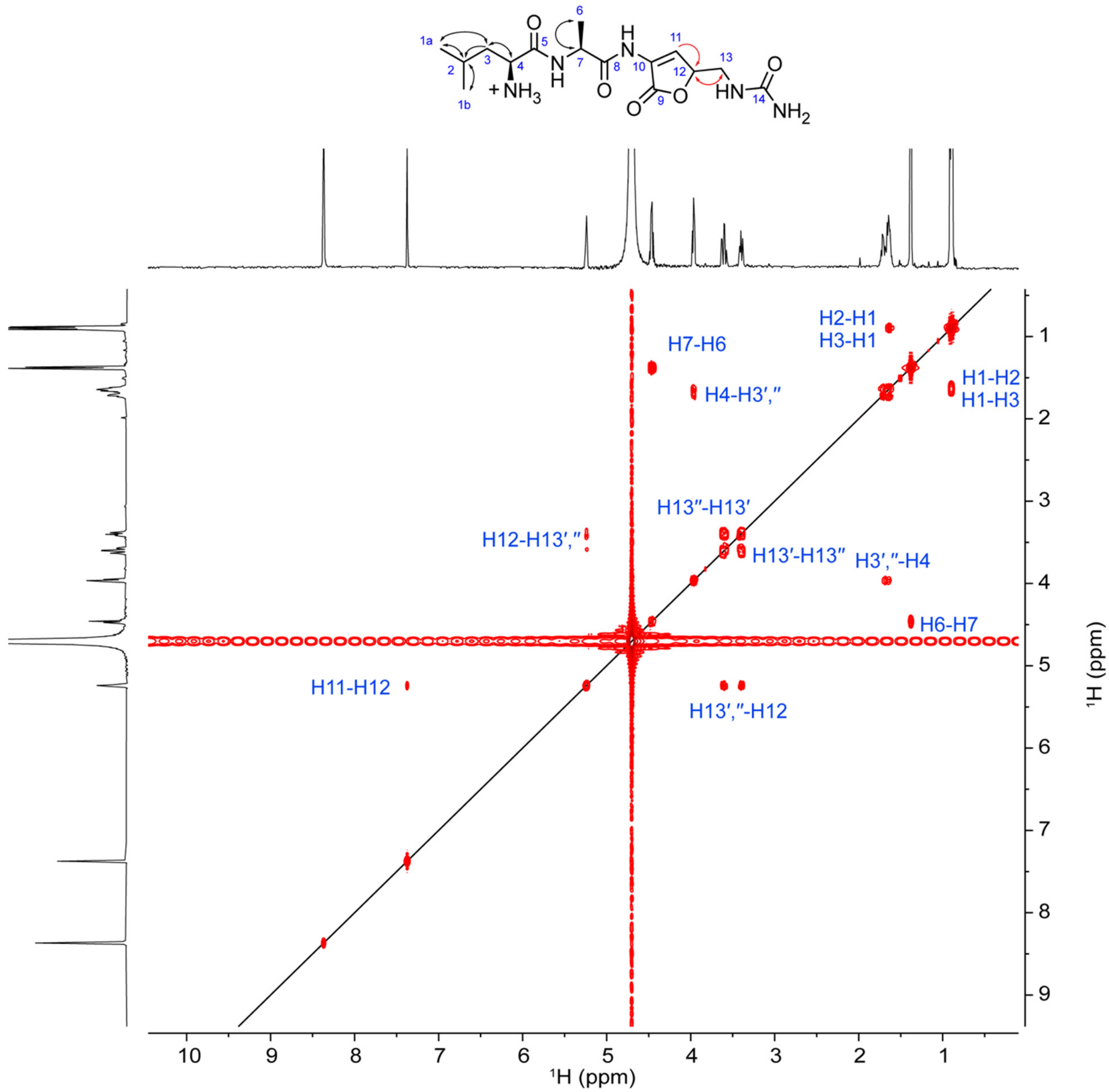
H2-H1
H3-H1
H7-H6
H4-H3′,″
H1-H2
H1-H3
H13″-H13′
H12-H13′,″
H13′-H13″
H3′,″-H4
H6-H7
H11-H12
H13′,″-H12
¹H (ppm)
¹H (ppm)

G

Atom	NMR Shift (ppm)
H1[a,b]	0.88, 0.90 (d)
H2	1.63 (m)
H3	1.63, 1.69 (m,m)
H4	4.47 (q)
H6	1.37 (d)
H7	3.95 (t)
H11	7.37 (m)
H12	5.22 (m)
H13′,″	3.40, 3.61 (td)
C1[a,b]	21.19, 21.77
C2	23.54
C3	40.26
C4	49.97
C5	173.57
C6	16.36
C7	51.68
C8	173.58
C9	170.62
C10	n.d.
C11	130.88
C12	81.67
C13	62.40
C14	161.34

H

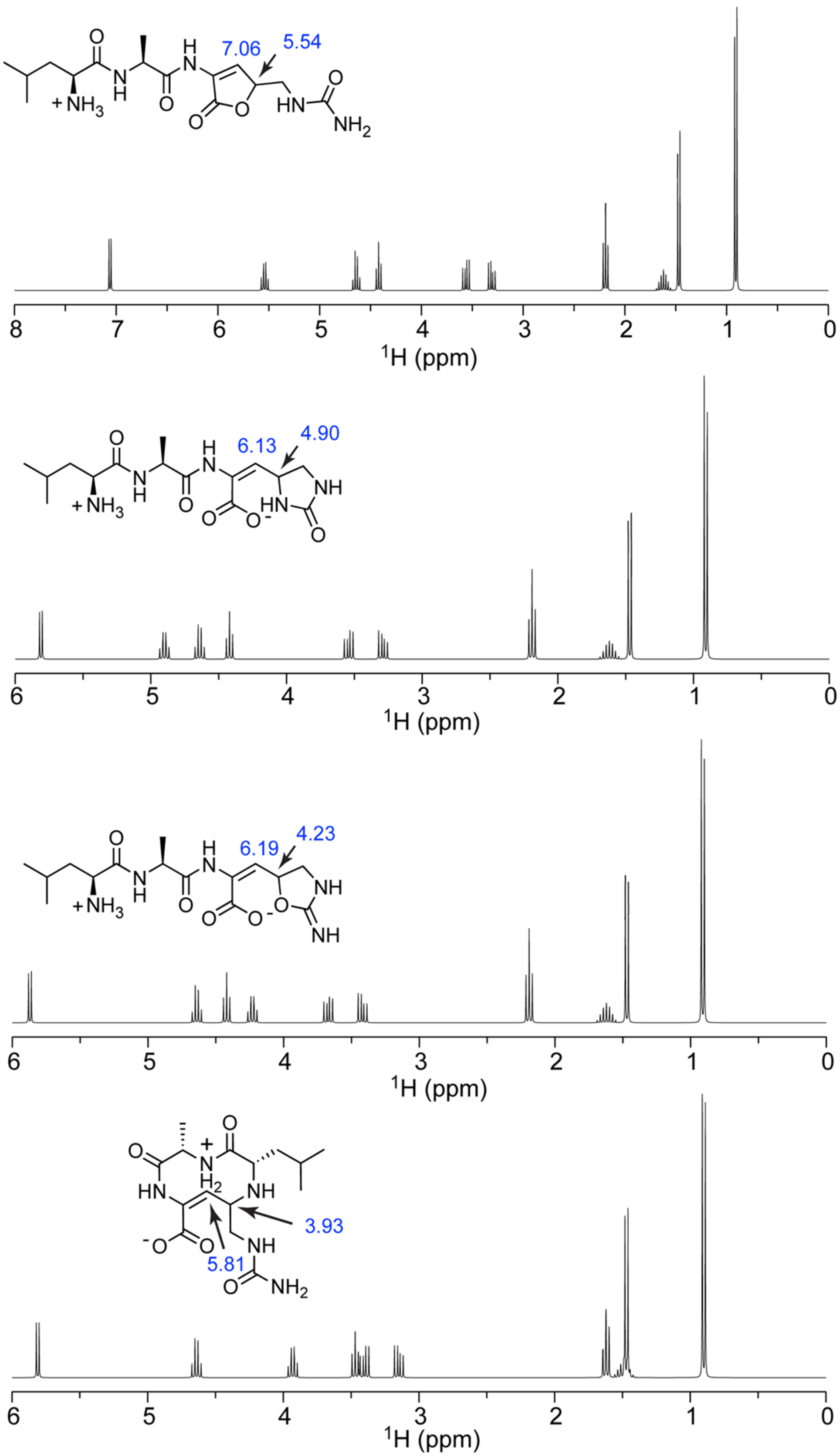

Table S2. Data collection and refinement parameters for apo MboA from *Pseudomonas syringae* (PDB ID 9YPI).

	MboA apo structure
**Data collection**	
Space group	P 43 21 2
Cell dimensions	
a, *b*, *c* (Å)	156.42, 156.42, 157.63
α, β, γ (°)	90, 90, 90
Resolution (Å)	43.61–2.49 (2.55–2.49)*
R_{sym} or R_{merge}	0.354 (2.495)
I / σI	11.0 (1.80)
Completeness (%)	95.0 (100.0)
Redundancy	26.98 (27.39)
$CC_{1/2}$	0.997 (0.505)
**Refinement**	
Resolution (Å)	43.61–2.49 (2.55–2.49)*
No. unique reflections	65471 (4774)
R_{work} / R_{free}	0.1870/0.2220
No. atoms	7906
Protein	7391
Ligand/ion	58
Water	457
B-factors	
Protein	50.92
Ligand/ion	89.47
Water	47.39
R.m.s. deviations	
Bond lengths (Å)	0.003
Bond angles (°)	0.51

*Values in parentheses are for highest-resolution shell.

Table S3. Data collection and refinement parameters for MboA from *Pseudomonas syringae* bound to the Leu-Ala-Arg tripeptide (PDB ID 9YPL).

	MboA with Leu-Ala-Arg peptide bound
**Data collection**	
Space group	P 43 21 2
Cell dimensions	
a, *b*, *c* (Å)	157.74, 157.74, 157.01
α, β, γ (°)	90, 90, 90
Resolution (Å)	43.75–2.20 (2.24–2.20)*
R_{sym} or R_{merge}	0.257 (2.284)
I / σI	13.5 (1.80)
Completeness (%)	100.0 (100.0)
Redundancy	27.64 (28.34)
$CC_{1/2}$	0.998 (0.634)
**Refinement**	
Resolution (Å)	43.75–2.20 (2.63–2.20)*
No. unique reflections	100535 (4924)
R_{work} / R_{free}	0.1705/0.1919
No. atoms	8203
Protein	7534
Ligand/ion	74
Water	595
B-factors	
Protein	39.05
Ligand/ion	81.54
LAR	36.5
Water	41.06
R.m.s. deviations	
Bond lengths (Å)	0.005
Bond angles (°)	0.70

*Values in parentheses are for highest-resolution shell.

Table S4. Data collection and refinement parameters for $Fe(II)_2$ MboA from *Pseudomonas syringae* bound to the Leu-Ala-Arg tripeptide (PDB ID 9YPM).

	$Fe(II)_2$ MboA with Leu-Ala-Arg peptide bound
**Data collection**	
Space group	P 43 21 2
Cell dimensions	
a, *b*, *c* (Å)	157.09, 157.09, 157.42
α, β, γ (°)	90, 90, 90
Resolution (Å)	90.76–2.45 (2.50–2.45)*
R_{sym} or R_{merge}	0.192 (3.043)
$I / \sigma I$	18.1 (1.40)
Completeness (%)	100.0 (100.0)
Redundancy	26.38 (27.05)
$CC_{1/2}$	0.999 (0.571)
**Refinement**	
Resolution (Å)	90.76–2.45 (2.50–2.45)*
No. unique reflections	72777 (4437)
R_{work} / R_{free}	0.1627/0.1951
No. atoms	8119
Protein	7526
Ligand/ion	323
Water	270
B-factors	
Protein	57.97
Ligand/ion	93.17
LAR	57.16
Fe	70.50
Water	60.11
R.m.s. deviations	
Bond lengths (Å)	0.003
Bond angles (°)	0.54

*Values in parentheses are for highest-resolution shell.

Figure S15. Electron density maps of Chain A and Chain B of Fe(II)$_2$ MboA bound to LAR. Different coordination at Fe1 observed Chain A and Chain B (also representative of Chains CD). (A) Composite F_o–F_c omit map contoured to 2.75 standard deviations from the mean for the active site in Chain A. Note that there is density for Asp90 bound to the Fe, but in Chain B it flips out of the active site and replaced by a water molecule. (B) POLDER [*28*] map (9 σ) for His89 in Chain A, which lacks strong density in the F_o–F_c omit map. Note that His89 populates two orientations at approximately a 1:1 ratio, one bound to the metal and one pointing away. (C) Composite F_o–F_c anomalous omit map (5 σ) for for Chain A calculated from the native wavelength data indicating heavy atoms. (A) Composite F_o–F_c omit map contoured to 2.75 standard deviations from the mean (σ) for the active site in Chain B. (D) POLDER [*28*] omit map for His89 (10 σ) in Chain A and the two metal-bound H_2Os (8 σ), which lacks strong density in the F_o–F_c omit map. (F) Composite F_o–F_c anomalous omit map (5 σ) for Chain B calculated from the native wavelength data indicating heavy atoms.

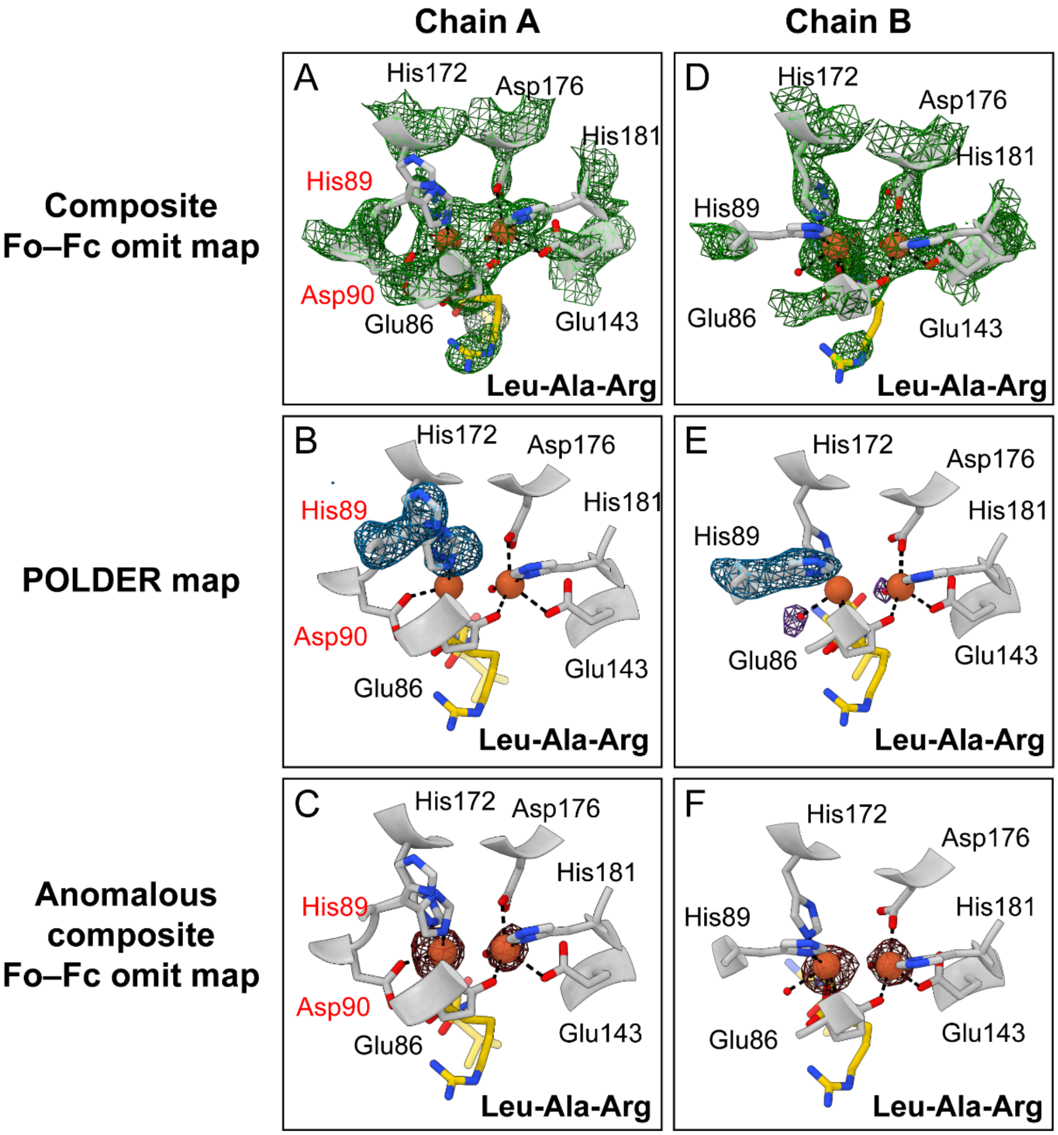

Figure S16. Comparison of the MboA to other crystallographically-characterized HDOs. The overall fold of MboA (PBD ID: 9YPI, apo) was compared to HrmI (PBD ID: 9N2A) [*40*], SznF (PBD ID: 6VZY) [*5*], UndA (PBD ID: 6P5Q) [*41*], AetD (PBD ID: 8TWW) [*8*], and BesC (PBD ID: 7TWA) [*42*]. Like AetD [*7, 8*] and UndA [*41*], MboA lacks a loop insertion between its metal ligands on the first core helix that exists in SznF [*5*], BesC [*42*], HrmI [*43*], and CADD [*44*]. We also note the C-terminal flexible loop that is only present in the core helix α3 of AetD [*8*]. For clarity, the partially modeled N-terminal His-tag was omitted from MboA and the additional domains present in HrmI and SznF were colored light gray.

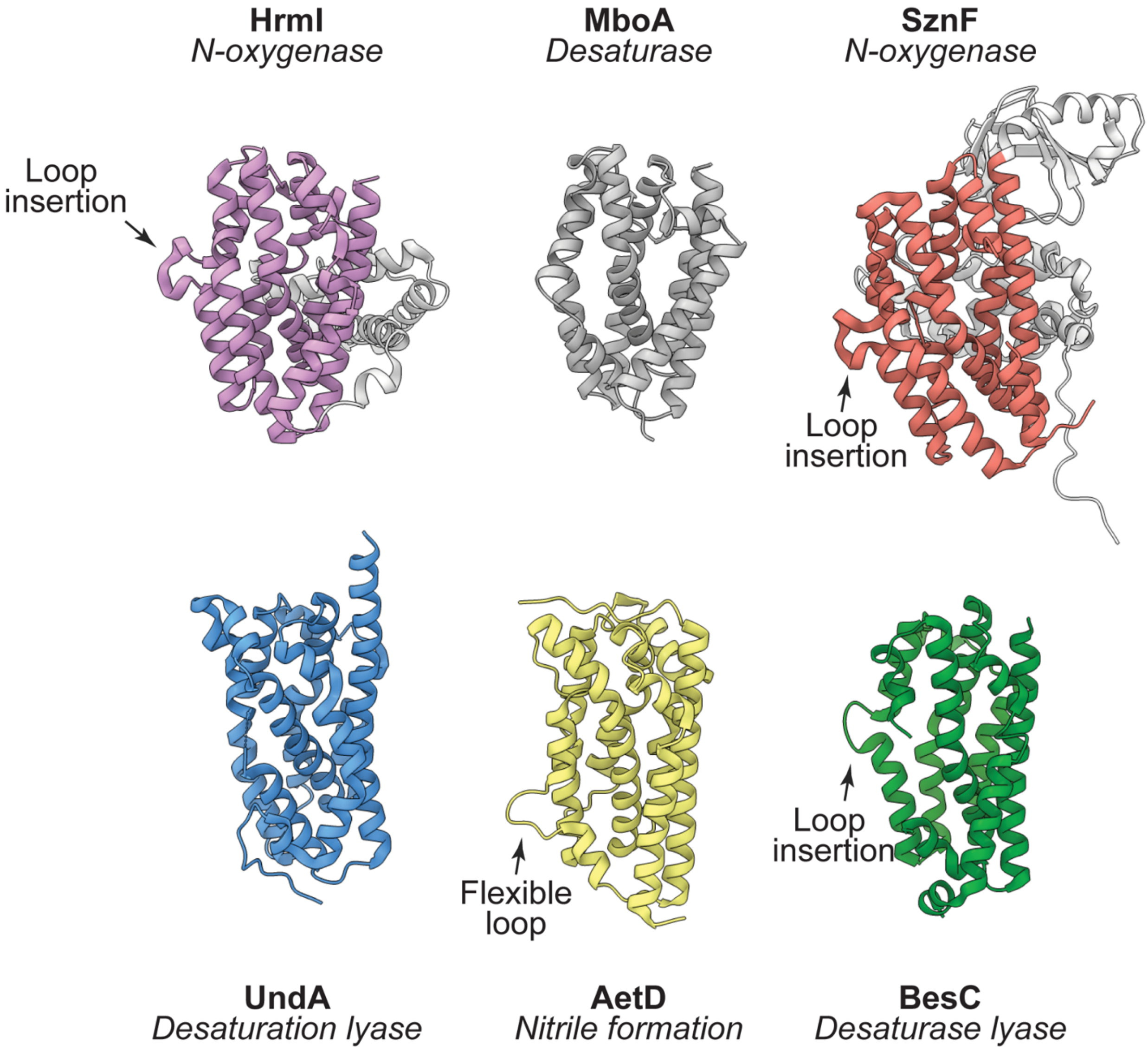

Figure S17. Comparison of the three MboA structures. (A) Overlay of apo MboA (PDB ID: 9YPI, purple), apo MboA bound to Leu-Ala-Arg (PDB ID: 9YPL, blue), and $Fe(II)_2$ MboA bound to Leu-Ala-Arg (PDB ID: 9YPM, Chain B, grey). Neither the presence of the assembled $Fe(II)_2$ active site nor LAR substrate significantly changes the overall fold of MboA overall (9YPI vs: 9YPL, 0.839 Å r.m.s.d.; 9YPM, 0.853 Å r.m.s.d.). Only subtle changes, such as the movement of Gly88 near the metal binding residue His89, are observed. (B) Overlay of the active sites of the three MboA structures, again highlighting the movement of Gly88 and the flipping of Glu143 to bind Fe2. Note that the position of Leu-Ala-Arg does not significantly change (RMSD: 1.01 Å) based on the presence of the metallocofactor despite chelation of the C-terminal carboxylate to Fe1. Waters were omitted for clarity.

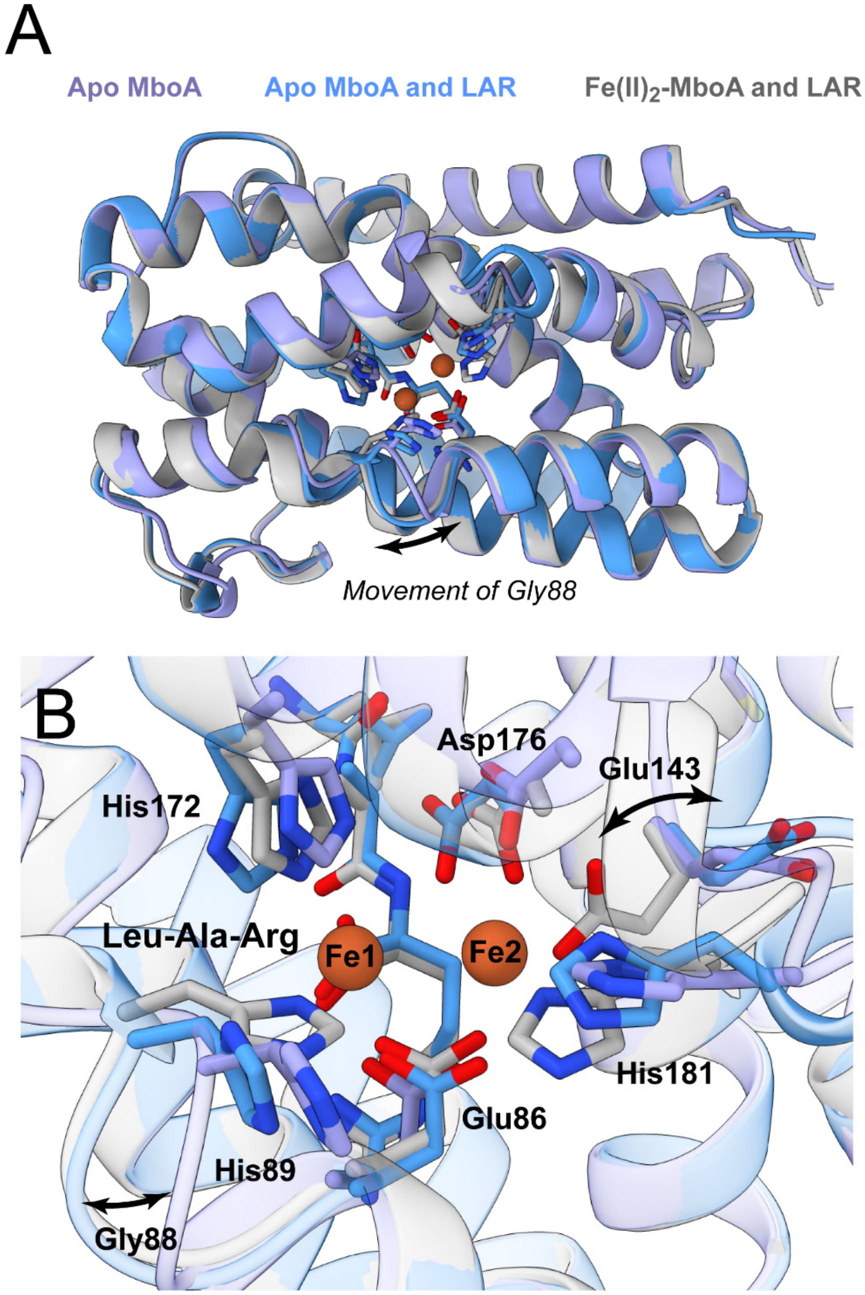

Figure S18. Channel and electrostatic analysis of MboA. (A) Two representations of the residues lining the MboA substrate channel (sticks, left; spheres, right). The radius of the spheres for protein residues was set to the van der Waals radius of each respective element. Analysis of the MboA structure (PDB ID: 9YPI) for channels was performed using MOLEonline [*45*]. (B) Analysis of the electrostatic potential map of the MboA structure (PDB ID: 9YPI) and the MboB AlphaFold model [*25*]. The bottom model shows the AlphaFold3 [*9*] MboAB heterodimer model and its electrostatic potential map. This model includes predicted complementary salt bridges from the positive residues in MboB and the acidic residues of MboA and gives the predicted localization of the N-terminal ferredoxin domain of MboB with respect to the active site of MboA. Electrostatic potential maps were calculated in ChimeraX [*29*].

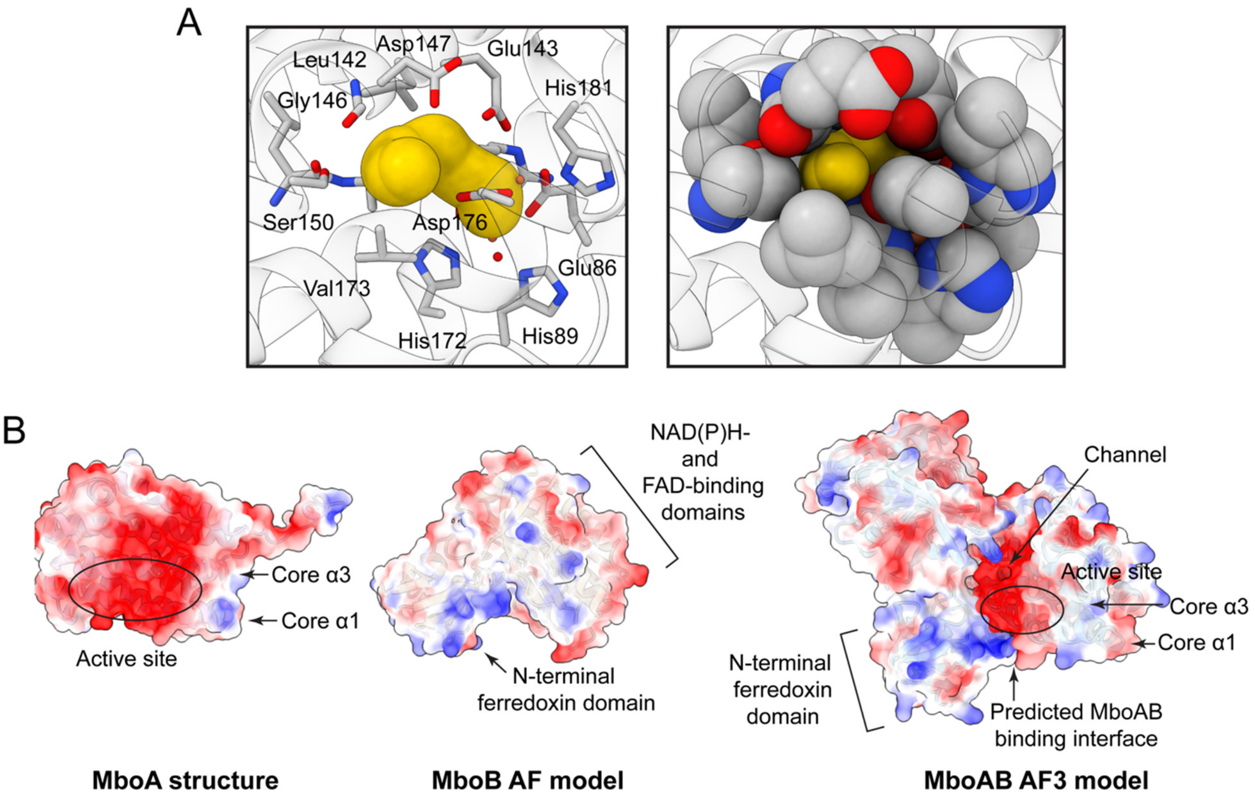

Figure S19. Sequence and structural analysis of the MboA coordination environment. The core α3 helix of HDOs typically contains a HX_3E/DX_2H motif that contributes three ligands to the Fe ions within the active site [*2*]. MboA exhibits a HX_3E/DX_2E motif and therefore we expected to observe a 2-His, 4-Glu/Asp coordination of the bimetallic cofactor, rather than the expected 3-His, 3-Glu/Asp coordination. However, despite a slight deviation in sequence, MboA still contributes all the ligands that are typical for HDOs (3-His, 3-Glu/Asp). As previously reported, there are likely many other HDOs that deviate from this sequence motif in sequence space while retaining the same coordination environment [*3*]. (A) Sequence alignment of MboA with other characterized enzymes from the HDO superfamily. Residues from the 3-His, 3-Glu/Asp metal-binding motif are highlighted in red. The observed metal-binding residues from the MboA structure are marked with an asterisk. (B) Overlay of the MboA active sites from Chain A (purple) and Chain B (grey) (PDB ID: 9YPM). In Chain A, Asp90 is bound to Fe1 and His89 occupies two orientations (bound to Fe1 at ~50% occupancy). The position of Gly88 is similar to apo MboA, which affects the positioning of Asp90. In Chain B, His89 is coordinated to Fe1 and Asp90 is replaced by a water molecule. (C) Logo plot of the residues neighboring the metal-binding residues (Asp86, Asp89, and Asp90) and the proposed base for desaturation (Tyr82). The logo plot was generated using WebLogo [*46*]. (D) Multiple sequence alignment of MboA, VarO, and a selection of sequences from a BLAST search of MboA on UniProt using ClustalO [*12*]. The black asterisk indicates metal-binding residues (including Asp90 of MboA) and the red asterisk indicates the proposed base for desaturation (Y82). The alignment is visualized using Unipro UGENE.

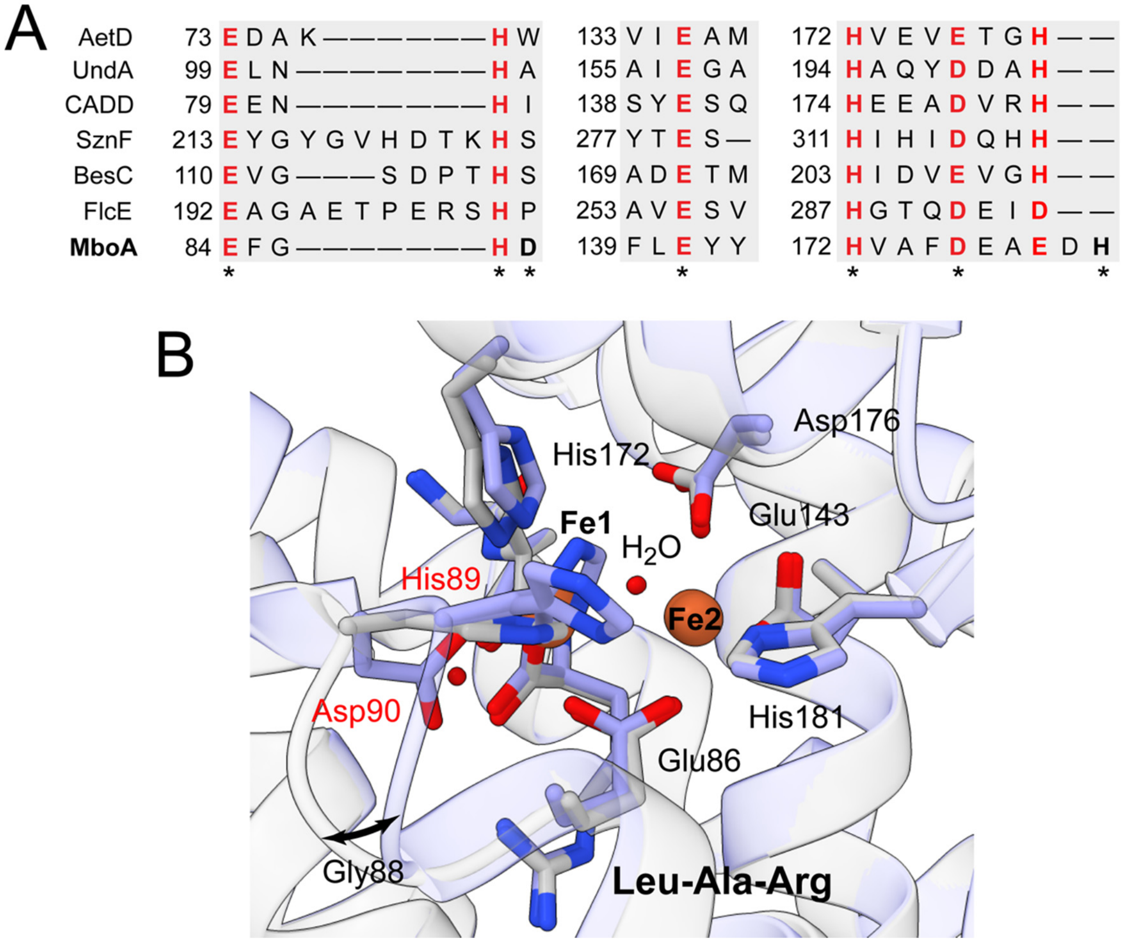

C

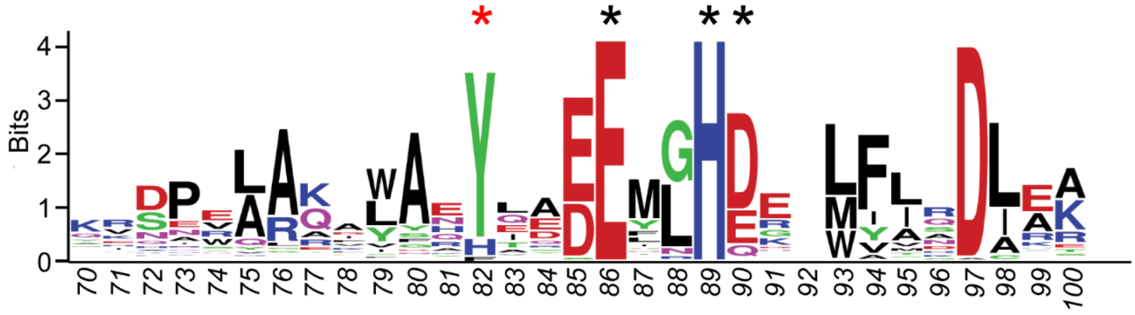

D

	70	71	72	73	74	75	76	77	78	79	80	81	82 *	83	84	85	86 *	87	88 *	89 *	90
**MboA|100.0%**	I	D	N	I	S	A	A	K	Q	L	S	Y	Y	L	Y	D	E	F	G	H	D
VarO|30.8%	Y	G	D	Y	R	L	A	G	K	L	A	R	Y	L	A	D	E	I	G	H	E
A0A3N4SJE5|36.3%	S	Q	D	D	S	L	A	A	K	L	A	R	Y	L	A	E	E	F	G	H	E
A0A931B5I4|36.6%	S	K	D	D	S	L	A	A	K	L	A	K	Y	L	A	E	E	F	G	H	E
A0A1S1PK62|36.8%	S	K	D	D	S	L	A	A	N	L	A	K	Y	L	A	E	E	F	G	H	E
A0A833JAZ0|40.7%	N	Q	D	N	I	L	A	K	D	F	T	Y	Y	L	Y	D	E	L	C	H	D
A0A561UCP7|34.1%	S	Q	D	D	V	L	A	A	K	L	A	R	Y	L	A	E	E	Y	G	H	E
A0A1N7SHX0|33.2%	F	K	D	N	H	L	A	Q	I	L	A	Q	Y	L	A	E	E	Y	G	H	E
B2JYA4|34.8%	Y	K	D	N	V	L	A	Q	F	L	A	R	Y	L	A	E	E	Y	G	H	D
A0A940SGX3|34.8%	Y	K	D	N	V	L	A	Q	F	L	A	R	Y	L	A	E	E	Y	G	H	D
A0A1H7E711|34.8%	Y	K	D	N	V	L	A	Q	F	L	A	R	Y	L	A	E	E	Y	G	H	D
A0A6J5DFA5|32.7%	F	K	D	N	P	I	A	Q	I	L	A	Q	Y	L	A	E	E	Y	G	H	E
F2LC77|32.3%	F	K	D	N	H	L	A	Q	I	L	A	Q	Y	L	A	E	E	Y	G	H	E
A0AA46AG02|32.9%	Y	S	D	N	K	L	A	Q	L	L	S	T	Y	L	A	E	E	F	G	H	D
A0A2I0SHC6|34.6%	Y	K	D	H	R	L	A	A	T	L	A	R	Y	L	S	E	E	N	N	H	E
A0A2N7WP51|32.7%	S	K	D	N	A	L	A	Q	T	L	A	Q	Y	L	S	E	E	Y	G	H	E
A0A1H9JVX3|34.1%	Y	K	D	H	R	L	A	A	T	L	A	R	Y	L	S	E	E	N	N	H	E
A0A419ZTE7|32.1%	F	K	D	N	T	L	A	Q	I	L	A	Q	Y	L	A	E	E	Y	G	H	E
A0A7X0HFD3|34.2%	Y	K	D	H	R	L	A	A	T	L	A	R	Y	L	S	E	E	N	N	H	E
A0A7Y0FZL1|33.6%	Y	K	D	H	R	L	A	A	T	L	A	R	Y	L	S	E	E	N	N	H	E
A0A931B1C4|28.0%	L	H	D	P	V	T	A	K	E	W	A	E	Y	T	D	D	E	M	L	H	D
A0A2N8NNA0|28.0%	L	H	D	P	V	A	A	K	E	W	A	E	Y	T	D	D	E	M	L	H	D

Figure S20. Comparison of substrate positioning in HDOs. Comparison of the MboA active site (PDB ID: 9YPM), AetD (PDB ID: 8TWW) [*7*], UndA (PDB ID: 6P5Q) [*41*], and HrmI (9N2A) [*40*] active sites and the positioning of the substrate with respect to Fe1 and Fe2. In MboA, the reactive site on the Arg side-chain of the LAR tripeptide is located between Fe1 and Fe2. In comparison, for AetD, UndA, and HrmI, the substrate is localized towards Fe1.

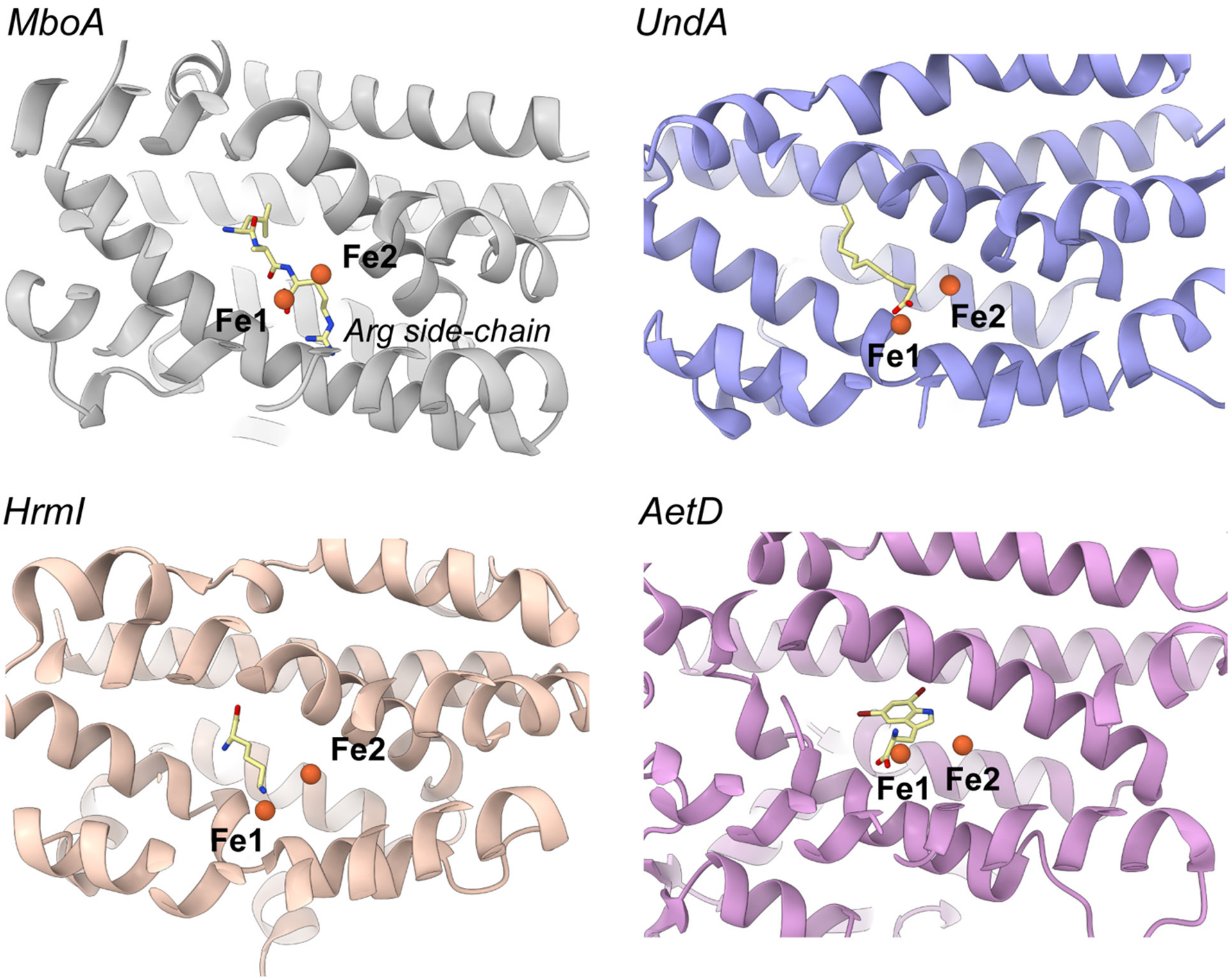

Figure S21. MboA active site geometry with the substrate C–H bonds modeled. ChimeraX was used to estimate the positions of the C–H bonds and measure the distances between H_β and the Fe2-bound water (2.1 Å) and between the H_γ and the Tyr82 phenol (2.5 Å) in MboA (PDB ID: 9YPM, Chain B).

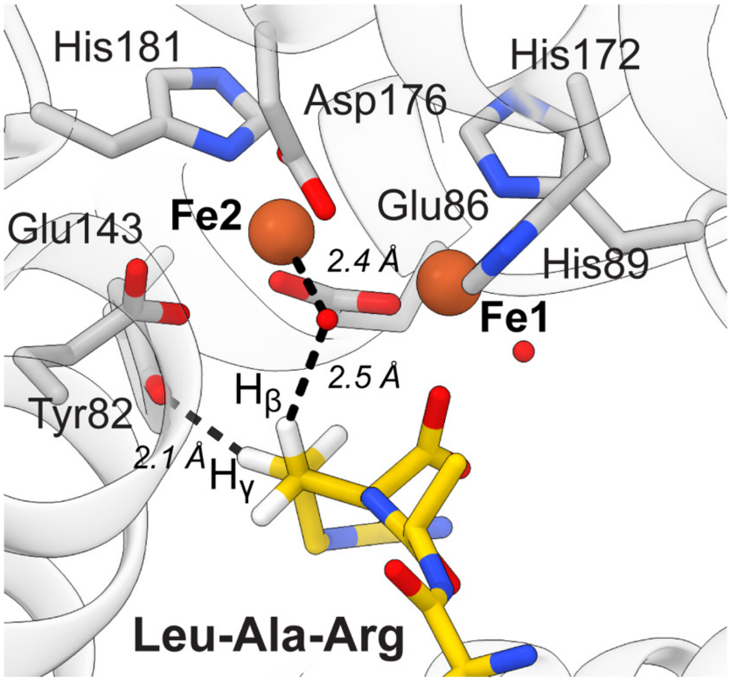

Figure S22. Analysis of the metal-metal distances in MboA and other binuclear metalloenzymes. (A) The Fe–O–O angles for the Fe(II)$_2$ cofactors of MboA (PDB ID: 9YPM), soluble methane monooxygenase (sMMO, PDB ID: 1FYZ) [*47*], SznF (PDB ID: 6VZY) [*5*], and AetD (PDB ID: 8TWW) [*8*]. The shorter Fe–Fe distance observed in MboA and sMMO seems to be correlated with both Fe sites possessing more acute Fe–O–O angles. (B) The Co–Co distances averaged from the five output models with Co(II) added from the AlphaFold3 server [*9*] are tabulated along with their experimentally-determined metal-metal distances from crystal structures. The crystallographic Fe–Fe distances are reported as the average of all chains if multiple chains are present within the structure. Notably, the average metal-metal distance in the MboA models consistently exhibit a shorter metal-metal distance than the other metal-bound HDO models (*, HrmI only has one chain in its crystal structure). (C) Representative AlphaFold models used in Panel B. (D) Representative resting, intermediate, and proposed intermediate states for FDOs [*48, 49*], HDOs [*2*], and membrane-bound FAD-like enzymes [*50, 51*].

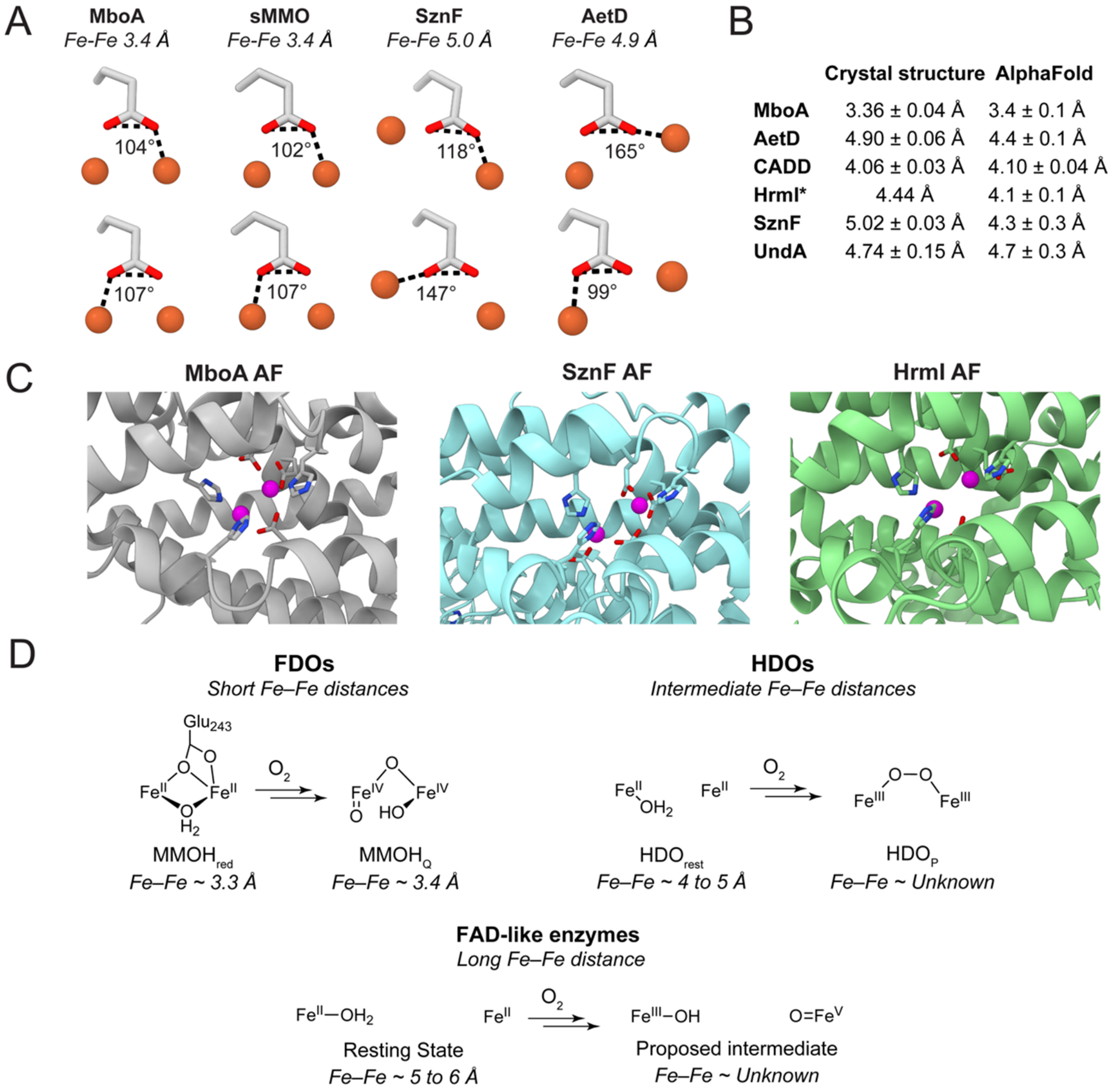

	Crystal structure	AlphaFold
**MboA**	3.36 ± 0.04 Å	3.4 ± 0.1 Å
**AetD**	4.90 ± 0.06 Å	4.4 ± 0.1 Å
**CADD**	4.06 ± 0.03 Å	4.10 ± 0.04 Å
**HrmI***	4.44 Å	4.1 ± 0.1 Å
**SznF**	5.02 ± 0.03 Å	4.3 ± 0.3 Å
**UndA**	4.74 ± 0.15 Å	4.7 ± 0.3 Å

Figure S23. Comparison of MboA with HDOs, fatty acid desaturases (FADs), fatty acid desaturase-like (FAD-like) enzymes, and sMMO. Based on sequence and structural comparisons, MboA is an acetylenase that is distinct from fatty acid desaturase (FAD)-like acetylenases. (A) Comparing the active site of MboA to sMMO (*M. capsulatus*, PDB ID: 1FYZ) [*47*], and a representative FAD (mouse stearoyl-CoA desaturase, PDB ID: 6WF2) [*50*]. (B) Sequence identity matrix MboA, HDOs, FADs, acetylenases, and AlkB. (C) Phylogenetic tree of the sequences shown in Panel B. The multiple sequence alignment was generated using ClustalO [*12*] (* and bolded, experimentally-characterized acetylenases).

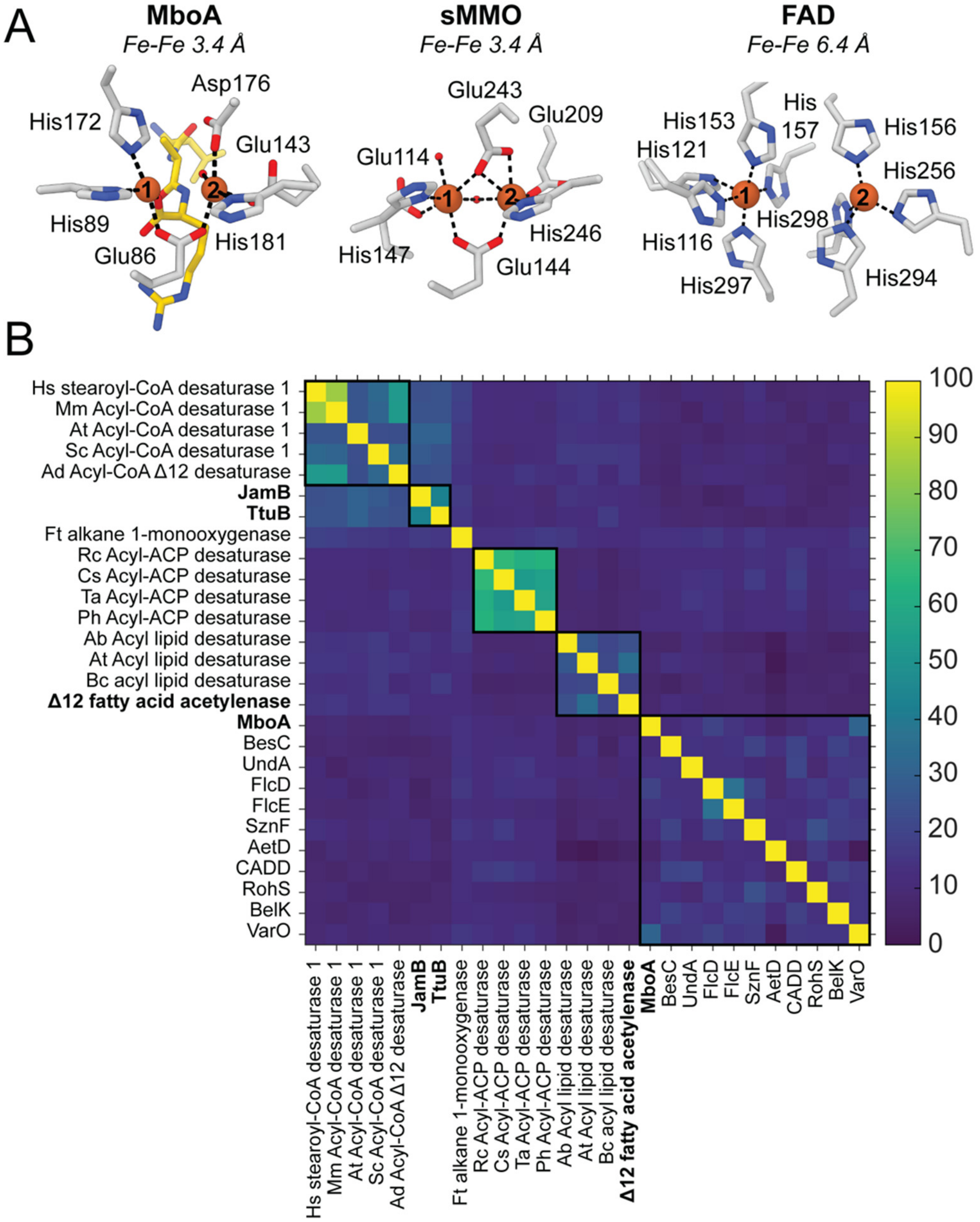

C

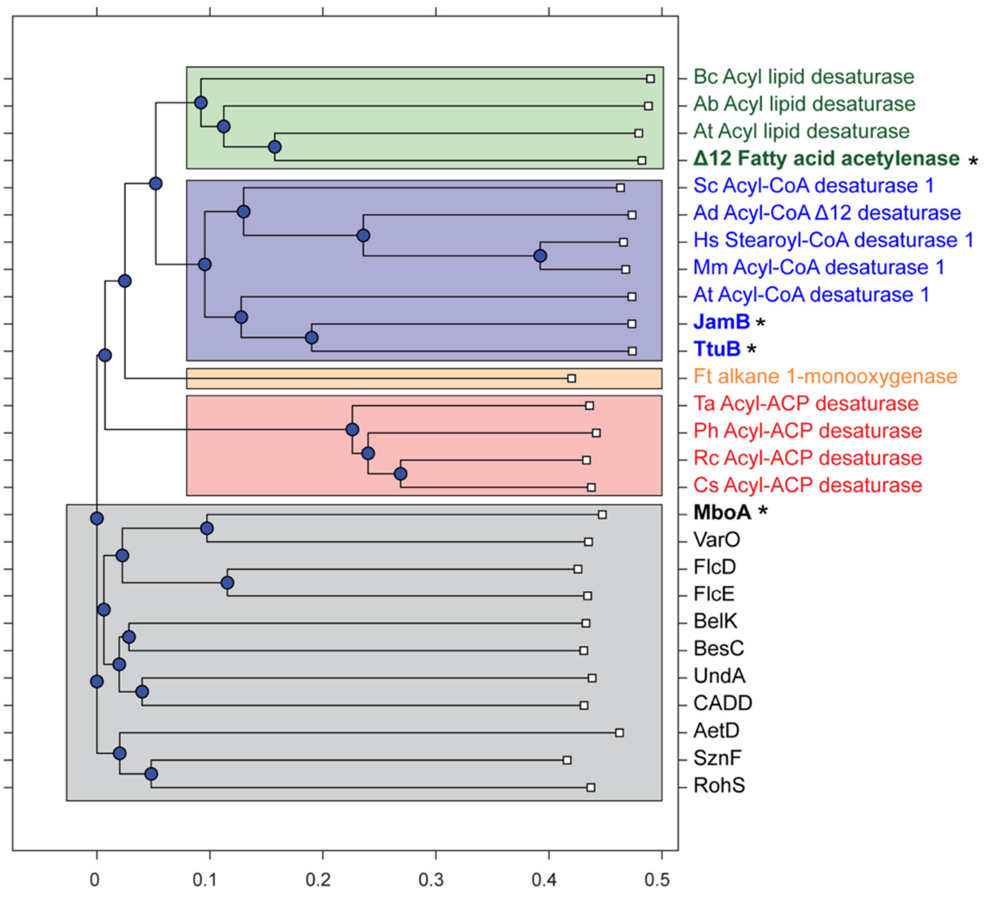
Bc Acyl lipid desaturase
Ab Acyl lipid desaturase
At Acyl lipid desaturase
Δ12 Fatty acid acetylenase *
Sc Acyl-CoA desaturase 1
Ad Acyl-CoA Δ12 desaturase
Hs Stearoyl-CoA desaturase 1
Mm Acyl-CoA desaturase 1
At Acyl-CoA desaturase 1
JamB *
TtuB *
Ft alkane 1-monooxygenase
Ta Acyl-ACP desaturase
Ph Acyl-ACP desaturase
Rc Acyl-ACP desaturase
Cs Acyl-ACP desaturase
MboA *
VarO
FlcD
FlcE
BelK
BesC
UndA
CADD
AetD
SznF
RohS
0
0.1
0.2
0.3
0.4
0.5

References

1. J. B. Broderick, B. R. Duffus, K. S. Duschene and E. M. Shepard, Radical *S*-adenosylmethionine enzymes. *Chem. Rev.* **2014**, *114* (8), 4229-4317.
2. S. R. Pope, M. J. McBride, M. A. Nair, X. E. Salas-Solá, C. Krebs, J. M. Bollinger and A. K. Boal, Heme oxygenase-like metalloenzymes. *Annu. Rev. Biochem.* **2025**, *94* (1), 59-88.
3. W. C. Simke, M. E. Walker, L. A. Calderone, A. T. Putz, J. B. Patteson, C. N. Vitro, C. F. Zizola, M. R. Redinbo, M.-E. Pandelia, T. L. Grove and B. Li, Structural basis for methine excision by a heme oxygenase-like enzyme. *ACS Cent. Sci.* **2024**, *10* (8), 1524-1536.
4. T. L. Ng, R. Rohac, A. J. Mitchell, A. K. Boal and E. P. Balskus, An *N*-nitrosating metalloenzyme constructs the pharmacophore of streptozotocin. *Nature* **2019**, *566* (7742), 94-99.
5. M. J. McBride, S. R. Pope, K. Hu, C. D. Okafor, E. P. Balskus, J. M. Bollinger and A. K. Boal, Structure and assembly of the diiron cofactor in the heme-oxygenase-like domain of the *N*-nitrosourea-producing enzyme SznF. *Proc. Natl. Acad. Sci. U.S.A.* **2021**, *118* (4), e2015931118.
6. Z. J. Wehrspan, R. T. McDonnell and A. H. Elcock, Identification of iron-sulfur (Fe-S) cluster and zinc (Zn) binding sites within proteomes predicted by DeepMind's AlphaFold2 program dramatically expands the metalloproteome. *J. Mol. Biol.* **2022**, *434* (2), 167377.
7. H. Li, J. W. Huang, L. Dai, H. Zheng, S. Dai, Q. Zhang, L. Yao, Y. Yang, Y. Yang, J. Min, R. T. Guo and C. C. Chen, The structural and functional investigation into an unusual nitrile synthase. *Nat. Commun.* **2023**, *14* (1), 7425.
8. S. Adak, N. Ye, L. A. Calderone, M. Duan, W. Lubeck, R. J. B. Schäfer, A. L. Lukowski, K. N. Houk, M. E. Pandelia, C. L. Drennan and B. S. Moore, A single diiron enzyme catalyses the oxidative rearrangement of tryptophan to indole nitrile. *Nat. Chem.* **2024**, *16* (12), 1989-1998.
9. J. Abramson, J. Adler, J. Dunger, R. Evans, T. Green, A. Pritzel, O. Ronneberger, L. Willmore, A. J. Ballard and J. Bambrick, Accurate structure prediction of biomolecular interactions with AlphaFold 3. *Nature* **2024**, *630* (8016), 493-500.
10. R. Gong, Y. Qu, J. Liu, X. Zhang, L. Zhou, Z. Tian, X. Zeng, B. Jin, Z. Li, L. Yu, R. Chen, Y. Zhou, L. Liao, L. Yang, X. Song, Y.-S. Cai, K. Shen, Z. Deng, Z. Zhang, B. Wang and W. Chen, A two-metalloenzyme cascade constructs the azetidine-containing pharmacophore. *Nat. Chem.* **2025**, doi: 10.1038/s41557-025-01949-y.
11. Y. Du, A. Thanapipatsiri, J. J. Blancas Cortez, X. E. Salas-Solá, C.-Y. Lin, A. K. Boal, C. Krebs, J. M. Bollinger and K. Yokoyama, Azetidine amino acid biosynthesis by non-haem iron-dependent enzymes. *Nat. Chem.* **2025**, doi: 10.1038/s41557-025-01958-x.
12. F. Sievers, A. Wilm, D. Dineen, T. J. Gibson, K. Karplus, W. Li, R. Lopez, H. McWilliam, M. Remmert, J. Söding, J. D. Thompson and D. G. Higgins, Fast, scalable generation of high-quality protein multiple sequence alignments using Clustal Omega. *Mol. Syst. Biol.* **2011**, *7*, 539.
13. N. Oberg, R. Zallot and J. A. Gerlt, EFI-EST, EFI-GNT, and EFI-CGFP: Enzyme Function Initiative (EFI) web resource for genomic enzymology tools. *J. Mol. Biol.* **2023**, *435* (14), 168018.

14. P. Shannon, A. Markiel, O. Ozier, N. S. Baliga, J. T. Wang, D. Ramage, N. Amin, B. Schwikowski and T. Ideker, Cytoscape: A software environment for integrated models of biomolecular interaction networks. *Genome Res.* **2003**, *13* (11), 2498-504.

15. J. A. Marchand, M. E. Neugebauer, M. C. Ing, C. I. Lin, J. G. Pelton and M. C. Y. Chang, Discovery of a pathway for terminal-alkyne amino acid biosynthesis. *Nature* **2019**, *567* (7748), 420-424.

16. D. G. Gibson, L. Young, R.-Y. Chuang, J. C. Venter, C. A. Hutchison and H. O. Smith, Enzymatic assembly of DNA molecules up to several hundred kilobases. *Nat. Methods* **2009**, *6* (5), 343-345.

17. E. Gasteiger, A. Gattiker, C. Hoogland, I. Ivanyi, R. D Appel and A. Bairoch, ExPASy: The proteomics server for in-depth protein knowledge and analysis. *Nucleic Acids Res.* 2003, *31*, 3784–3788.

18. M. M. Bradford, A rapid and sensitive method for the quantitation of microgram quantities of protein utilizing the principle of protein-dye binding. *Anal. Biochem.* **1976**, *72*, 248-54.

19. H. Tsugawa, T. Cajka, T. Kind, Y. Ma, B. Higgins, K. Ikeda, M. Kanazawa, J. VanderGheynst, O. Fiehn and M. Arita, MS-DIAL: Data-independent MS/MS deconvolution for comprehensive metabolome analysis. *Nat. Methods* **2015**, *12* (6), 523-6.

20. E. Arrebola, F. M. Cazorla, V. E. Durán, E. Rivera, F. Olea, J. C. Codina, A. Pérez-García and A. de Vicente, Mangotoxin: A novel antimetabolite toxin produced by *Pseudomonas syringae* inhibiting ornithine/arginine biosynthesis. *Physiol. Mol. Plant Pathol.* **2003**, *63* (3), 117-127.

21. V. J. Carrión, E. Arrebola, F. M. Cazorla, J. Murillo and A. de Vicente, The *mbo* operon Is specific and essential for biosynthesis of mangotoxin in *Pseudomonas syringae*. *PLOS ONE* **2012**, *7* (5), e36709.

22. W. Kabsch, XDS. *Acta. Crystallogr. D Biol. Crystallogr.* **2010**, *66* (Pt 2), 125-32.

23. P. R. Evans and G. N. Murshudov, How good are my data and what is the resolution? *Acta. Crystallogr. D. Biol Crystallogr.* **2013**, *69* (Pt 7), 1204-14.

24. M. D. Winn, C. C. Ballard, K. D. Cowtan, E. J. Dodson, P. Emsley, P. R. Evans, R. M. Keegan, E. B. Krissinel, A. G. Leslie, A. McCoy, S. J. McNicholas, G. N. Murshudov, N. S. Pannu, E. A. Potterton, H. R. Powell, R. J. Read, A. Vagin and K. S. Wilson, Overview of the CCP4 suite and current developments. *Acta. Crystallogr. D Biol. Crystallogr.* **2011**, *67* (Pt 4), 235-42.

25. J. Jumper, R. Evans, A. Pritzel, T. Green, M. Figurnov, O. Ronneberger, K. Tunyasuvunakool, R. Bates, A. Žídek, A. Potapenko, A. Bridgland, C. Meyer, S. A. A. Kohl, A. J. Ballard, A. Cowie, B. Romera-Paredes, S. Nikolov, R. Jain, J. Adler, T. Back, S. Petersen, D. Reiman, E. Clancy, M. Zielinski, M. Steinegger, M. Pacholska, T. Berghammer, S. Bodenstein, D. Silver, O. Vinyals, A. W. Senior, K. Kavukcuoglu, P. Kohli and D. Hassabis, Highly accurate protein structure prediction with AlphaFold. *Nature* **2021**, *596* (7873), 583-589.

26. P. Emsley, B. Lohkamp, W. G. Scott and K. Cowtan, Features and development of Coot. *Acta. Crystallogr. D Biol. Crystallogr.* **2010**, *66* (Pt 4), 486-501.

27. P. D. Adams, P. V. Afonine, G. Bunkóczi, V. B. Chen, I. W. Davis, N. Echols, J. J. Headd, L. W. Hung, G. J. Kapral, R. W. Grosse-Kunstleve, A. J. McCoy, N. W. Moriarty, R. Oeffner, R. J. Read, D. C. Richardson, J. S. Richardson, T. C. Terwilliger and P. H. Zwart, PHENIX: A comprehensive Python-based system for macromolecular structure solution. *Acta. Crystallogr. D. Biol. Crystallogr.* **2010**, *66* (Pt 2), 213-21.

28. D. Liebschner, P. V. Afonine, N. W. Moriarty, B. K. Poon, O. V. Sobolev, T. C. Terwilliger and P. D. Adams, Polder maps: Improving OMIT maps by excluding bulk solvent. *Acta. Crystallogr. D. Struct. Biol.* **2017**, *73* (Pt 2), 148-157.
29. E. C. Meng, T. D. Goddard, E. F. Pettersen, G. S. Couch, Z. J. Pearson, J. H. Morris and T. E. Ferrin, UCSF ChimeraX: Tools for structure building and analysis. *Protein Sci.* **2023**, *32* (11), e4792.
30. T. D. Goddard, C. C. Huang, E. C. Meng, E. F. Pettersen, G. S. Couch, J. H. Morris and T. E. Ferrin, UCSF ChimeraX: Meeting modern challenges in visualization and analysis. *Protein Sci.* **2018**, *27* (1), 14-25.
31. K. Kiianitsa, J. A. Solinger and W.-D. Heyer, NADH-coupled microplate photometric assay for kinetic studies of ATP-hydrolyzing enzymes with low and high specific activities. *Anal. Biochem.* **2003**, *321* (2), 266-271.
32. S. J. B. Mallinson, M. M. Machovina, R. L. Silveira, M. Garcia-Borràs, N. Gallup, C. W. Johnson, M. D. Allen, M. S. Skaf, M. F. Crowley, E. L. Neidle, K. N. Houk, G. T. Beckham, J. L. DuBois and J. E. McGeehan, A promiscuous cytochrome P450 aromatic *O*-demethylase for lignin bioconversion. *Nat. Commmun.* **2018**, *9* (1), 2487.
33. A. Karlsson, Z. M. Beharry, D. Matthew Eby, E. D. Coulter, E. L. Neidle, D. M. Kurtz, H. Eklund and S. Ramaswamy, X-ray crystal structure of benzoate 1,2-dioxygenase reductase from *Acinetobacter* sp. Strain ADP1. *J. Mol. Biol.* **2002**, *318* (2), 261-272.
34. J. F. Acheson, H. Moseson and B. G. Fox, Structure of T4moF, the toluene 4-monooxygenase ferredoxin oxidoreductase. *Biochemistry* **2015**, *54* (38), 5980-5988.
35. Y. Ashikawa, Z. Fujimoto, K. Inoue, H. Yamane and H. Nojiri, Crystal structure of the ferredoxin reductase component of carbazole 1,9a-dioxygenase from *Janthinobacterium* sp. J3. *Acta Crystallogr. D Struct. Biol.* **2021**, *77* (Pt 7), 921-932.
36. J. Müller, A. A. Lugovskoy, G. Wagner and S. J. Lippard, NMR structure of the [2Fe-2S] ferredoxin domain from soluble methane monooxygenase reductase and interaction with Its hydroxylase. *Biochemistry* **2002**, *41* (1), 42-51.
37. L. L. Chatwood, J. Müller, J. D. Gross, G. Wagner and S. J. Lippard, NMR structure of the flavin domain from soluble methane monooxygenase reductase from *Methylococcus capsulatus (Bath)*. *Biochemistry* **2004**, *43* (38), 11983-11991.
38. M. Barfield and B. Chakrabarti, Long-range proton spin-spin coupling. *Chem. Rev.* **1969**, *69* (6), 757-778.
39. E. Pretsch, P. Bühlmann and M. Badertscher, Structure determination of organic compounds. Springer Berlin, Heidelberg: 2009.
40. S. S. Skirboll, M. Gangopadhyay, H. N. Phan, J. Hartsell, A. Mudireddy, D. Hilovsky, P. D. Swartz, X. Liu, Y. Guo and T. M. Makris, The heme oxygenase-like diiron enzyme HrmI reveals altered regulatory mechanisms for dioxygen activation and substrate *N*-oxygenation. *J. Am. Chem. Soc.* **2025**, *147* (33), 30210-30221.
41. B. Zhang, L. J. Rajakovich, D. Van Cura, E. J. Blaesi, A. J. Mitchell, C. R. Tysoe, X. Zhu, B. R. Streit, Z. Rui, W. Zhang, A. K. Boal, C. Krebs and J. M. Bollinger, Jr., Substrate-triggered formation of a peroxo-Fe_2(III/III) intermediate during fatty acid decarboxylation by UndA. *J. Am. Chem. Soc.* **2019**, *141* (37), 14510-14514.

42. M. J. McBride, M. A. Nair, D. Sil, J. W. Slater, M. E. Neugebauer, M. C. Y. Chang, A. K. Boal, C. Krebs and J. M. Bollinger, Jr., Substrate-triggered µ-peroxodiiron(III) intermediate in the 4-chloro-l-Lysine-fragmenting heme-oxygenase-like diiron oxidase (HDO) BesC: Substrate dissociation from, and C4 targeting by, the intermediate. *Biochem.* **2022**, *61* (8), 689-702.

43. L. Pang, W. Niu, Y. Duan, L. Huo, A. Li, J. Wu, Y. Zhang, X. Bian and G. Zhong, *In vitro* characterization of a nitro-forming oxygenase involved in 3-(trans-2′-aminocyclopropyl)alanine biosynthesis. *Eng. Microbiol.* **2022**, *2* (1), 100007.

44. H. N. Phan, P. D. Swartz, M. Gangopadhyay, Y. Guo, A. I. Smirnov and T. M. Makris, Assembly of a heterobimetallic Fe/Mn cofactor in the para-aminobenzoate synthase Chlamydia protein associating with death domains (CADD) initiates long-range radical hole-hopping. *Biochemistry* **2024**, *63* (22), 3020-3029.

45. L. Pravda, D. Sehnal, D. Toušek, V. Navrátilová, V. Bazgier, K. Berka, R. Svobodová Vařeková, J. Koča and M. Otyepka, MOLEonline: A web-based tool for analyzing channels, tunnels and pores (2018 update). *Nucleic Acids Res.* **2018**, *46* (W1), W368-W373.

46. G. E. Crooks, G. Hon, J. M. Chandonia and S. E. Brenner, WebLogo: A sequence logo generator. *Genome Res.* **2004**, *14* (6), 1188-1190.

47. D. A. Whittington and S. J. Lippard, Crystal structures of the soluble methane monooxygenase hydroxylase from *Methylococcus capsulatus (Bath)* demonstrating geometrical variability at the dinuclear iron active site. *J. Am. Chem. Soc.* **2001**, *123* (5), 827-838.

48. G. E. Cutsail, III, R. Banerjee, A. Zhou, L. Que, Jr., J. D. Lipscomb and S. DeBeer, High-resolution extended X-ray absorption fine structure analysis provides evidence for a longer Fe···Fe Distance in the Q intermediate of methane monooxygenase. *J. Am. Chem. Soc.* **2018**, *140* (48), 16807-16820.

49. C. E. Schulz, R. G. Castillo, D. A. Pantazis, S. DeBeer and F. Neese, Structure–spectroscopy correlations for intermediate Q of soluble methane monooxygenase: Insights from QM/MM calculations. *J. Am. Chem. Soc.* **2021**, *143* (17), 6560-6577.

50. J. Shen, G. Wu, A.-L. Tsai and M. Zhou, Structure and mechanism of a unique diiron center in mammalian stearoyl-CoA desaturase. *J. Mol. Biol.* **2020**, *432* (18), 5152-5161.

51. J. T. Groves, L. Feng and R. N. Austin, Structure and function of alkane monooxygenase (AlkB). *Acc. Chem. Res.* **2023**, *56* (24), 3665-3675.
